# Breaking the antimicrobial resistance dilemma: marine natural products, a treasure trove for the development of new anti-*Staphylococcus aureus* drugs

**DOI:** 10.1007/s42995-026-00355-8

**Published:** 2026-02-02

**Authors:** Peng-Jie Li, Liu-Xia Lv, Xi-Zhen Cao, Jian-Yu Liu, Ji-Xiu Gao, Hao-Ran Li, Gulab Said, Dan Chen, Bo Yao, Xin Liu, Mei-Yan Wei, Xiao-Ping Yang, Tong-Yi Xu, Yu-Cheng Gu, Chang-Lun Shao

**Affiliations:** 1 Key Laboratory of Marine Drugs, The Ministry of Education of China, School of Medicine and Pharmacy, Ocean University of China, Qingdao, 266003 China; 2 Department of Chemistry, Women University Swabi, Swabi, 23430 Pakistan; 3 Hospital of Ocean University of China, Qingdao, 266003 China; 4 Department of Critical Care Medicine, Qilu Hospital (Qingdao), Cheeloo College of Medicine, Shandong University, Qingdao, 266035 China; 5 Science and Technology Research Center of Chinese Customs, Beijing, 100026 China; 6 School of Bioengineering, Beijing Polytechnic University, Beijing, 100176 China; 7 Qingdao Traditional Chinese Medicine Hospital, Qingdao Hiser Hospital Affiliated of Qingdao University, Qingdao, 266100 China; 8 Department of Cardiovascular and Thoracic Surgery, No. 971 Hospital of PLA Navy, Qingdao, 266071 China; 9 Syngenta Jealott’s Hill International Research Centre, Bracknell, Berkshire, RG42 6EY UK; 10 Key Laboratory of Tropical Medicinal Resource Chemistry of Ministry of Education, College of Chemistry and Chemical Engineering, Hainan Normal University, Haikou, 571158 China

**Keywords:** Antimicrobial resistance, *Staphylococcus aureus*, Drug-resistance mechanisms, Marine natural products

## Abstract

Antimicrobial resistance (AMR) affects multiple fields, including medicine, pharmacy, food safety, and animal husbandry, and poses a major threat to public health and economic development. *Staphylococcus aureus* has emerged as one of the most critical bacterial pathogens due to its widespread prevalence and significant genetic plasticity. This review outlines currently approved small-molecule drugs for clinical use and their mechanisms in controlling bacterial infections and antimicrobial resistance, highlighting the development of novel agents as an effective strategy to combat AMR. Special emphasis is placed on the antibacterial activity of marine-derived natural products (MNPs) against *S. aureus*, and a systematic analysis is presented for the first time on the structures, origins, activities, and structure–activity relationships of 1,192 compounds reported in 468 publications from 1977 to 2025. Among these, 36 MNPs exhibited significant activity against both methicillin-sensitive *S. aureus* and methicillin-resistant *S. aureus* (MIC ≤ 1 µg/mL). Macrolides and peptides represented the most prevalent structural classes, with compounds displaying potent activity accounting for 24% and 27% of the total active compounds, respectively. Further drug-likeness analysis shows that MNPs have promising therapeutic potential. Among the 160 highly active MNPs, over 80% have suitable lipid-water partition coefficients, and 41% fully meet Lipinski's Rule of Five. Structural analysis reveals tetrahydrofuran, tetrahydropyran, and sugar units as the most common motifs, present in 20, 16, and 14 compounds, respectively. Technologies and strategies to accelerate the development of marine-derived antibacterial drugs are also proposed. This will serve stakeholders across diverse sectors by supporting cross-sectoral coordination to address AMR.

**Supplementary Information:**

The online version contains supplementary material available at https://doi.org/10.1007/s42995-026-00355-8.

## Introduction

### The global situation of drug resistance

**Fig. 1:**
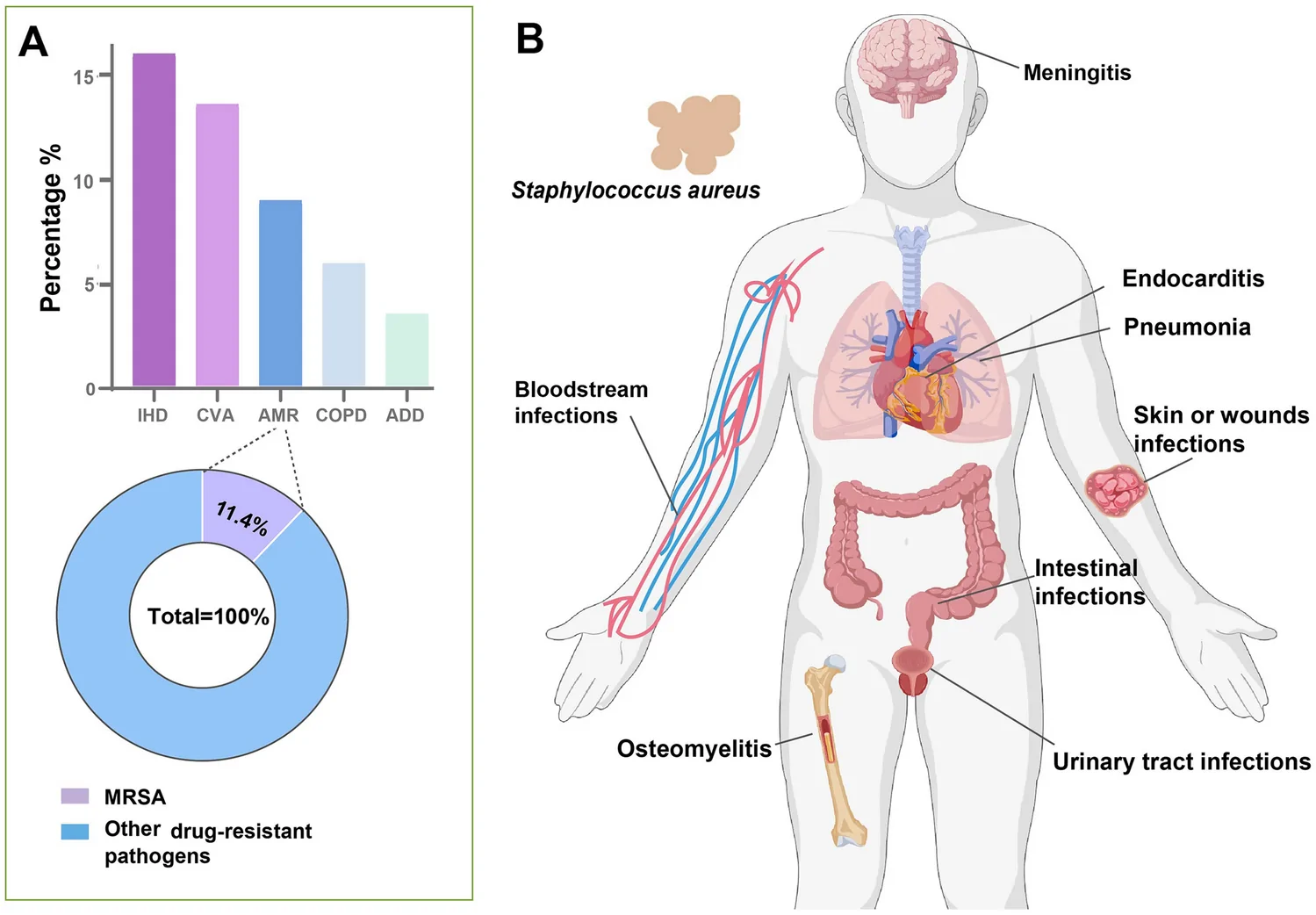
Current status of antimicrobial resistance and infections caused by *Staphylococcus aureus*. **A** AMR was among the five leading causes of death worldwide in 2021. AMR, Antimicrobial resistance; IHD, Ischemic Heart Disease; CVA, Cerebrovascular Accident; COPD, chronic obstructive pulmonary disease; ADD, Alzheimer's disease and other dementias. **B** *S. aureus* can cause severe infections in nearly all body organs

Antimicrobial resistance (AMR) has become a huge threat to human health. In 2021, the number of deaths related to antibiotic resistance was approximately 4.71 million, accounting for about 7% of the global death toll that year and was one of the five leading causes of death worldwide in 2021 (Fig. 1A) (World Health Organization. 2024a). In addition, the report by the US Centers for Disease Control and Prevention in 2024 shows that bacterial resistance is gradually increasing, and overall antibiotic resistance has increased by 20% from 2019 to 2022 (David 2024). It is estimated that the number of deaths related to drug-resistant bacteria will reach 8.22 million, of which about 1.91 million will be directly caused by resistance in 2050 (Miryam 2024; Naghavi et al. 2024).

AMR is a cross-cutting issue in global health, and the global rise of antibiotic resistance is not limited to human society. Antibiotic-resistant bacteria can also be observed in various fields such as agriculture, animal husbandry, and food production (Hernando-Amado et al. 2019; Monger et al. 2021; Scaccia et al. 2021; Zheng et al. 2024). In intensive farming, the use of antibiotics can address bacterial infections, but long-term and excessive antibiotic use has led to increasingly frequent occurrences of antibiotic-resistant strains in livestock environments and products such as pigs and fish (Ferri et al. 2022; Monger et al. 2021). In agricultural production, antibiotic resistance is equally severe. Fertilizers containing residual antibiotics, as well as sewage and wastewater used for irrigation, increase the abundance of antibiotic resistance genes in the soil, leading to the transfer of these genes to crops (Fusaro et al. 2024; He et al. 2020; Wang et al. 2020; Zhu et al. 2017). More critically, these resistant bacteria and resistance genes enter the human body through various pathways such as the food chain and air inhalation, becoming one of the reasons for the growing prevalence of clinical antibiotic resistance (He et al. 2020; Kurćubić et al. 2025).

*Staphylococcus aureus* (*S. aureus*) is a Gram-positive bacterium and a classic opportunistic pathogen, which can cause various infectious diseases such as skin or soft tissue infections, endocarditis, and pneumonia by reproducing in large quantities or invading the blood through the damaged body parts (Fig. 1B) (Keim and Horswill 2023). Over the past 30 years, the proportion of deaths caused by drug-resistant *S. aureus* has increased year by year, becoming one of the main growth points of bacterial resistance. Methicillin-resistant *Staphylococcus aureus* (MRSA) is the main drug-resistant strain of *S. aureus* (Michalik et al. 2025) and the number of deaths caused by MRSA has increased the most (Naghavi et al. 2024), causing a global burden of 550,000 related deaths and 130,000 attributable deaths in 2021 (Naghavi et al. 2024). Due to the enormous global burden caused by MRSA, the World Health Organization (WHO) listed it as a high-priority pathogen in 2024 (World Health Organization. 2024b).

### Antibiotic resistance mechanisms

**Fig. 2:**
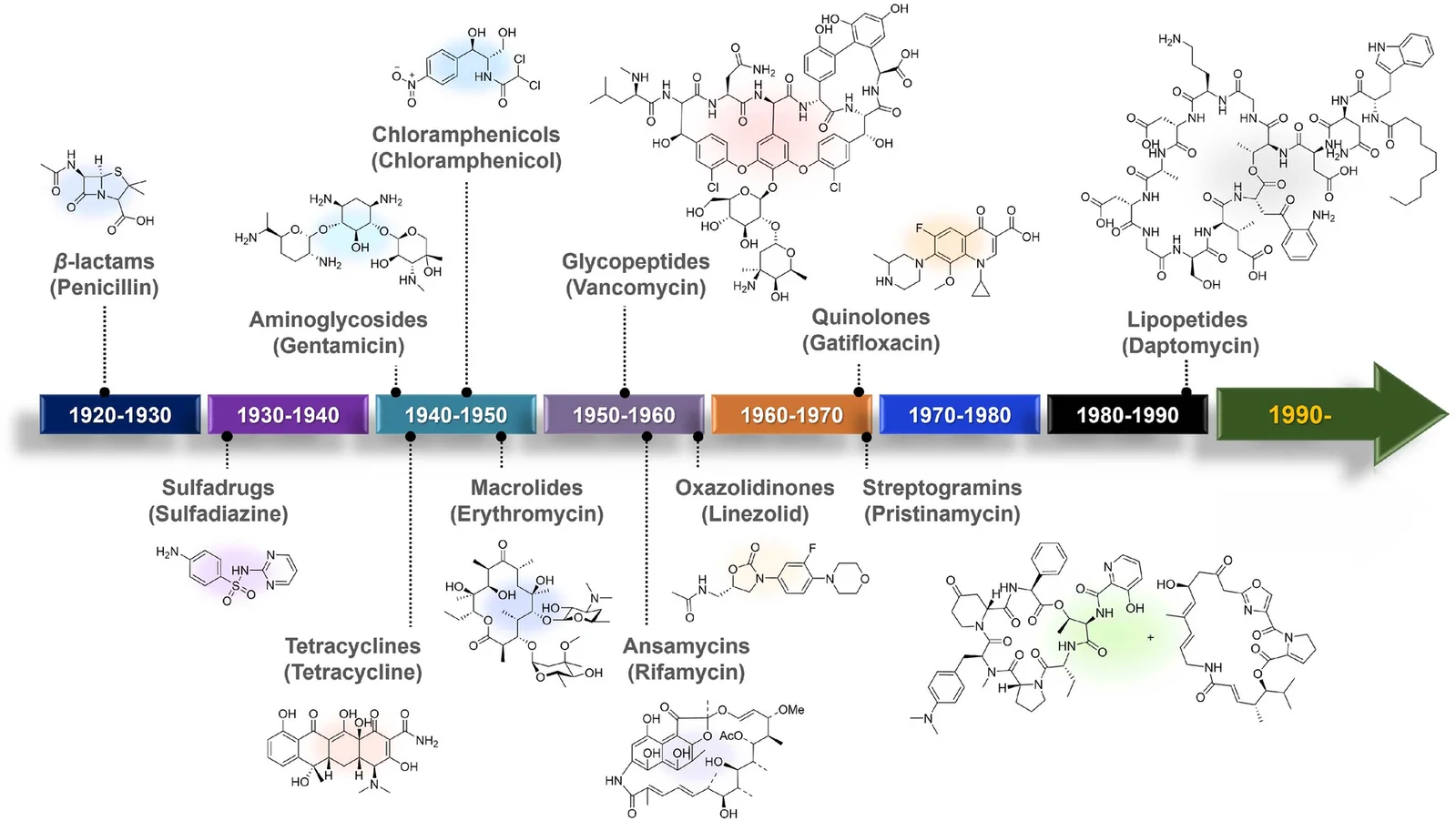
History and structures of main antibiotics (structural categories)

The types of antibiotics currently in use mainly consist of 12 varieties, including β-lactams, quinolones, aminoglycosides, and some others (Lewis 2012; Ling et al. 2015). In this endless war against drug-resistant bacteria, humans currently find themselves at a severe disadvantage, the pace of new drug development lags far behind the rate at which resistance evolves. To illustrate, only one new antibiotic class (lipopeptides) has been developed between 1964 and 2024 (Fig. 2), a speed vastly outpaced by the emergence of drug-resistant bacteria (Lewis 2012; Ling et al. 2015; Naghavi et al. 2024). For now, antibiotics like vancomycin, daptomycin, and linezolid remain humanity’s last line of defense, as resistant strains to these drugs have not yet become globally prevalent (Boswihi and Udo 2018). However, this line of defense is extremely fragile and fraught with limitations. First, none of the three drugs can reach effective intracellular concentrations without inducing toxicity (Lehar et al. 2015). Second, daptomycin cannot be used to treat pneumonia (Raja et al. 2003). And third, linezolid is not suitable for the treatment of bacteremia or infective endocarditis (Watkins and Lemonovich File^2012^). MRSA has emerged rapidly driven by antibiotic abuse and the unavoidable drawbacks of broad-spectrum antibiotics and has become a leading cause of multidrug-resistant nosocomial infections (Guo et al. 2024a, 2024b; Rosa et al. 2022).

**Fig. 3:**
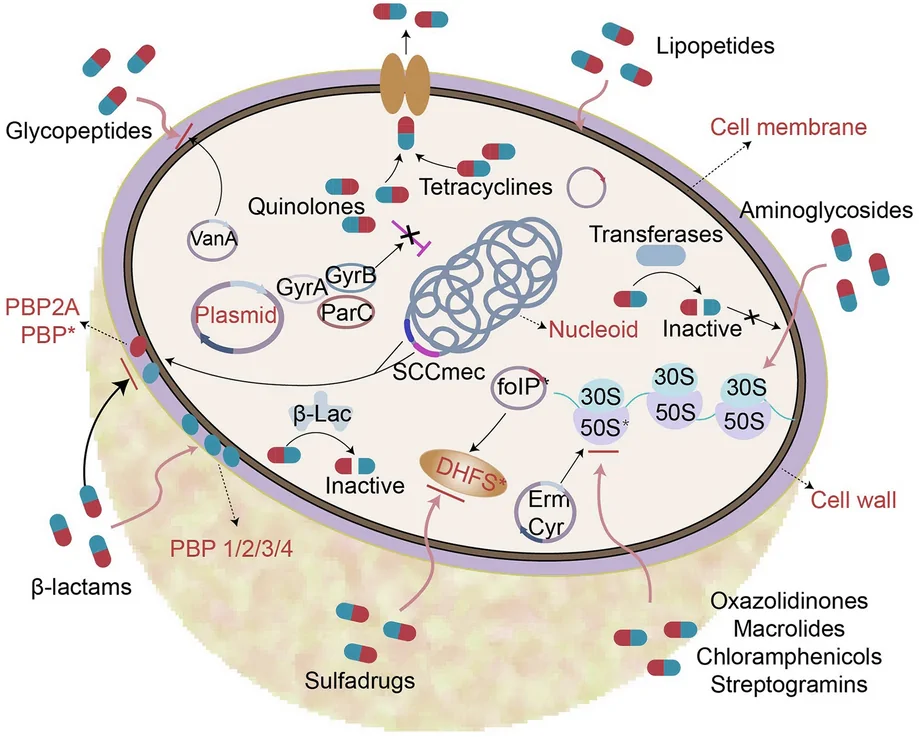
Mechanism of action and main resistance mechanisms of first-line antibiotics

The mechanisms of action of existing antibiotics are diverse, including inhibition of bacterial cell wall synthesis (Lima et al. 2020; Sarkar et al. 2017), disruption of bacterial cell membranes (Slingerland et al. 2022), suppression of nucleic acid synthesis (Pham et al. 2019), inhibition of protein synthesis (Brodersen et al. 2000; Diekema and Jones 2001; Kotra et al. 2000; Leclerc et al. 1991; Luo et al. 2024; Vázquez-Laslop and Mankin 2018; Yu and Zeng 2024), and interference with bacterial metabolic processes (Venkatesan et al. 2023). The emergence of these compounds has provided humanity with a rich arsenal against pathogenic bacteria. But with the long-term use of antibiotics and bacterial adaptability, the bacteria have evolved resistance to almost all available antibiotics. Existing research classifies the primary bacterial resistance mechanisms into five categories (Fig. 3). First, reduced intracellular drug concentrations mediated by efflux pumps, exemplified by resistance to quinolones (Ding et al. 2008; Xu et al. 2011) and tetracyclines (Schnappinger and Hillen 1996). Second, enzymatic inactivation of antibiotics through the production of modifying or degrading enzymes, such as β-lactamases that confer resistance to β-lactams (Mlynarczyk-Bonikowska et al. 2022) and aminoglycoside-modifying enzymes (Emaneini et al. 2009; Kelmani et al. 2008). Third, modification of antibiotic targets via chromosomal mutations or acquisition of plasmid-encoded resistance genes. This mechanism underlies most forms of antibiotic resistance. For instance, expression of the altered penicillin-binding protein PBP2A (Moreillon 2008; Ito et al. 2003) and mutations in PBP genes (Banerjee et al. 2010; Greninger et al. 2016; Lee et al. 2018) confer β-lactam resistance in *S. aureus*. Additionally, the VanA operon in Gram-positive cocci mediates high-level resistance to vancomycin and teicoplanin (Courvalin 2006). Fourth, bypass or alteration of metabolic pathways, such as increased synthesis of para-aminobenzoic acid by *S. aureus*, which counteracts the inhibitory effects of sulfonamides (Landy et al. 1943). Fifth, reduced drug permeability due to changes in membrane barriers, including downregulation of porin expression (Brighty et al. 1993), induction of biofilm formation regulated by cyclic dinucleotides (c-di-GMP), small non-coding RNAs (Kalia et al. 2023), or quorum sensing systems (Jenul et al. 2019; Zhao et al. 2020).

### The strategies for solving the problem of antibiotic resistance

**Fig. 4:**
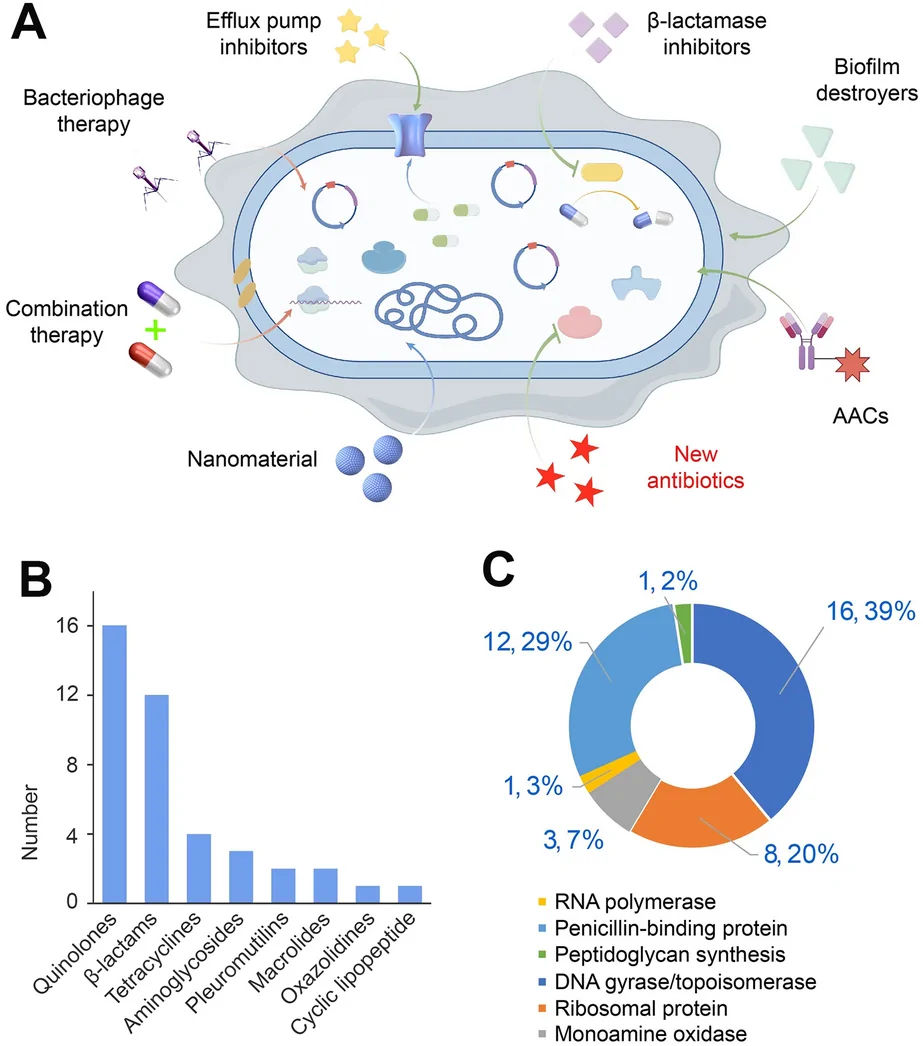
The strategies for solving the problem of antibiotic resistance. **A** Strategies against bacterial resistance (By Figdraw). **B** Structural types of antibiotics (for bacteria) launched from 2000 to 2023. **C** Antimicrobial targets of antibiotics (for bacteria) launched from 2000 to 2023

At present, in response to the development of antibiotic resistance, researchers have come up with various strategies (Fig. 4A), such as combination therapy with drugs of different mechanisms of action (Ai et al. 2023; Mantzana et al. 2023; Tamma et al. 2024) and antibiotic adjuvants combined with antimicrobial drugs (Docquier and Mangani 2018; Douafer et al. 2019; Wright 2016). To date, several antimicrobial drug combinations have entered clinical use, such as Amoxicillin/Clavulanic acid, Ceftazidime/Avibactam, and Ceftolozane/Tazobactam (Lowrence et al. 2019; van Duin et al. 2016; Zhang et al. 2024a, b, c). In addition, there are also some biofilm disruptors (Peng et al. 2025; Saigal et al. 2019), nanodevices (Panáček et al. 2024; Prasad and Gupta 2020), and bacteriophages (Huang et al. 2022; Kim et al. 2018; Segundo-Arizmendi et al. 2025; Skurnik et al. 2025) used in the development of antibacterial drugs. AB-SA01, a phage therapeutic targeting MRSA, has currently completed Phase I clinical trials (Aleksandra et al. 2020). The monoclonal antibody-antibiotic conjugate strategy has demonstrated remarkable potential in treating MRSA infections, with at least two novel conjugates, DSTA4637S and ASAK-22, proven effective in eradicating intracellular MRSA (Fan et al. 2024; Lehar et al. 2015; Yu et al. 2022).

A statistical analysis of antibiotics launched between 2000 and 2023 revealed that their main chemical structures are quinolones and β-lactams (Fig. 4B, Supplementary Table S1). Additionally, the primary mechanisms of action of these antibiotics over the last 24 years are DNA gyrase/topoisomerase inhibitors and penicillin-binding protein inhibitors, accounting for 39% and 29% of the total, respectively (Fig. 4C), (Shi et al. 2023). These data indicate that although a considerable number of antibiotics have been launched, there are no antibiotics with new chemical structures or new mechanisms of action—failing to effectively address the current situation of antibiotic resistance. Although advancements in antibiotic adjuvants and materials science can alleviate the threat posed by bacterial resistance to some extent, we should recognize that developing antibiotics with new targets and new structures is the key to solving the problem of antimicrobial resistance. In addition, the development of new antibiotics that reduce bacterial virulence and inhibit bacterial harm while maintaining symbiosis with beneficial bacteria is also a major trend in the future of antibiotics (Ford et al. 2021; Wang et al. 2025).

### The current status of MNPs in drug development

Natural products and plant mixtures accounted for 4.6% of the drugs approved by the FDA from 1981 to 2019 (Christine et al. 2024). Marine natural products (MNPs) constitute an important part of natural products. Up to September 2025, a comprehensive compound library containing 43,404 MNPs has been established. Besides, this library continues to expand rapidly, adding over 1,000 new MNPs annually (Carroll et al. 2024, 2025). According to statistics, the development of MNPs is rapid. From 3,000 marine products, 1 drug can be developed, which is approximately 1- to threefold higher than the industry average (1 in 5000–10000 tested compounds) (Gerwick and Moore 2012). MNPs play a crucial role in various fields, such as antifungal, antitumor, antibacterial activities, as well as the treatment of obesity and nonalcoholic fatty liver disease (NAFLD) (Chen et al. 2023; Du et al. 2025; Han et al. 2023; Jung et al. 2024; Liu et al. 2024; Wu et al. 2025; Zhang et al. 2025; Zou et al. 2024). Furthermore, the discovery of new natural products offers greater possibilities for the development of innovative drugs (Jung et al. 2024; Li et al. 2023, 2025; Lv et al. 2025; Xu et al.^2021^). To date, 17 drugs derived from the ocean, such as eribulin mesylate (Halavens®), trabectedin (Yondelis®), and brentuximab vedotin (Adcetris®), have been successfully launched on the market (Papon et al. 2022; Zhang et al. 2024a, b, c). Beyond marketed products, 33 marine drugs are currently in clinical development (https://www.marinepharmacology.org), which fully demonstrates that MNPs have great potential in drug development. In the field of antibacterial drug research and development, a large number of marine-derived lead molecules with significant antibacterial activity have been reported in recent years. Similarly, some reviews have systematically introduced these molecules according to pathogen categories. However, there has been no review reported on *S. aureus*.

This review systematically analyzes the mechanisms of resistance to first-line antibiotics and the bottlenecks in the development of new antibiotics. Then, we demonstrate the progress and potential of MNPs in the development of new antibiotics. We further discuss the difficulties and strategies for developing new antibiotics from marine sources, which may promote this work.

## MNPs are an important source of new antibiotics

### Overview of MNPs with antibacterial activity against *S. aureus*

**Fig. 5:**
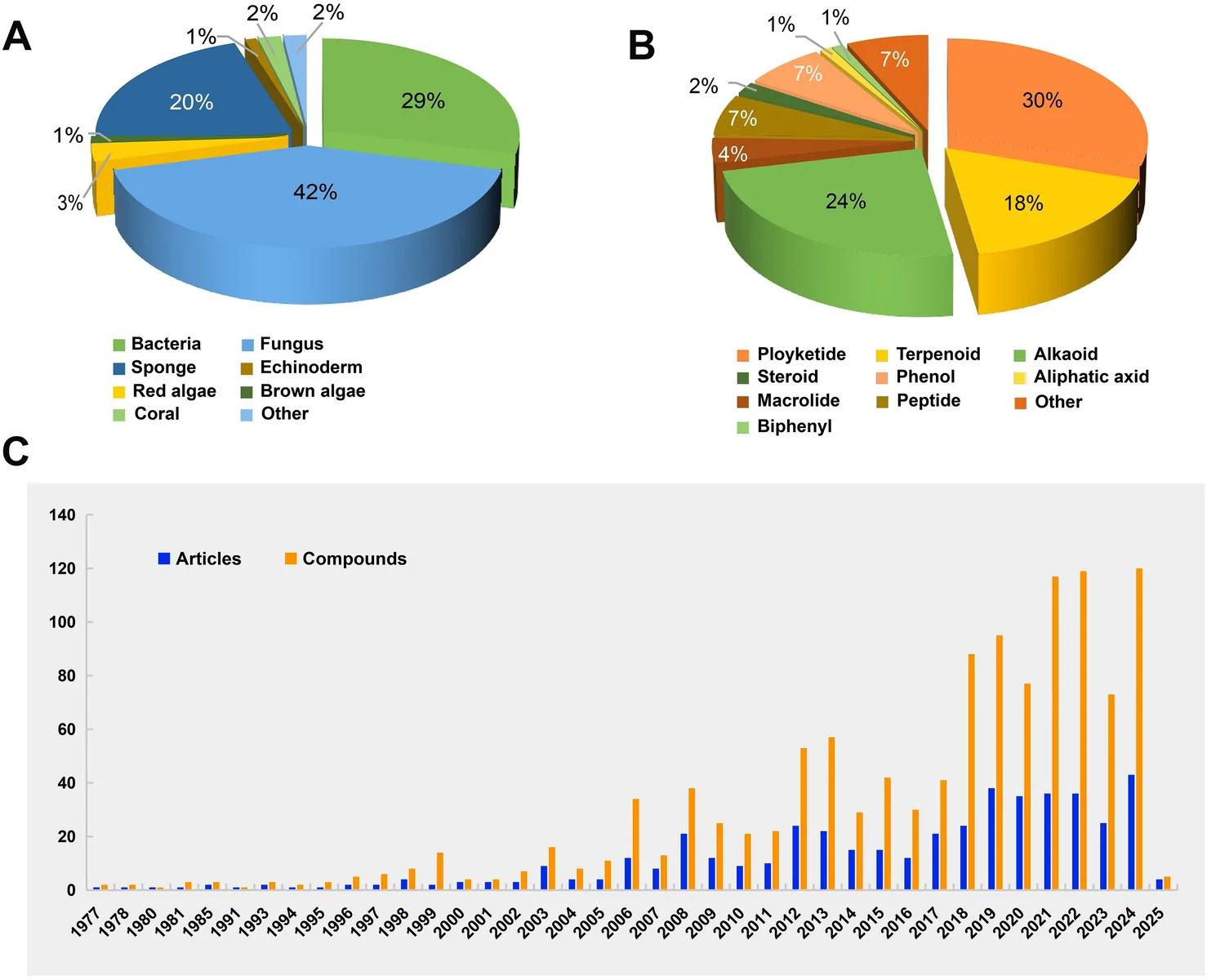
Overview of marine-derived anti-*S. aureus* compounds from 1977 to 2025. **A** Source distribution of MNPs against *S. aureus*. **B** Classification distribution of marine natural product compounds against *S. aureus*. **C** Distribution of the number of literatures and compounds on anti-*S. aureus* MNPs from 1977 to 2025

MNPs are a valuable resource for drug research and development. And many well-known drugs have been derived from MNPs. However, it remains a treasure trove that has just shown its potential but has not been thoroughly explored for the development of antibiotics. Taking *S. aureus* as an example, 1192 MNPs with anti-*S. aureus* activity have been discovered in 468 studies published between 1977 to 2025 (Fig. 5A–C, Supplementary Table S2). These MNPs can be divided into nine categories according their chemical structures, which are polyketides (30%), terpenes (18%), alkaloids (24%), macrolides (4%), peptides (7%), steroids (2%), phenols (7%), fatty acids (1%), biphenyls (1%), and others (7%). Detailed analyses of the origins of these MNPs showed that marine microorganisms, including fungi (42%) and bacteria (29%), have become the focus of antibacterial drug research. Due to the unique biological characteristics and metabolic capabilities, marine microorganisms represent a rich and largely untapped resource for the development of innovative drugs. Compared with sponges (20%), corals (2%), and echinoderms (1%), marine microorganisms can usually be well cultured in airtight fermentation tanks and bioreactors. Therefore, marine microorganisms have the advantage of being renewable, which enables them to continuously produce various pharmacologically active compounds. The above content indicates that MNPs have clearly become an important research source for the development of antibiotics against *S. aureus* infections. In addition, the number of compounds with antibacterial properties has been increasing rapidly in recent years, further demonstrating the potential of MNPs in the research and development field of anti-*S. aureus* drugs.

**Fig. 6:**
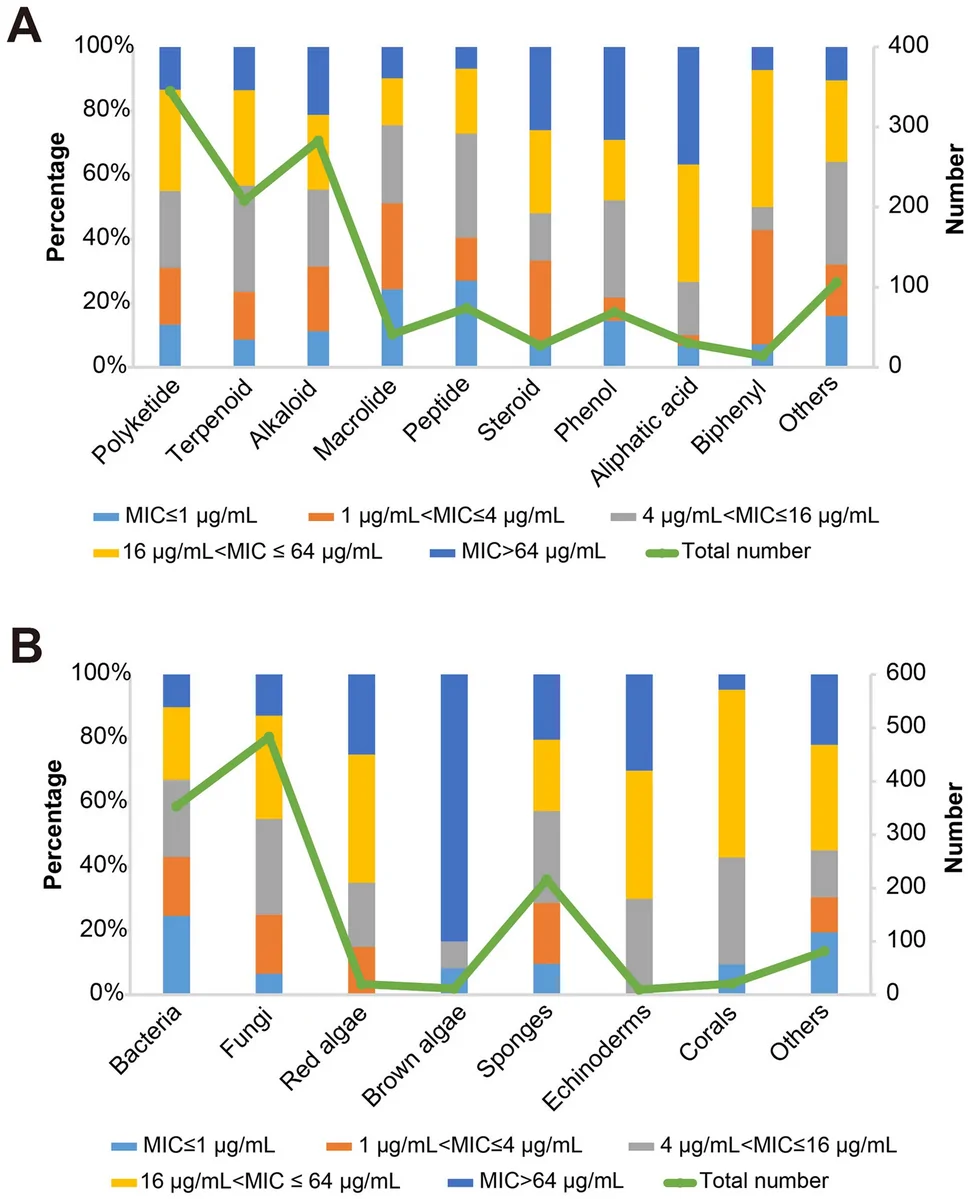
MIC distribution of marine-derived anti-*S. aureus* compounds from 1977 to 2025. **A** Percentage distribution of compounds against *S. aureus* in each chemical class for 1977–2025. **B** Percentage distribution of compounds against *S. aureus* in each origin for 1977–2025

To evaluate the antimicrobial potency distribution, we further classified these MNPs into 5 categories based on the strength of their compound activity, including minimum inhibitory concentration (MIC) ≤ 1, 1 < MIC ≤ 4, 4 < MIC ≤ 16, 16 < MIC ≤ 64, and MIC > 64 (with the unit of µg/mL) (Fig. 6A). The analysis data showed that each structural category contained compounds with significant activity (MIC ≤ 1 µg/mL), accounting for 6% to 27% of the total. Macrolides and peptides had the highest proportions, with compounds exhibiting significant activity making up 24% and 27% of the total active compounds, respectively. This indicates that they have potential as lead molecules against *S. aureus*. As shown in Fig. 6B, the analysis data indicated that compounds with significant activity were found in all sources except red algae and echinoderms, accounting for 6 to 24.6% of the total. Bacteria had the highest proportion, with compounds showing significant activity making up 24.6% of the total active compounds, suggesting that bacteria remain the primary source of potential lead molecules against *S. aureus*. Among these compounds with significant antibacterial activity derived from marine bacteria, 35.7% originate from *Streptomyces* sp. and 28.6% from *Actinomycetes* sp., which is similar to the sources of commercially available antibiotics. This fully demonstrates the important role of these two bacteria in antibiotic development. It is worth noting that *Bacillus* sp. is the third largest source of these compounds, representing a distinct origin of marine antibacterial molecules compared to existing antibiotics (Supplementary Table S3).

### Elemental, functional groups and drug-like properties analysis of MNPs with excellent anti-*S. aureus* activity

**Fig. 7:**
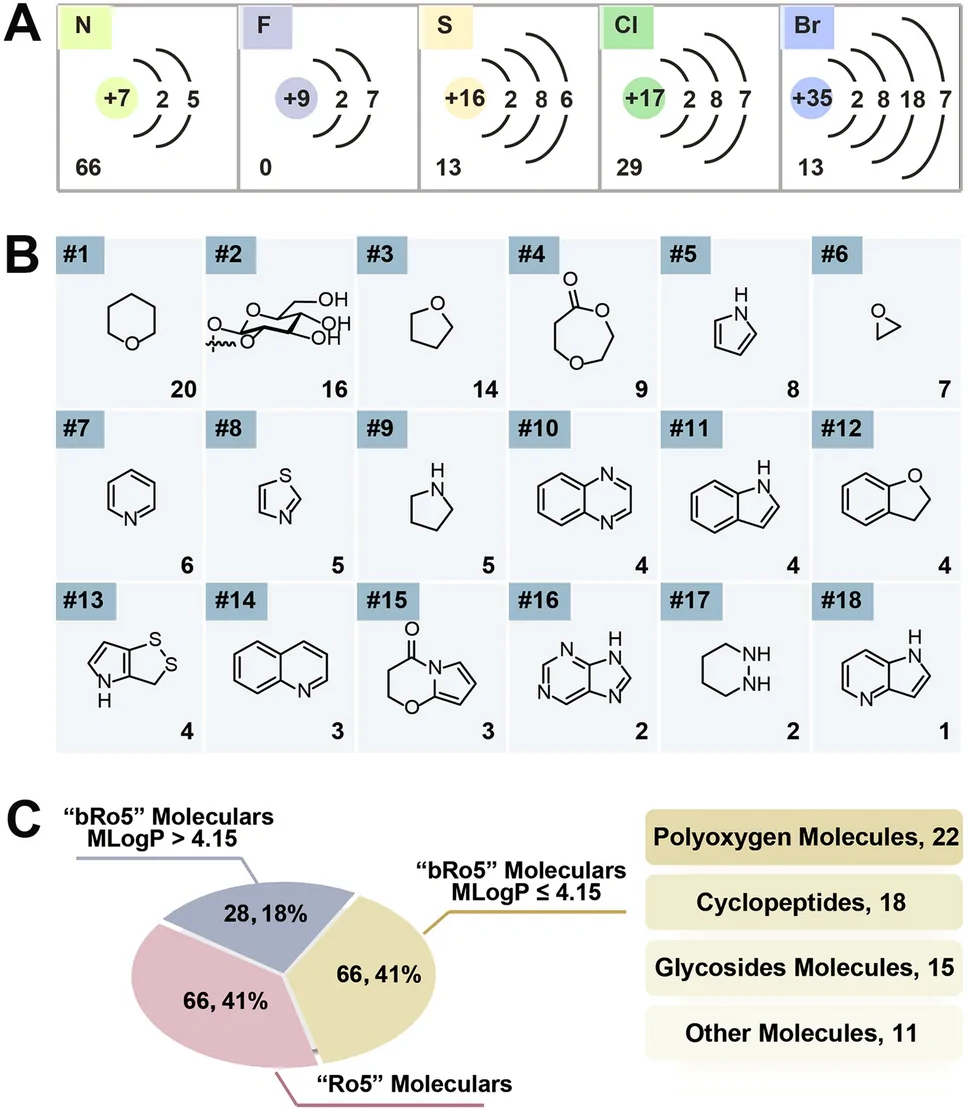
Structural analysis of 160 active molecules. **A** Distribution of special elements in anti-*S. aureus* compounds. **B** Distribution of special functional groups in anti-*S. aureus* compounds. **C** The drug-likeness prediction for the MNPs with anti-*S. aureus* activity

Owing to the distinctive environmental conditions of the ocean, bioactive compounds derived from marine organisms exhibit unique elemental compositions and structural features. In our analysis of 160 active molecules identified through screening, we found that these molecules contain key elements including nitrogen (N), sulfur (S), and halogens (e.g. chlorine [Cl] and bromine [Br]) (Fig. 7A). Specifically, the counts of molecules containing each element are as follows: 66 for N, 13 for S, 29 for Cl, and 13 for Br. This elemental distribution not only underscores the structural uniqueness of marine-derived active molecules but also reflects the specialized metabolic pathways of marine organisms. Furthermore, we analyzed the specific functional groups contained in these active molecules and found that their structures include oxygen-containing heterocycles (such as three-membered rings, tetrahydrofuran rings, tetrahydropyran rings, and seven-membered rings), glycan units, nitrogen-containing heterocycles (such as pyridine, thiazole, and pyrrole), as well as benzoheterocyclic structures (such as quinoline, purine, and piperazine) (Fig. 7B). Among them, the active molecules contain a relatively large number of tetrahydrofuran, tetrahydropyran, and glycan units, with the number of corresponding compounds being 20, 16, and 14 respectively. Unlike the structures of existing antibiotics, some active MNPs contain benzoheterocyclic structures, which demonstrate the advantage of MNPs in the discovery of structurally novel antibiotics.

To further evaluate the drug-like properties of MNPs with anti-*S. aureus* activity, molecular weight, number of hydrogen bond donors, number of hydrogen bond acceptors, and MLogP of 160 molecules (with MIC against *S. aureus* ≤ 1 µg/mL) with excellent activity were predicted by SwissADME. The results showed that among these 160 molecules, 41% fully complied with the Lipinski's Rule of Five (Ro5) (Lipinski et al. 2001), while another 41% molecules beyond of Ro5 (bRo5 molecules) had suitable lipid-water partition coefficients (Fig. 7C), which is considered the most important parameter in the drug development. These data indicate that marine natural products not only exhibit excellent activity but also possess favorable drug-like properties.

### Representative MNPs with significant anti-*S. aureus* active.

To explore the application potential of MNPs in combating *S. aureus*, this study adopted a MIC of ≤ 1 μg/mL as the core activity criterion, and conducted a systematic analysis of 242 MNPs with anti-*S. aureus* activity, focusing on investigating their activity characteristics and structure–activity relationships (SAR) (Supplementary Figs. S1–S10). It is worth noting that among these MNPs, 36 lead molecules exhibited prominent broad-spectrum anti-*S. aureus* activity—not only exerting potent inhibitory effects on methicillin-sensitive *S. aureus* (MSSA) but also demonstrating significant bacteriostatic efficacy against the MRSA, with their MIC values ≤ 1 μg/mL (Table 1). Moreover, among these 36 active compounds, most exhibit unique structural characteristics, including anthraquinone glycosides, anthraquinone dimers, complex heptacyclic systems, and dihydroindole pyrroles and some other structural types not previously seen in clinical antibiotics, highlighting the potential of marine natural products as a source for novel antibiotics (Supplementary Table S3).

**Table 1:** Representative compounds against MSSA and MRSA

Compound	MIC (µg/mL)		Compound	MIC (µg/mL)	
	MSSA	MRSA		MSSA	MRSA
Nidulin (**32**)	0.78	0.78	Berninamycin A (**145**)	0.8	0.4
Chromomycin A_9_ (**35**)	0.06–0.13	0.13	Echinomycin (**149**)	0.01	0.03
Chromomycin A_p_ (**36**)	0.06–0.25	0.06–0.25	Compound **159**	0.025	0.1
Chromomycin A_2_ (**37**)	0.03–0.06	0.06–0.25	Compound **160**	0.156	0.78
Chromomycin A_3_ (**38**)	0.06–0.13	0.13	Compound **161**	0.156	0.78
BE-43472B (**43**)	0.03–0.06	0.06–0.25	Loloatin C (**162**)	0.5	0.5
BE-43472A (**44**)	0.12–0.5	0.25–0.50	Thiocillin I (**163**)	0.05	0.78
Bisanthraquinone derivative 6 (**48**)	0.06–0.12	0.06	Thiocillin II (**164**)	0.013	0.2
Bisanthraquinone derivative 7 (**49**)	1.0	0.125–0.50	Actinomycin X2 (**165**)	0.25	0.25
Bisanthraquinone derivative 8(**50**)	0.126	0.126–0.50	2-(2′,4′-dibromophenoxy)-4,6-dibromophenol (**193**)	0.117–0.313	0.313–0.625
Citreamicin *θ* A (**51**)	1	0.25	Tianyamycin A (**217**)	0.125	0.125
Citreamicin *θ* B (**52**)	1	0.25	Tianyamycin B (**218**)	0.125	0.125
Lynamicin B (**97**)	0.8	1	Tianyamycin C (**219**)	0.125	0.125
Streptonigrin (**109**)	0.78	0.78	Tianyamycin D (**220**)	0.125	0.125
Gliotoxin (**110**)	1	1	Thiomarinol D (**231**)	< 0.01	< 0.01
Antarmycin C (**142**)	0.5	0.25–1.00	Thiomarinol E (**232**)	< 0.01	< 0.01
YM-266183 (**143**)	0.025	0.78	Thiomarinol F (**233**)	< 0.01	< 0.01
YM-266184 (**144**)	0.025	0.39	Thiomarinol G (**234**)	< 0.01	< 0.01

### Polyketide

**Fig. 8:**
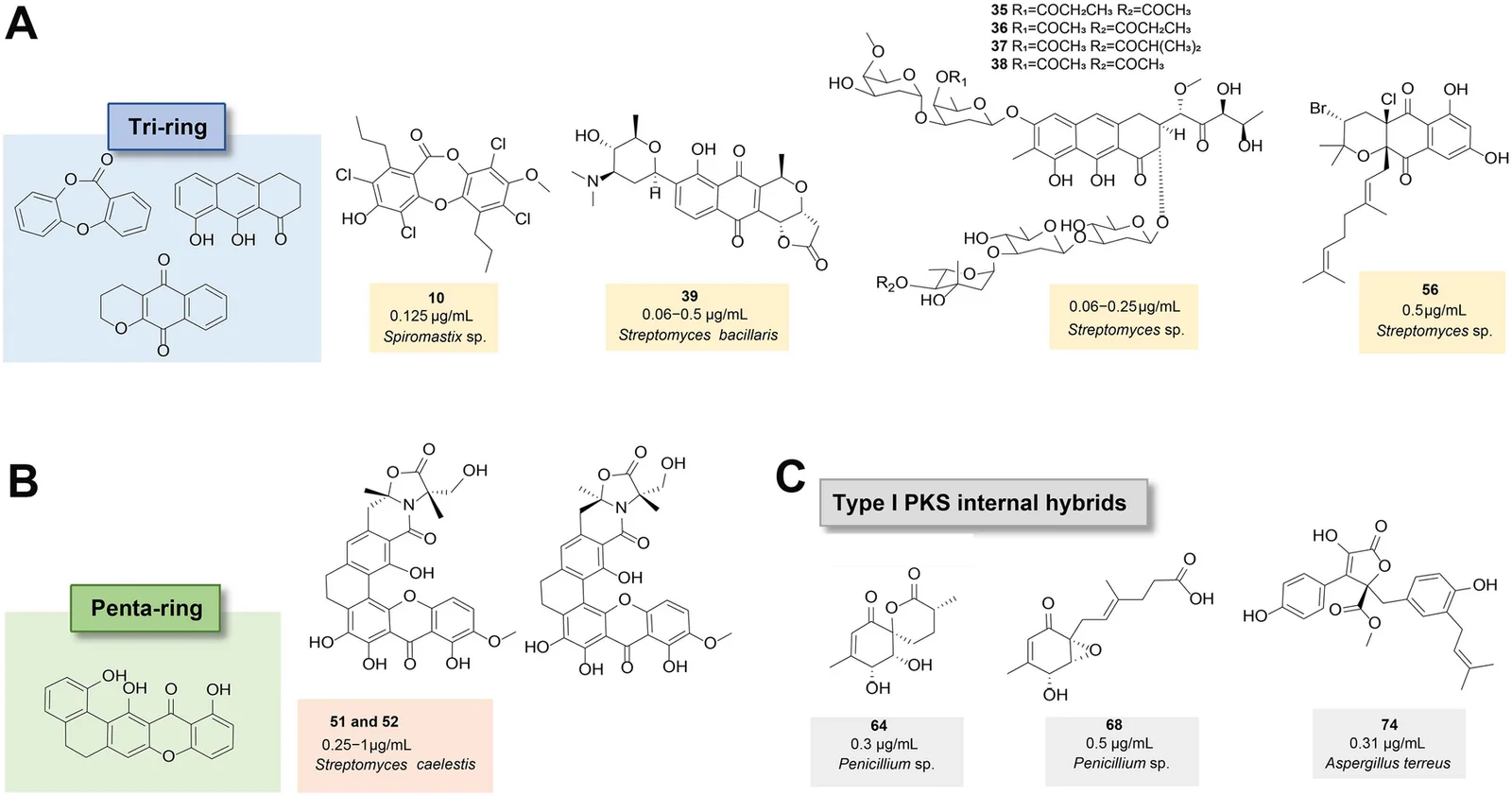
The structures of the marine polyketides with the strongest anti-*S. aureus* activity. **A** Polyketides containing a tri-ring system. **B** Polyketides containing a penta-ring system. **C** Polyketides with type I PKS internal hybrids

In a comprehensive review of the literature, the proportion of polyketides in MNPs against *S. aureus* was up to 30%. Of these, 38 polyketides displayed potent antibacterial activity against MSSA and MRSA with MIC values in the range of 0.028–1.00 µg/mL (Fig. 8A–C).

Several depsidones, including spiromastixones A–O (**1**–**15**) (Niu et al. 2014), P–S (**16**–**19**) (Niu et al. 2021), T–Z5 (**20**–**31**) (Huang et al. 2024), nidulin (**32**), 2-chlorounguinol (**33**), and unguinol (**34**) (Handayani et al. 2020) displayed potent activity against *S. aureus*. In particular, compounds **7**, **10**, **11**, **12**, and **14** have shown activity against *S. aureus* ATCC 29213 with MIC values of 0.5, 0.125, 0.5, 0.25, and 1 µg/mL. Compounds **27** and **28** exhibited inhibition activity against MRSA T144 and MRSA 1530 with MICs of 0.5–1.0 µg/mL. Structure–activity relationship (SAR) analysis indicated that halogenation plays a critical role in the antibacterial effects of depsidones. With the increase of halogen atoms, the antibacterial activity of the compound increased. The activity of chlorinated derivatives is generally stronger than that of bromine compounds.

Chromomycins were obtained from the marine-derived actinomycete *Streptomyces* sp. MBTI36, namely chromomycin A_9_ (**35**), chromomycin A_p_ (**36**), chromomycin A_2_ (**37**), and chromomycin A_3_ (**38**). Compounds **35**–**38** displayed potent activity against MSSA and MRSA with MICs of 0.06–0.25 µg/mL, which outperformed major antibiotic classes such as vancomycin and linezolid (Cho et al. 2020). It is worth noting that during a passage experiment, **35**–**38** showed low drug resistance. Investigating the anti-MRSA agents from marine actinomycetes *Streptomyces bacillaris* MBTC38 led to the discovery of lactoquinomycin A (LQM-A) (**39**) and its derivatives (**40**–**42**). These agents showed strong inhibition activities against MSSA and MRSA (MICs of 0.06–4.00 µg/mL) and weak to no inhibitory activity against Gram-negative bacteria. In particular, LQM-A (**39**) displayed the most significant antibacterial activity against MRSA (MIC = 0.25–0.50 µg/mL), with a low incidence of resistance (Chung et al. 2021).

Chemical studies of a streptomycete isolated from a cyanobacterium associated with the tropical tunicate *Ecteinascidia turbinata* led to the bioassay-guided purification of four antibacterial bisanthraquinone metabolites (**43**–**46**), which have a unique octacyclic skeleton (Socha et al. 2006). To investigate the anti-*S. aureus* of bisanthraquinone, four semi-synthetic derivatives (**47**–**50**) were prepared (Socha et al. 2006). The MICs and minimum bactericidal concentration (MBC) of compounds **43**–**50** have been evaluated. A wide range of activities were observed for **43**–**50** (MIC = 0.0275–32.0 µg/mL), suggesting that the C-1′ methoxyl substituent is not conducive to the activity of the compound (Socha et al. 2006).

Citreamicins *θ* A and B (**51** and **52**), a pair of diastereomers that possess a 5/6/6/6/6/6/6 seven-ring system, citreaglycon A (**53**) and dehydrocitreaglycon A (**54**) obtained from marine-derived *Streptomyces caelestis* have shown inhibition activities against MSSA and MRSA with similar MIC values (0.25–16.0 µg/mL). The reduced antibacterial activity of **53** and **54** (MICs of 8.0–16.0 µg/mL compared to **51** and **52** with MICs of 0.25–1.00 µg/mL) suggested that the five-member nitrogen heterocycle is the key to activity (Liu et al. 2012). Napyradiomycins (**55**–**63**) were isolated from the marine-derived *Streptomyces* sp. SCSIO 10428. Compounds **58**–**62** exhibited similar anti-*S. aureus* ATCC 29213 activities (MICs of 0.5–1.0 µg/mL) (Wu et al. 2013a, b).

Five novel compounds have new carboxylic acid side chains, isolated from the deep-sea-derived fungus *Penicillium* sp. F23-2, namely, penicyclones A–E (**64**–**68**) (Guo et al. 2015), possess strong activity against *S. aureus* with MICs of 0.3–1.0 µg/mL. In addition to these MNPs, a further series of metabolites **70–95** were reported from fungi (Chen et al. 2021, 2013; Cueto et al. 2001; Song et al. 2022; Wu et al. 2016; Zhang et al. 2012a, b; Zhang et al. 2022; Zhang et al. 2024a, b, c), actinomycetes (Kim et al. 2022; Kokkini et al. 2023), and other bacteria (Jiang et al. 2018; Kim et al. 2022; Ryu et al. 2021) and displayed potent antibacterial activity.

### Alkaloid

**Fig. 9:**
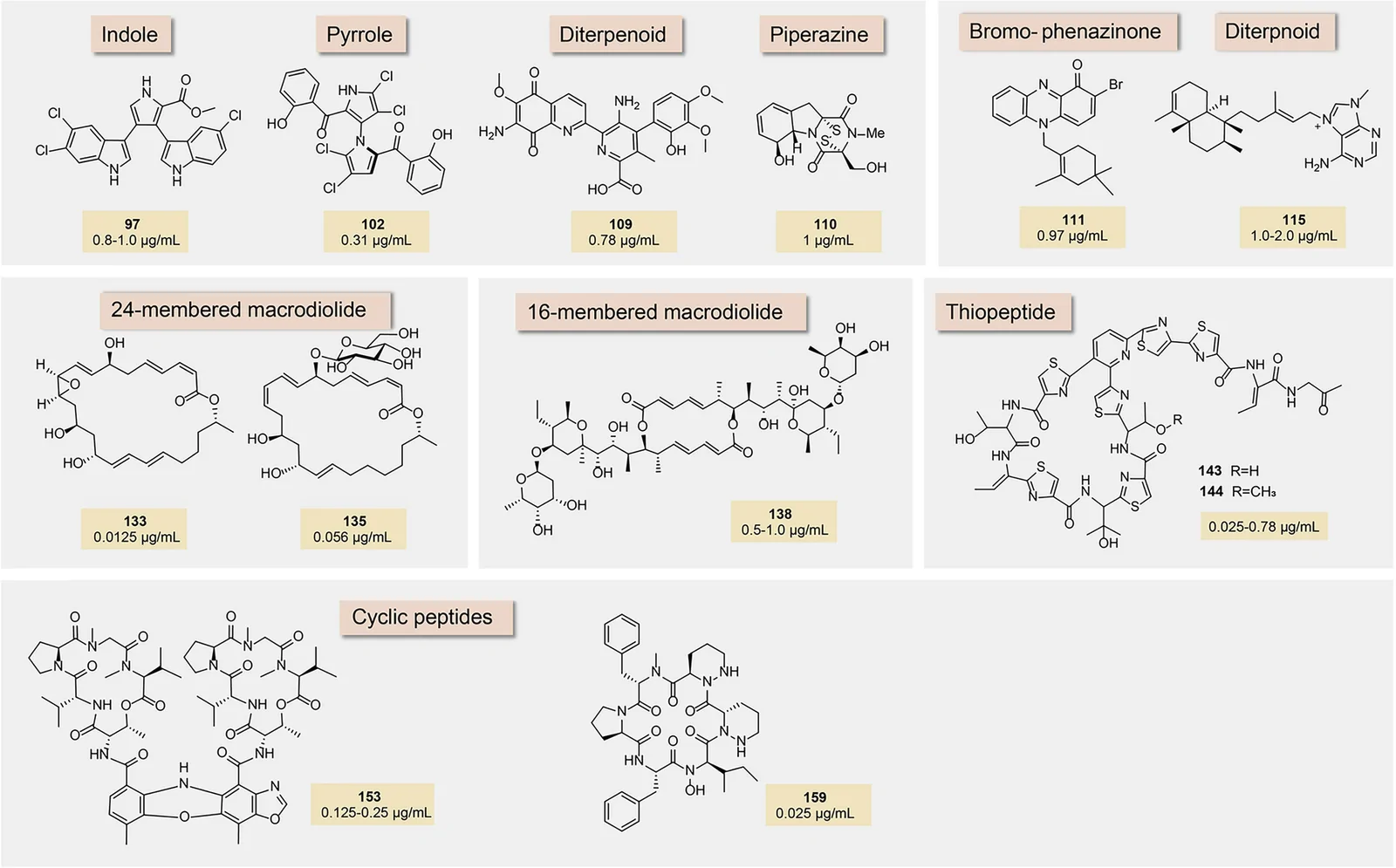
The structures of marine alkaloids with the strongest activity

In the process of investigating MNPs with anti-*S. aureus* alkaloids, we found that alkaloids accounted for 24%, second only to polykeides (Fig. 9). Due to the unique structure and biological activity of alkaloids, these compounds have become important resources for drug research and development. Chlorinated *bis*-indole alkaloids, lynamicins A–E (**96**–**100**) (McArthur et al. 2008), and 13-oxofumitremorgin B (**101**) (Zhang et al. 2022) showed potent activity against *S. aureus*. Compounds **96**–**100** were obtained from marine actinomycete NPS12745, displayed antibacterial activity against MSSA and MRSA with MIC values ranging from 0.8–20.0 µg/mL. An SAR analysis suggested that halogen substitution may be the key to improve the activity of the compound (McArthur et al. 2008). Compound **101** has shown inhibition activity against MRSA (MIC = 0.59 µg/mL) and was isolated from the marine-derived *Aspergillus fumigatus* (Zhang et al. 2022). Marinopyrroles A–F (**102**–**107**) have been discovered from the actinomycete *Streptomyces* sp. CNQ-418. Compounds **102**–**104** showed strong activity against MRSA with MIC values of 0.31, 0.63, and 0.16 µg/mL. At the same time, SAR showed that alicylyl hydroxyl and N–H were crucial for anti-MRSA activity (Nenkep et al. 2010).

Deoxynyboquinone (DNQ, **108**), discovered from the actinomycete *Pseudonocardia* sp. SCSIO 01299, displayed strong activity against *S. aureus* ATCC 29213 with a MIC value of 1 µg/mL (Li et al. 2011). Streptonigrin (**109**), isolated from a deep-sea-derived *Streptomycetes* sp. SMS636, exhibited significant inhibition activity against MRSA at the same concentration (MIC = 0.78 µg/mL) (Xu et al. 2019). A diketopiperazine compound, gliotoxin (**110**), isolated from a marine-derived fungus of the genus *Pseudallescheria*, exhibited potent antibacterial activity against MRSA with an MIC value of 1 µg/mL (Li et al. 2006). Bioassay-guided fractionation led to marinocyanin A (**111**) and WS-9659A (**112**) from two marine actinomycetes, CNY-960 and CNS-284. Compounds **111** and **112** both exhibited an MIC value of 0.97 µg/mL against *S. aureus* (Asolkar et al. 2017). Other alkaloid compounds **113**–**124** have also been reported to have significant anti-*S. aureus* activities (Chen et al. 2022; Hong et al. 2017; Hughes et al. 2010; Song et al. 2020; Wang et al. 2010; Zha et al. 2024; Zhang et al. 2022).

### Macrolide

A series of 24-membered lactones, macrolactin M (**125**) (Nagao et al. 1997), macrolactins O–R (**126**–**129**) (Zheng et al. 2007), macrolactins A and B (**130** and **131**), macrolactin S (**132**) (Lu et al. 2008), and gageomacrolactins (**133**–**135**) (Tareq et al. 2013) were isolated from *Bacillus* sp. obtained from soil sample (Zheng et al. 2007), sea sediment (Lu et al. 2008), and sediment sample (Tareq et al. 2013). Structurally, compounds **126**–**131**,** 134,** and** 135** are glycosylated. Biological assays against *S. aureus* showed that **132**–**135** possess strong activity with MICs of 0.7, 0.0125, 0.063, and 0.056 µg/mL (Lu et al. 2008; Tareq et al. 2013). It is worth mentioning that compounds **133**–**135** showed no cytotoxicity on multiple cancer cell lines (GI_50_ > 30 µg/mL) (Tareq et al. 2013). And compound **128** inhibited *S. aureus* peptide deformylase (PDF) in dose-dependent manners with median inhibition concentration (IC_50_) values of 12.1 µM (Zheng et al. 2007).

To investigate the glycosidic antibiotics from the marine sediment derived strain *Streptomyces* sp. 7–145 (Wu et al. 2013a, b) and deep-sea-derived *Pseudonocardia antarctica* (Zhou et al. 2025), led to the discovery of 11′,12′-dehydroelaiophylin (**136**), 11,11′-*O*-dimethyl-14′-deethyl-14′-methylelaiophylin (**137**), elaiophylin (**138**), 11-*O*-methylelaiophylin (**139**), 11,11′-*O*-dimethylelaiophylin (**140**), andefomycin G (**141**) (Wu et al. 2013a, b), and antarmycin C (**142**) (Zhou et al. 2025). Antimicrobial bioassay results indicated that **136**, **138**, **139**, and **141** have inhibitory activity against multiple strains of *S. aureus* (MICs = 0.5–2.0 µg/mL) (Wu et al. 2013a, b). Compound **142** containing a symmetric 16-membered macrodiolide core with two pendant vancosamine moieties, one of which is glycosylated, exhibited potent activity against MSSA and MRSA with MICs of 0.25–1.00 µg/mL (Zhou et al. 2025) (Fig. 9).

### Peptide

A series of thiopeptide antibiotics with significant antibacterial activity was discovered from marine microorganisms (Fig. 9). Two novel thiopeptides, YM-266183 (**143**) and YM-266184 (**144**), exhibited significant antibacterial activity against *S. aureus* FDA 209P and *S. aureus* CAY 27701 with MICs of 0.025–0.780 µg/mL, obtained from *Bacillus cereus* QN03323, which was isolated from the marine sponge *Halichondria japonica* (Nagai et al. 2003). Berninamycins A, B, J, and K (**145**–**148**) were obtained from marine-derived *Streptomyces* sp. and showed inhibition activity against *S. aureus* with MICs of 0.4–64.0 µg/mL (De et al. 2021).

To investigate against drug-resistant and biofilm-forming *S. aureus*, new echinomycin antibiotics, echinomycin (**149**), FD-991 (**150**), depsiechinoserine (**151**), and echinoserine (**152**), were isolated from marine streptomycete *Streptomyces* sp. **149** and **150** exhibited potent inhibition activities against two clinical isolates (MRSA-L32 and MRSA-L44) and MSSA with MICs of 0.01–2.00 µg/mL. Compounds** 151** and** 152** also showed significant bactericidal effect with MBCs of 0.45–7.17 µg/mL. Further activity studies showed that **149** exhibited inhibitory effects on MSSA biofilm formation, with a minimum biofilm inhibitory concentration (MBIC) of 0.03 µg/mL and a minimal biofilm eradication concentration (MBEC) of 0.22 µg/mL (Socha et al. 2009).

Actinomycins D_1_–D_4_ (**153**–**156**) and actinomycin D (**157**) were obtained from a marine sponge-associated *Streptomyces* sp. LHW52447. In particular, compounds **153** and **154** were two novel D-type actinomycin analogues with the incorporation of an oxazole unit into the central phenoxazinone chromophore. Compared with compounds **155**–**157** (MIC = 0.5–1.0 µg/mL), **153** and **154** exhibited stronger activities against MRSA (MIC = 0.125–0.250 µg/mL), indicating that the additional azole unit was critical for enhanced anti-MRSA activity (Jiao et al. 2018). Four cyclic peptides (**158**–**161**) were isolated from the marine *Streptomyces* sp. and displayed potent activities against *S. aureus* ATCC 6538 with MIC values of 1.25, 0.025, 0.156, and 0.156 µg/mL, respectively. In addition, **159**–**161** showed inhibitory effects against MRSA with MIC values of 0.1, 0.78, and 0.78 µg/mL (Jiang et al. 2021). Other peptide compounds **162**–**165** have also been reported to have significant anti-*S. aureus* activities (Gerard et al. 1999; Nagai et al. 2003).

### Terpene

Cembrane diterpenoids are a class of isoprenoid and consist of a fourteen-membered carbocyclic ring with an isopropyl residue at C-1 and three methyl groups at C-4, C-8, and C-12, which displayed antibacterial, anticancer, anti-inflammatory, and other biological activities (Nurrachma et al. 2021). Sarcophytolide (**166**), obtained from soft coral *Sarcophyton glaucum*, exhibited potent activity against *S. aureus* with an MIC of 0.19 µg/mL (Badria et al. 1998). Chemical investigation of soft coral *Sarcophyton trocheliophorum* led to the discovery of 1,13-di-epi-13-acetoxy launine P (**167**), 13-oxo-thunbergol (**168**), isocrassumol B (**169**), 7α, 8α-sarcophine (**170**), launine P (**171**), and sarcophytonin B (**172**). Compound **172** displayed anti-*S. aureus* activity with MIC value less than 0.5 µg/mL (Ren et al. 2023).

**Fig. 10:**
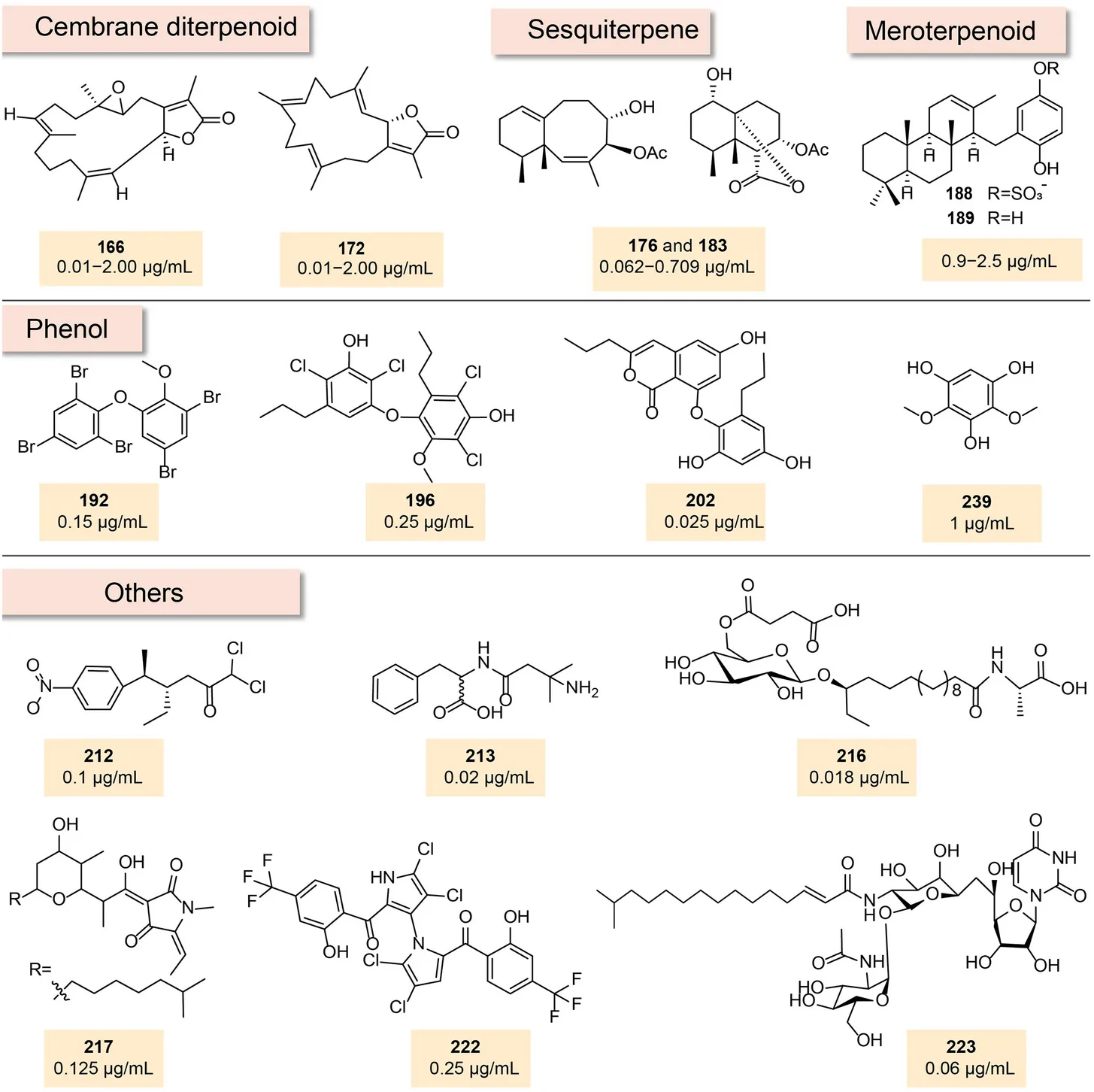
The structures of the phenol and other compounds with the strongest activity

Meroindenon (**173**) and merochlorins E (**174**) and F (**175**) possess a tetrahydroxynaphthalene core and a C_15_-isoprene unit and were obtained from the marine-derived bacterium *Streptomyces* sp. Compared with **173** (MIC = 128 µg/mL), the significant anti-*S.aureus* ATCC 6538 activities of **174** and **175** (MIC = 2 and 1 µg/mL) indicated that the gem-dimethylmethylcyclohexene ring plays an important role in the antibacterial activity (Ryu et al. 2019). A series of sesquiterpenes, paralemnolins D, E, J, P, X, Y (**176**–**181**), 2-deoxy-12-oxolemnacarnol (**182**), paralemnolide A (**183**), parathyrsoidin G (**184**), 6α-acetyl-1(10)-α-13-nornardosin-7-one (**185**), 6α-acetyl-7α-acetate-1(10)-α-13-nornardosine (**186**), and clavukoellian F (**187**), obtained from soft coral *Paralemnalia thyrsoides*, showed inhibition activities against *S. aureus* with MICs of 0.062 – 0.709 µg/mL. Among them, compound **183** significantly inhibited bacterial growth (MIC = 0.062 µg/mL), while compound **182** did not show antibacterial activity (Elshamy et al. 2021). Fascioquinols A (**188**) and B (**189**) exhibited strong activities against *S. aureus* (IC_50_ = 0.9–2.5 µmol/L) and were obtained from marine sponge *Fasciospongia* sp. (Zhang et al. 2011) (Fig. 10).

### Phenol

It is well known that marine organisms can produce phenolic metabolites (6%), which have significant antibacterial activity. A series of spiromastols, polybrominated diphenyl esters X–XII (**190**–**192**), 2-(2′,4′-dibromophenoxy)-4,6-dibromophenol (**193**), and spiromastols A–K (**194**–**204**) were obtained from marine sponges (Popov et al. 1999; Shridhar et al. 2009) and deep-sea-derived fungus (Niu et al. 2015) (Fig. 10). Biological activity results showed compounds **190**–**192** displayed strong activity against *S. aureus* with MICs of 0.36, 0.36, and 0.15 µg/mL (Popov et al. 1999). In particular, compound **193** showed significant inhibitory activity against multiple strains of *S. aureus* (20 clinically derived MRSA strains, 10 standard MRSA strains procured from culture banks, and two standard MSSA strains procured from culture banks) with MIC values ranging from 0.117 – 10.0 µg/mL (Shridhar et al. 2009). Compounds **194**–**204**, with a rare *n*-propyl group, were isolated from the marine fungus *Spiromastix* sp. MCCC 3A00308. Compounds **194**–**196** showed potent activity against *S. aureus* ATCC 25923 with MIC values of 0.5, 4.0, and 0.25 µg/mL (chloroamphenicol, 1 µg/mL), while compounds **202**–**204** exhibited moderate activity (MIC = 8, 32, 32 µg/mL). The SAR analysis of **194**–**204** showed that the antibacterial activity depended on the substitution of A and B rings (Niu et al. 2015). Another type of phenol, 2,4-diacetylphloroglucinol (DAPG) (**205**), isolated from a marine alga-derived *Pseudomonas* sp. AMSN, exhibited potent activity against MRSA at 1 µg/mL (Isnansetyo et al. 2003; Kamei and Isnansetyo 2003). Other phenol compounds **206**–**210** have also been reported to have significant anti-*S. aureus* activities (Ki et al. 2019; Popov et al. 1999).

### Others

Chemical investigation of the marine actinomycete *Nocardia* sp. ALAA 2000, obtained from red alga *Laurenica spectabilis*, led to the isolation of justicidin B (**211**) and 1,1-dichloro-4-ethyl-5-(4-nitro-phenyl)-hexan-2-one (**212**). Compound **212** is unique in containing both chlorination and a rarely observed nitro group. Compounds **211** and **212** displayed potent activities against *S. aureus* ATCC 6538 with MICs of 1 and 0.1 µg/mL (El-Gendy et al. 2008). Phenamide (**213**), which was isolated from amphipod-derived *Aspergillus sydowii* MBC15-11F, showed anti-*S. aureus* activity with an MIC value of 0.02 μg/mL (Qader et al. 2023).

A potent anti-*S. aureus* ATCC43300 compound alterporriol A (**214**), with an MIC of 0.5 µg/mL, was isolated from marine-derived fungus *Pleospora* sp. (Liu et al. 2017). Ieodoglucomide C (**215**) and ieodoglycolipid (**216**), from marine-derived bacterium *Bacillus licheniformis*, exhibited inhibition activities against *S. aureus* at 0.018 and 0.019 µg/mL (Tareq et al. 2015). Tianyamycins A–D (**217**–**220**), which were discovered from a marine-derived *Streptomyces* sp. LDBS7850, exhibited inhibition activities against MSSA and MRSA at the same concentration (MIC = 0.125 μg/mL) (Han et al. 2021). Marinopyrrole A (MA) (**221**), with a rare bipyrrole chemical skeleton, obtained from marine-derived *Streptomyces* sp., displayed potent activity against MRSA with an MIC of 0.75 μg/mL and MBC of 1.05 μg/mL. MA-D1 (**222**) was the best MA derivative, showing more potent anti-MRSA activity with an MIC of 0.25 μg/mL and an MBC of 0.45 μg/mL (Guo et al. 2024).

Investigation of marine-derived actinomycete *Streptomyces* sp. MBTG32 led to the isolation of tunicamycin VII (**223**), tunicamycin VIII (**224**), corynetoxin U17a (**225**), and tunicamycin IX (**226**). Compounds **223**–**226** showed strong activities against *S. aureus* ATCC6538p with MIC values of 0.06–0.25 µg/mL. Further mechanism studies indicated that **223**–**226** displayed potent inhibitory activities on *S. aureus* MurNAc-pentapeptide translocase (MraY), with IC_50_ values of 0.08–0.21 µg/mL (Lee et al. 2024a, b). Other compounds **227**–**242** have also been reported to have significant anti-*S. aureus* activities (Carballeira et al. 1998; Chen et al. 2013; Cheng et al. 2021; Hadjkacem et al. 2024; Jiang et al. 2018; Needham et al. 1994; Quévrain et al. 2009; Shang et al. 2012; Shiozawa et al. 1997; Wang et al. 2022; Zhang et al. 2022) (Fig. 10).

## Opportunities of MNPs in the drug development against *S. aureus*

### The next line of defense against *S. aureus* infection

The ocean with rich biological resources and natural products is an important new resource for antibiotic research and development in the future. There are about 1,192 MNPs with anti-*S. aureus* activity and more than 70% of them derive from marine microorganisms. More than half of these compounds have an activity against MRSA and about 80 compounds against one or more MRSA strains with an MIC ≤ 1 µg/mL. Some of these can reach the level of “ng/mL.” For example, the MICs of echinomycin and thiocillin Ⅱ against MRSA are 0.033 µg/ml and 0.037 µg/mL, respectively (Cueto et al. 2001; Socha et al. 2009).

### MNPs as a reserve resource for novel antibiotics

The key to solve the problem of antibiotic resistance is to develop new antibiotics for new targets, especially those that do not readily develop antibiotic resistance. As an important resource for the development of anti-*S. aureus* drugs in the future, the potential of MNPs in developing new target drugs has been demonstrated already. There are several compounds with new mechanisms that have not been discovered. For example, halenaquinol is an SrtA inhibitor that can block the anchoring of bacterial SpA on the cell wall surface (Lee et al. 2024a, b). Abyssomicin C is another example, and it is an inhibitor for the para-aminobenzoic acid synthesis of *S. aureus* (Nicolaou et al. 2008). In addition, the structures of most MNPs are relatively novel and differ from existing types of antibiotics. According to the lock-and-key principle of protein–ligand recognition, novel structures often bring unlimited possibilities for the discovery of new targets.

### Narrow spectrum antibiotics are beneficial for alleviating drug resistance

Conventional pathogen and drug resistance diagnostics, which took hours or days to gain results in the past, made a need to initiate broad-spectrum antibiotic treatment before the offending microbe is defined (Wollein Waldetoft and Brown 2022). However, the extensive use of broad-spectrum antibiotics has promoted the generation of drug resistance and the destruction of human normal flora, which causes more serious harm to humans (Böttcher and Gersbach 2022; Diamantis et al. 2022). With the development of rapid diagnostics for pathogen and drug resistance, narrow-spectrum antibiotics that have the advantages of fewer side effects and less likelihood of inducing drug resistance are gaining more attention and becoming an important direction in antibiotic research and development. Among the rich active MNPs, there are some compounds with the potential to develop narrow-spectrum antibiotics. For example, both tunicamycin VIII (Lee et al. 2024a, b) and resistomycin (Kim et al. 2022) only act on Gram-positive bacteria and have a weak effect on Gram-negative bacteria.

## Challenges and strategies of MNPs in the drug development against *S. aureus*

### Effective methods for discovering active MNPs

MNPs are a vast compound library containing 43,404 compounds. However, due to the limitations of research teams in terms of technology or funding, a large amount of analysis has not yet been conducted to test for anti-*S. aureus* activity, resulting in only about 2% of the MNPs being identified as anti-*S. aureus* molecules. High-throughput screening technology is characterized by its speed and scalability, allowing for the rapid completion of large-scale compound screening and the identification of drug lead compounds in a short period. It has been widely used in the development of various drugs, including antibiotics (Chiaraviglio and Kirby 2015; Sun et al. 2019) and antitumor agents (Glass et al. 2008; Talaulikar et al. 2023). Virtual screening technology is one of the main strategies for drug lead compound discovery and has been widely applied in the early stages of drug research (Chen et al. 2024; Li et al. 2024; Tripathi and Bandyopadhyay 2022; Yan et al. 2020). The discovery of new antibacterial targets such as aminoacyl-tRNA synthetase (Pang et al. 2021), lipid II, and lipid III (Ling et al. 2015) has injected new vitality into the development of novel antibacterial drugs. Furthermore, while striving for speed, the discovery and isolation of MNPs guided by activity or based on chemical ecology is also an important strategy. The combination of these strategies will fully develop the potential of MNPs in the discovery of anti-MRSA lead molecules, providing a more abundant material basis for the development of anti-MRSA drugs.

### Identification of compound targets of MNPs.

With the in-depth study of natural products, the research on their targets have gradually attracted the attention of researchers. More and more research technologies, such as various omics technologies, target fishing, and some other technologies have been applied to the study of the mechanisms and targets of natural products. Among these, probe-free chemoproteomics, a novel method for target identification that requires no modification of active molecules, plays a key role in identifying the targets of active natural products. Based on this method, direct targets of more and more natural products, either terrestrial natural products or MNPs, have been revealed. Examples include shizukaol A (Tang et al. 2021), vioprolide A (Kirsch et al. 2019), and so on.

### Addressing the pharmaceutical source issue of active MNPs

MNPs with significant activity are the most important lead compounds in drug development. However, most natural products have problems such as low natural content and difficult separation, which seriously hinder further research. Chemical total synthesis is the most common and effective method in the large-scale preparation of active molecules. Therefore, the total synthesis of such molecules has achieved remarkable results. One noted example is the total synthesis of abyssomicin C, a marine anti- *S. aureus* molecule with a novel target. Since its discovery in 2004, at least two total syntheses (Nicolaou and Harrison 2006a, 2006b; Zapf et al. 2005) and two formal synthetic studies (Couladouros et al. 2006; Vidali et al. 2020) of abyssomicin C have been reported (Bihelovic and Saicic 2012; Nicolaou and Harrison 2006a, 2006b). Biosynthesis is another effective method to prepare the active molecules on a large scale. A notable example of this strategy is the industrial synthesis of trabectedin.

### The application prospects of artificial intelligence in the research of MNPs

Artificial intelligence (AI) is widely applied in various scientific research fields due to its ability to absorb, recognize, and process complex information. In recent years, AI methods including machine learning (ML) and deep learning (DL) have gradually been integrated into natural product discovery and drug development (Liu et al. 2023; Mullowney et al. 2023; Saldívar-González et al. 2022). Artificial intelligence can not only accelerate the discovery of new natural products through in-depth exploration of biosynthetic gene clusters but also rapidly complete the identification of novel compounds by accurately predicting mass spectrometry (MS) and nuclear magnetic resonance (NMR) data (Burns et al. 2019; Dührkop et al. 2019; Kloosterman et al. 2020; Merwin et al. 2020; Reher et al. 2020). More importantly, AI holds great promise in predicting macromolecular targets, related bioactivities, and potential toxicity of natural products, providing a powerful impetus for the development of natural products into drugs. Integrating artificial intelligence into the development of marine-derived anti-MRSA lead molecules will accelerate progress across multiple dimensions, including compound sourcing, rapid activity discovery, and mechanism of action (Conde et al. 2021; Mullowney et al. 2023; Reker et al. 2020).

### Policy guidance and incentive programs are necessary

Compared with other types of drugs, antibiotics have a relatively low return on investment, so large pharmaceutical companies have given up investing in this field (Plackett 2020). In addition, from the analysis of antibiotics approved since 2000, the structural modification or combination of existing drugs, which can greatly reduce the costs for antibiotic research, has become the direction pursued by pharmaceutical companies. Due to the above factors, the attention and investment in MNPs are far from enough to support their rapid development.

**Fig. 11:**
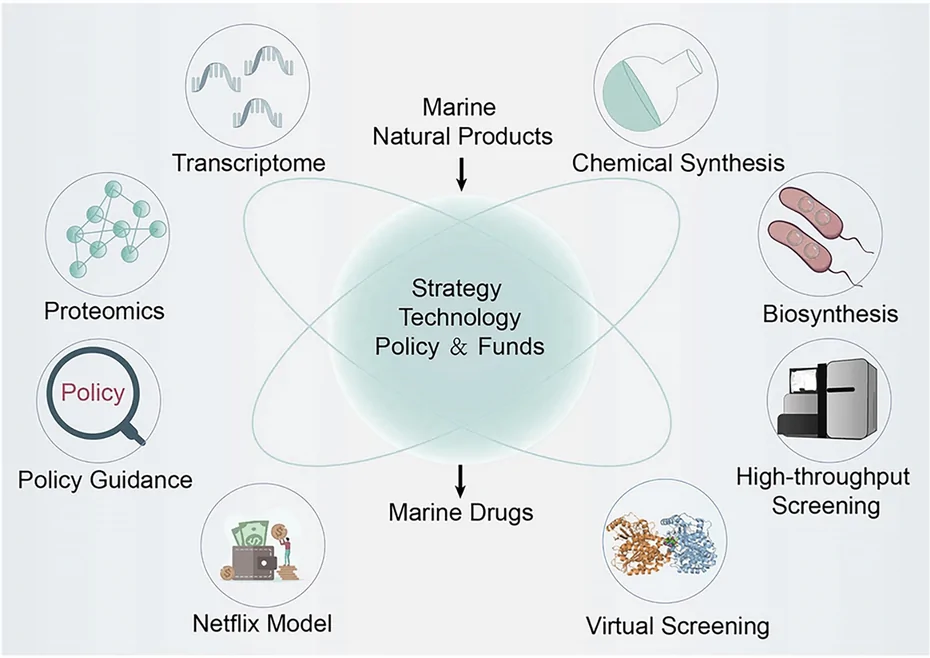
Factors required for the development of antibiotics from marine sources

The increasingly serious problem of bacterial resistance forces government policy makers to set up more strategies, such as tax preferences or subsidies, to attract funding and other resources for antimicrobial drug discovery, especially marine antibiotics with infinite potential. The ‘Netflix’ subscription model, which is used for antibiotics prescribed for BPPL infections and can provide financial guarantee for pharmaceutical companies, may also prove to be an effective way to develop novel marine antibiotics against *S. aureus* (Fig. 11)*.*

## Conclusions and prospects

**Fig. 12:**
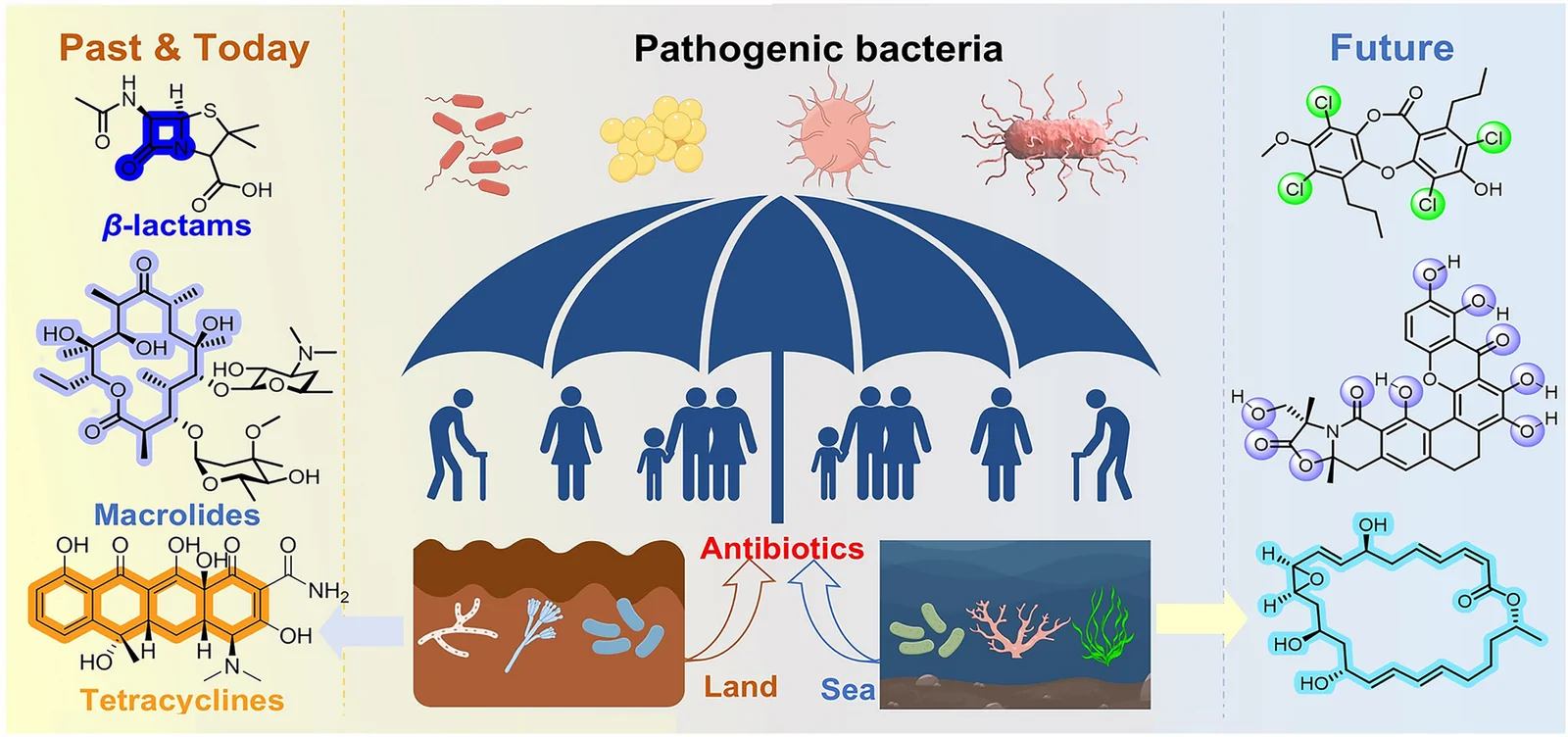
The source of antibiotics in the past and future (By Figdraw)

Vancomycin has been used as the last line of defense against MRSA infections since its discovery. However, vancomycin-resistant *Staphylococcus aureus* is now showing a global epidemic trend. Therefore, it is urgent to proactively plan strategies to deal with antibiotic resistance. Notably, MNPs, a treasure trove provided by nature, hold significant potential to expand the scope and novelty of antibacterial drug discovery pipelines and reinforce the therapeutic arsenal against drug-resistant microorganisms. However, developing novel antibiotics for MRSA and other resistant pathogens remains a major challenge. First, understanding the pharmacological properties and in vivo behavior of active compounds is essential to ensure they pass preclinical and clinical evaluation. For example, narrow or broad antibacterial spectrum, organ-specific tissue distribution, and ability to cross the blood–brain or blood-placenta barriers. Second, the needs of workers in extreme environments, such as marine personnel, must be considered. High temperature, humidity, salinity, and unstable sea conditions require drugs with corrosion-resistant packaging, compact storage, and oral formulations. Finally, antibiotic resistance spans multiple fields—medicine, pharmacy, environmental science, food safety, and aquaculture—and requires interdisciplinary collaboration. In the future, based on marine natural products and guided by the major challenges of antibacterial drugs, it will inject new strength into the research and development of antibacterial drugs (Fig. 12).

## Supplementary Information

Below is the link to the electronic supplementary material.Supplementary file1 (PDF 5034 KB)

## Data Availability

Additional data related to this paper may be requested from the authors.
