# Supplementary material for: Breaking the antimicrobial resistance dilemma: marine natural products, a treasure trove for the development of new anti-Staphylococcus aureus drugs
Source: Mar Life Sci Technol. 2026 Feb 2;8(3):659–79. doi: 10.1007/s42995-026-00355-8 (PMC13525127; doi:10.1007/s42995-026-00355-8)
Supplement: Supplementary file 1 [file 42995_2026_355_MOESM1_ESM.pdf]

Supporting Information

Breaking the Antimicrobial Resistance Dilemma: Marine natural products, a treasure trove for the development of new anti-*Staphylococcus aureus* drugs

Peng-Jie Li [1], Liu-Xia Lv [1], Xi-Zhen Cao [1], Jian-Yu Liu [1], Ji-Xiu Gao [1], Hao-Ran Li [1], Gulab Said [2], Dan Chen [3, *], Bo Yao [4, *], Xin Liu [5, 6, *], Mei-Yan Wei [1, *], Xiao-Ping Yang [7, *], Tong-Yi Xu [8, *], Yu-Cheng Gu [9, *], Chang-Lun Shao [1, 10, *]

1 Key Laboratory of Marine Drugs, The Ministry of Education of China, School of Medicine and Pharmacy, Ocean University of China, Qingdao 266003, People's Republic of China

2 Department of Chemistry, Women University Swabi, Swabi 23430, Pakistan

3 Hospital of Ocean University of China, Qingdao 266003, People's Republic of China

4 Department of Critical Care Medicine, Qilu Hospital (Qingdao), Cheeloo College of Medicine, Shandong University, 758 Hefei Road, Qingdao, Shandong, 266035, People's Republic of China

5 Science and Technology Research Center of Chinese Customs, Beijing 100026, People's Republic of China

6 School of Bioengineering, Beijing Polytechnic University, Beijing 100176, People's Republic of China

7 Qingdao Traditional Chinese Medicine Hospital, Qingdao Hiser Hospital Affiliated of Qingdao University, Qingdao 266100, People's Republic of China

8 Department of Cardiovascular and Thoracic Surgery, No. 971 Hospital of PLA Navy, Qingdao 266071, People's Republic of China

9 Syngenta Jealott's Hill International Research Centre, Bracknell, Berkshire, RG42 6EY, United Kingdom

10 Key Laboratory of Tropical Medicinal Resource Chemistry of Ministry of Education, College of Chemistry and Chemical Engineering, Hainan Normal University, Haikou 571158, People's Republic of China

List of Supporting Information

Supplementary Fig. S1 The chemical structures of compounds **1–63**
Supplementary Fig. S2 The chemical structures of compounds **64–89**
Supplementary Fig. S3 The chemical structures of compounds **90–124**
Supplementary Fig. S4 The chemical structures of compounds **125–142**
Supplementary Fig. S5 The chemical structures of compounds **143–161**
Supplementary Fig. S6 The chemical structures of compounds **162–165**
Supplementary Fig. S7 The chemical structures of compounds **166–189**
Supplementary Fig. S8 The chemical structures of compounds **190–210**
Supplementary Fig. S9 The chemical structures of compounds **211–216**
Supplementary Fig. S10 The chemical structures of compounds **217–242**
Supplementary Table. S1 Antibiotics marketed from 2003 to 2025
Supplementary Table. S2 Anti-*S. aureus* compounds reported from 1977 to 2025
Supplementary Table. S3 The source distribution of marine bacterial-derived active compounds

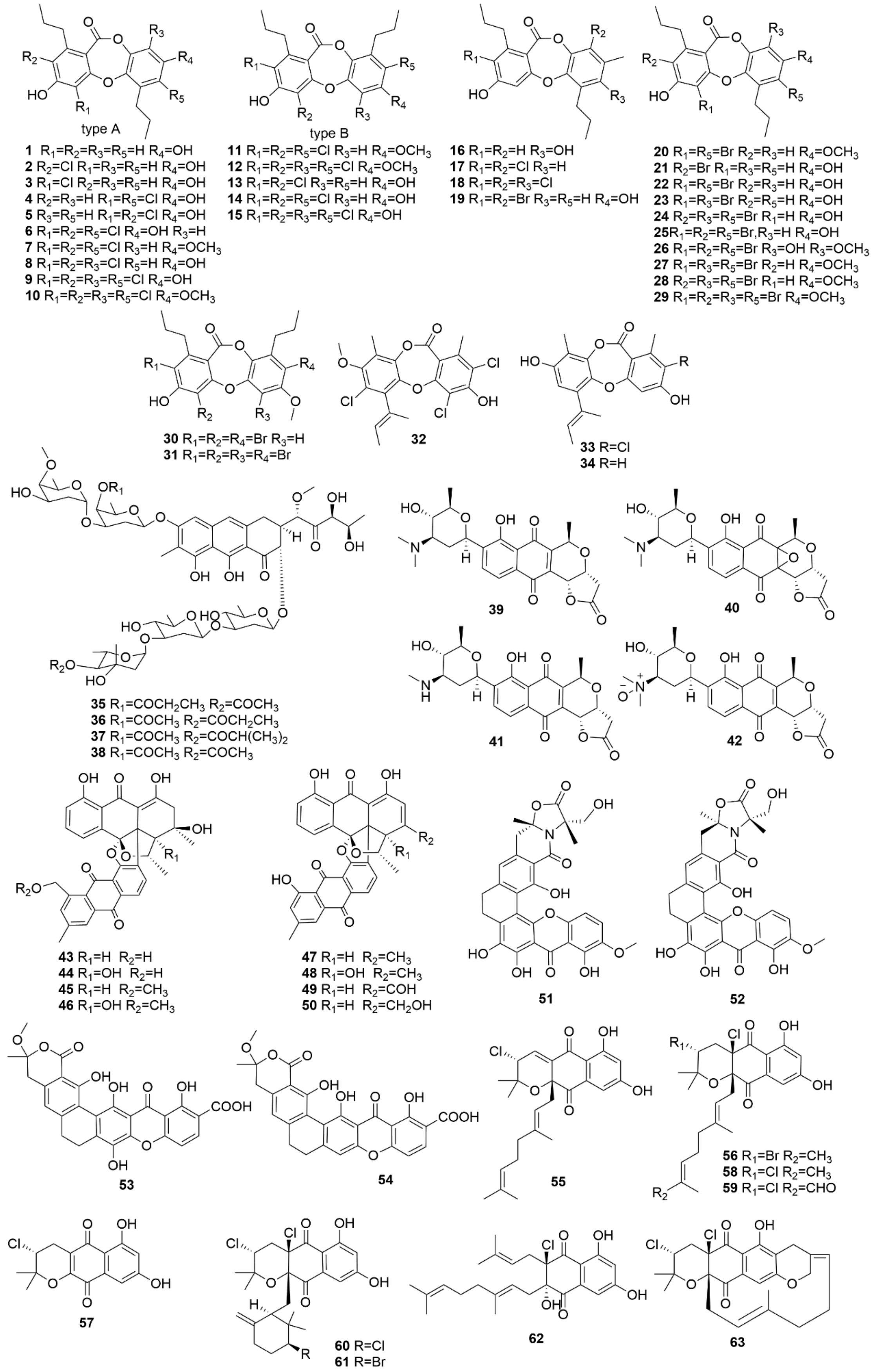

Supplementary Fig. S1 The chemical structures of compounds **1–63.**

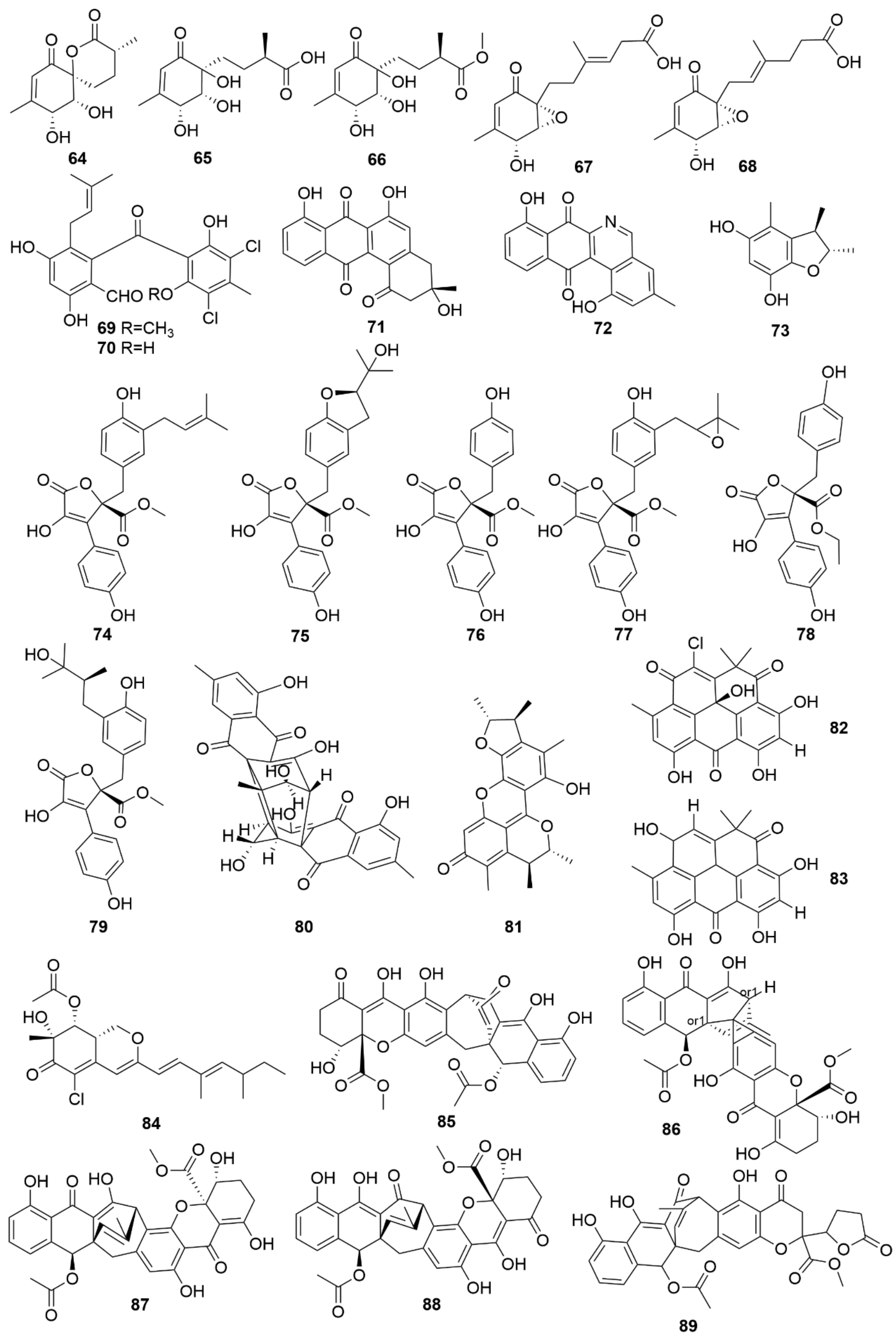

Supplementary Fig. S2 The chemical structures of compounds **64–89.**

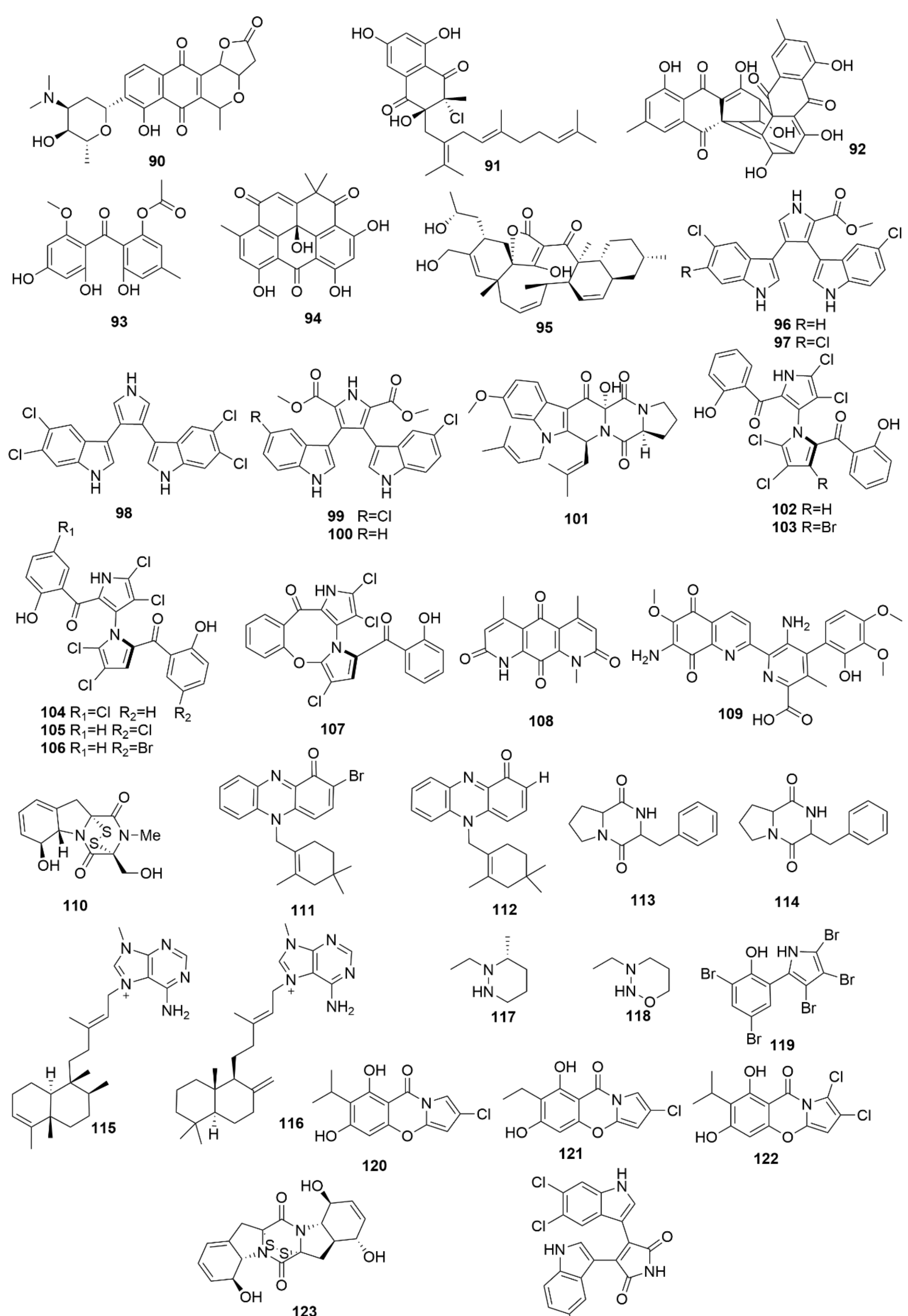

Supplementary Fig. S3 The chemical structures of compounds **90–124.**

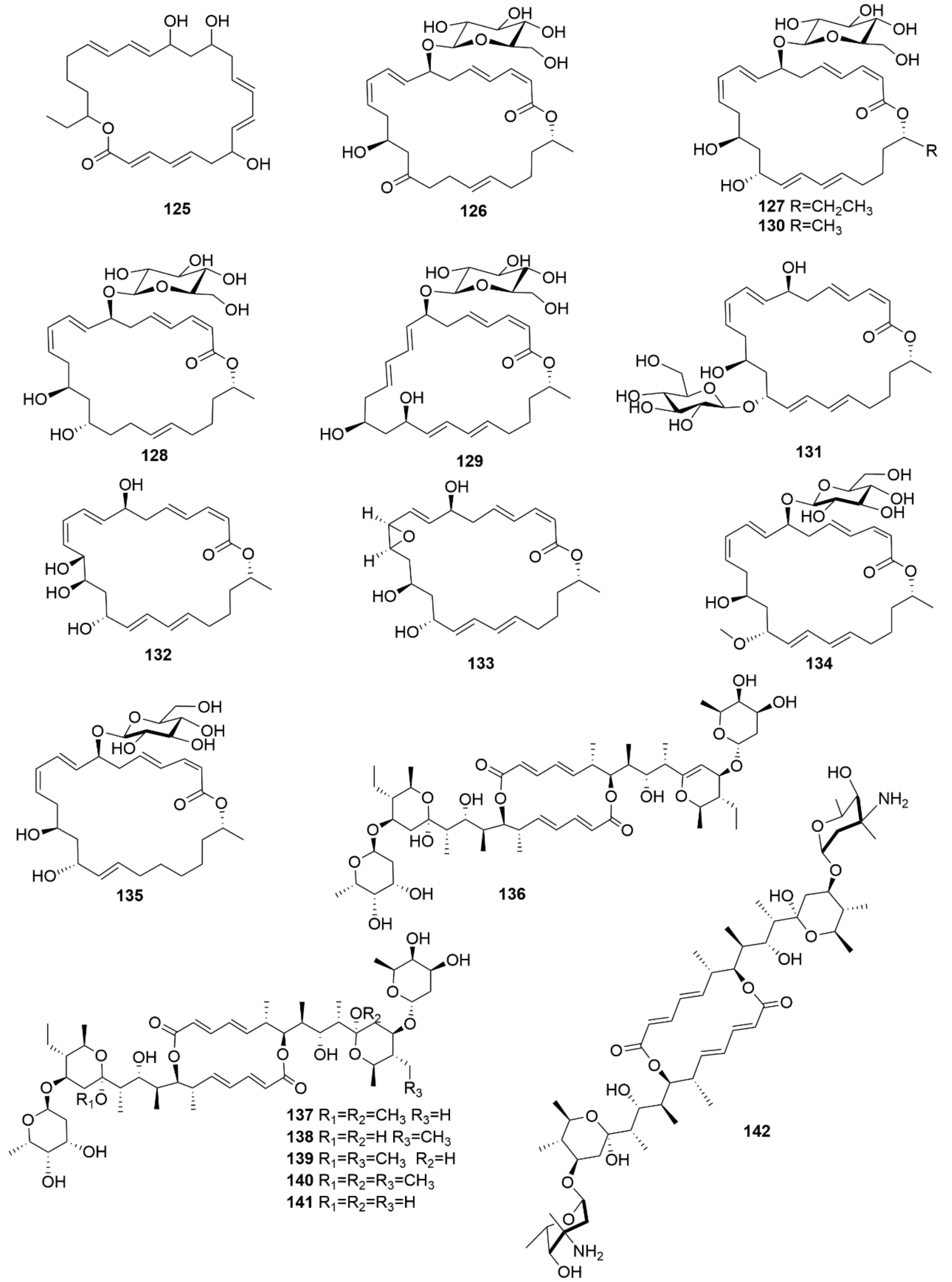

Supplementary Fig. S4 The chemical structures of compounds **125–142.**

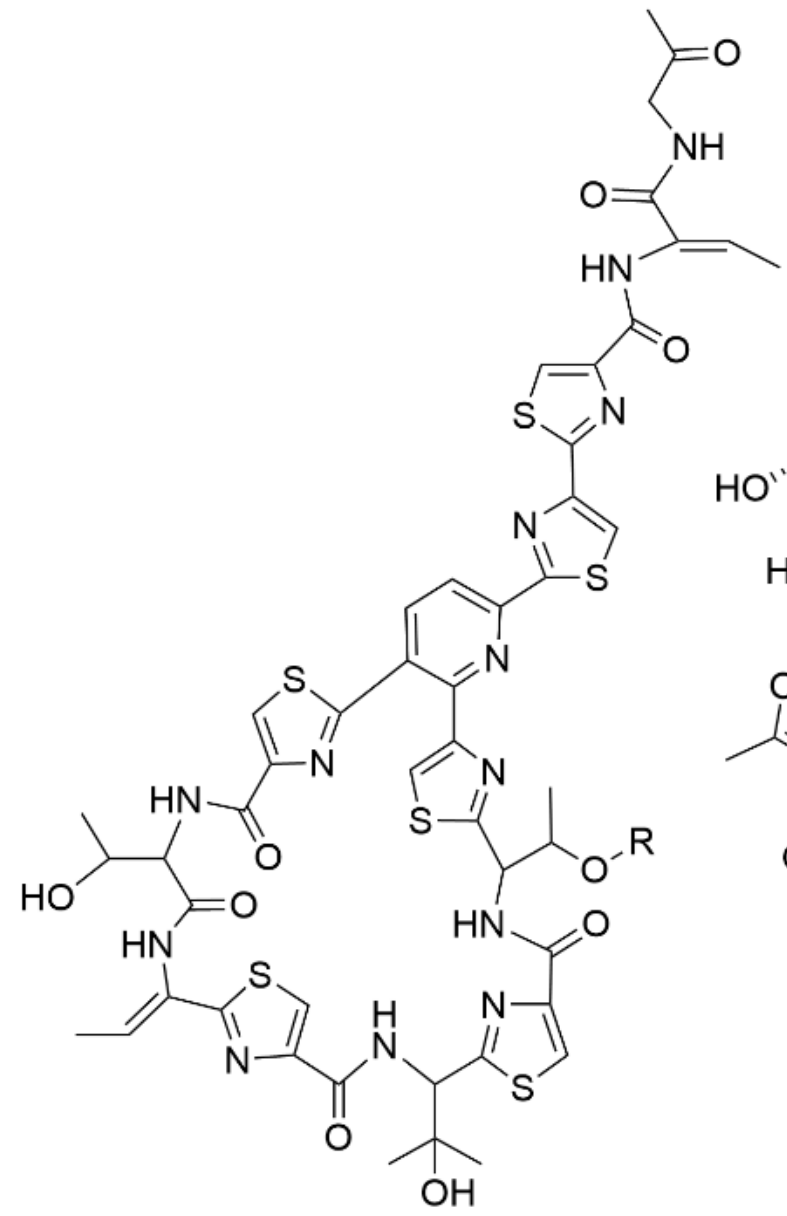

143 R=H
144 R=CH_3

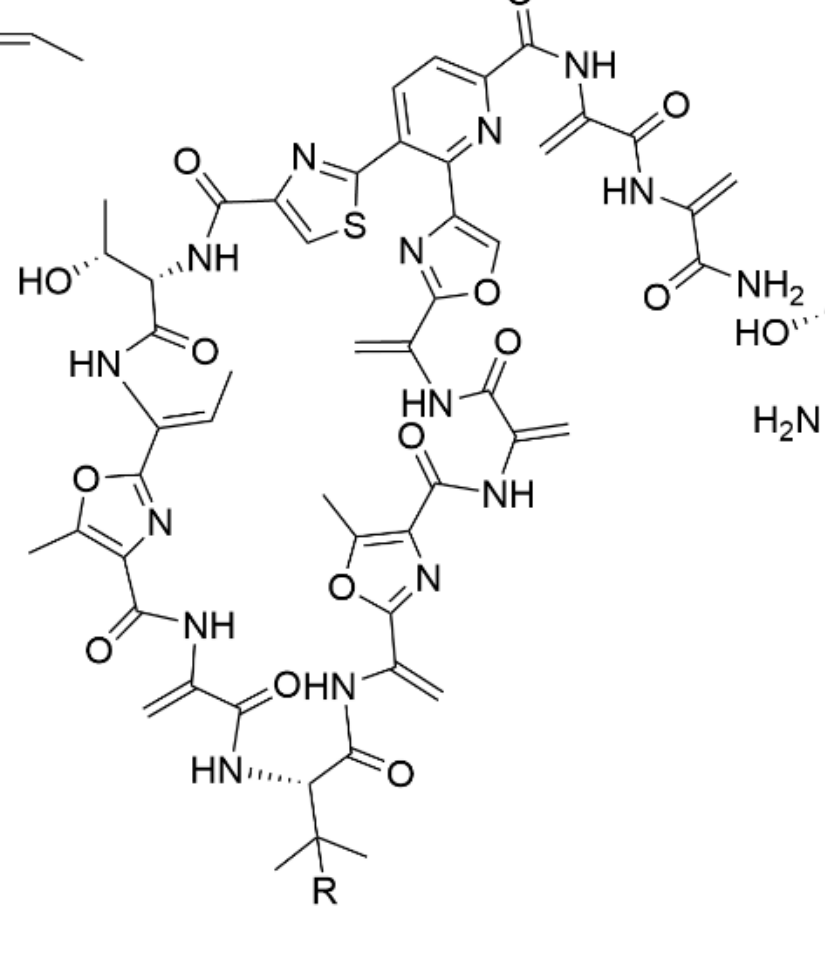

145 R=OH
146 R=H

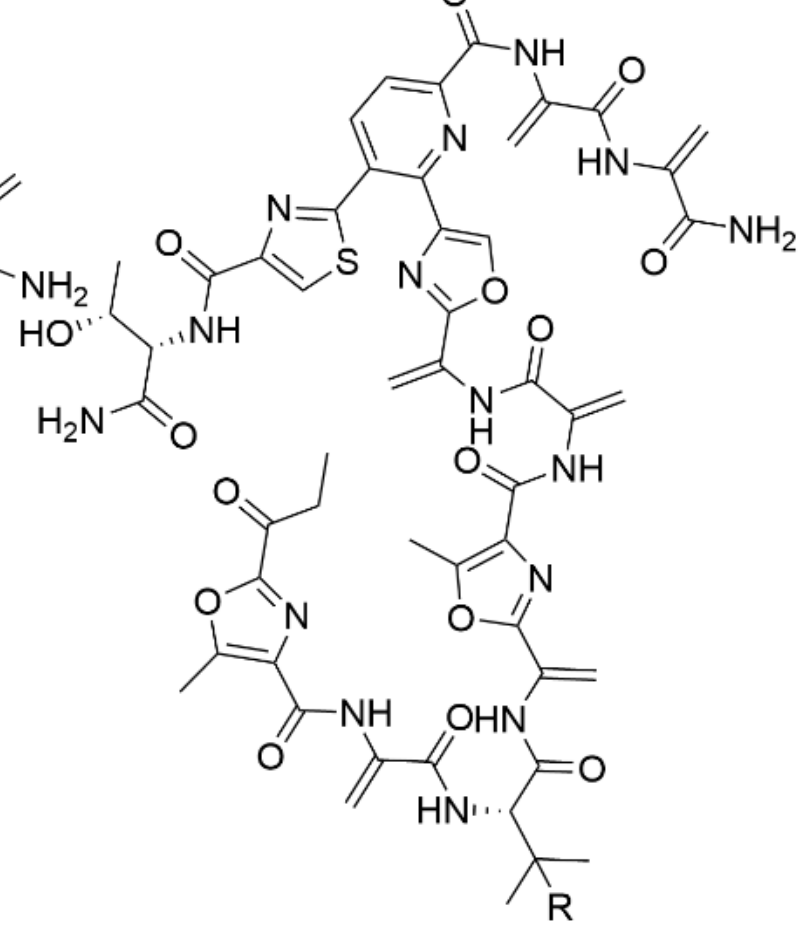

147 R=H
148 R=OH

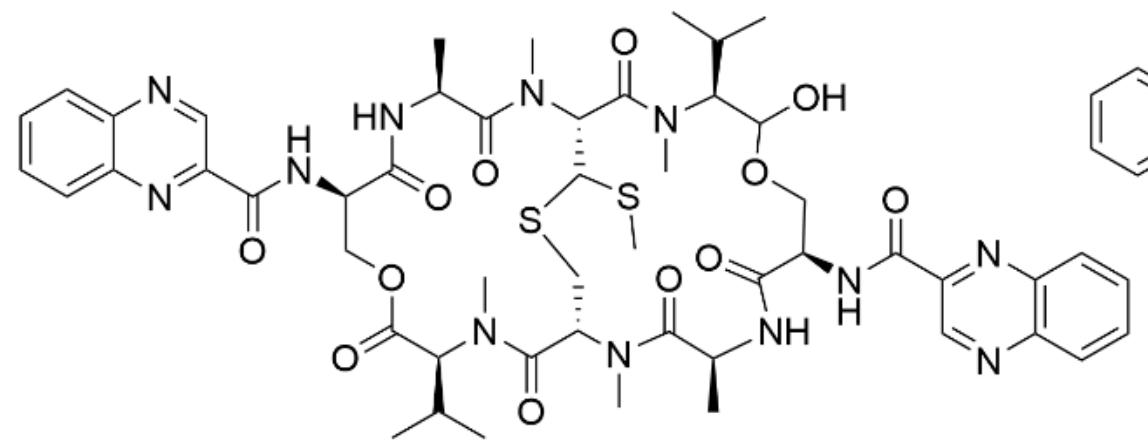

149

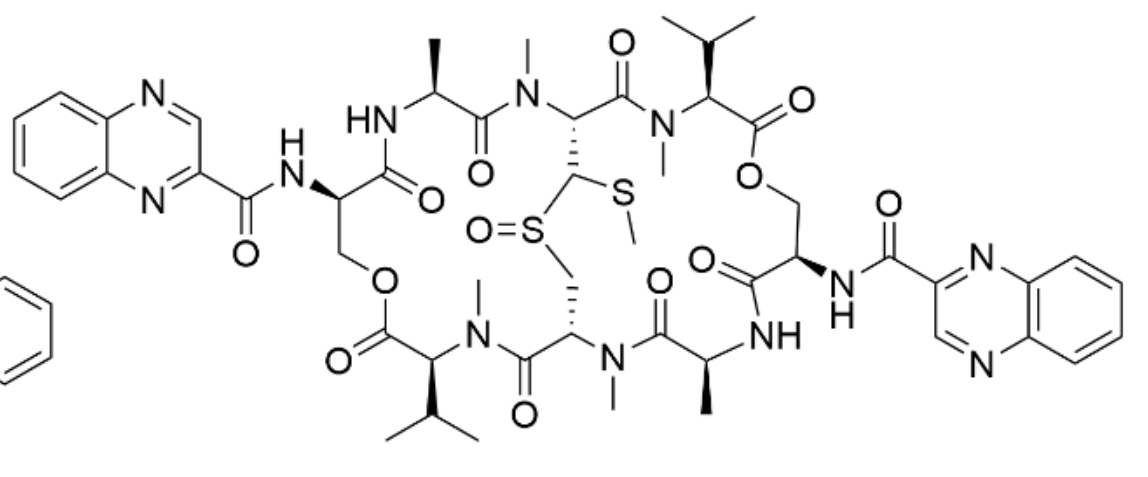

150

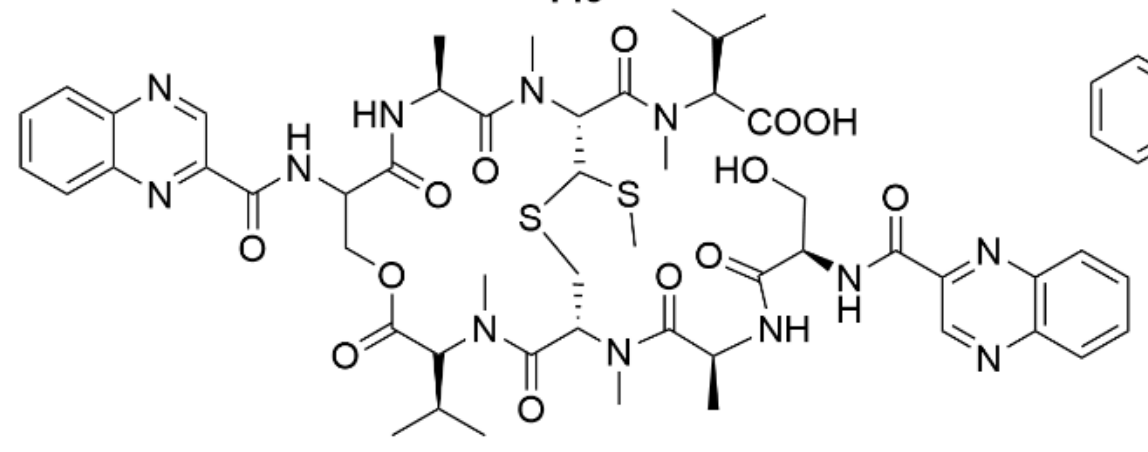

151

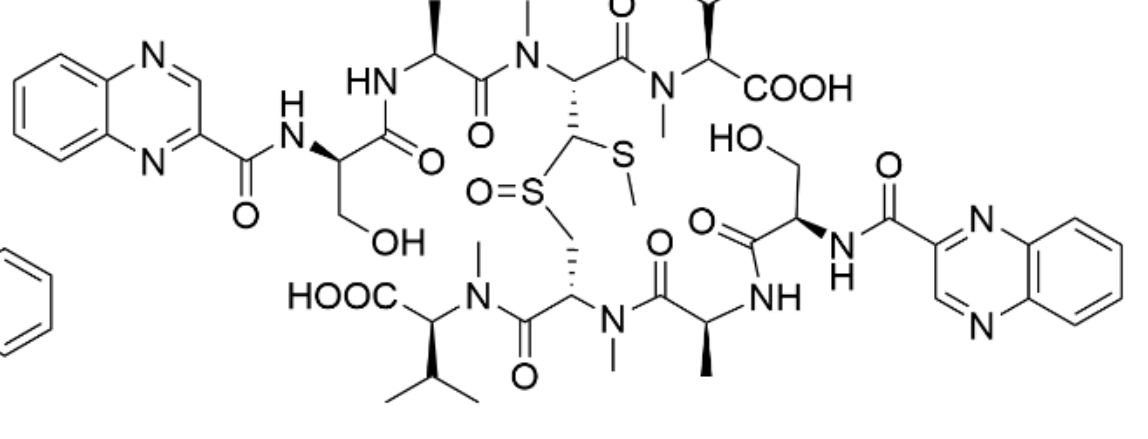

152

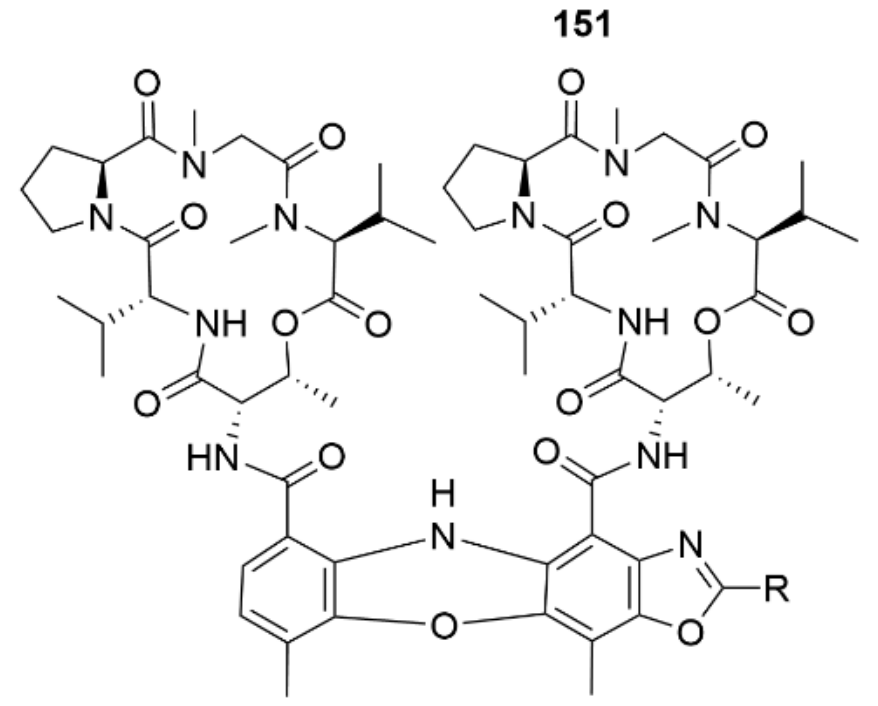

153 R=H
154 R=*iso*-butyl

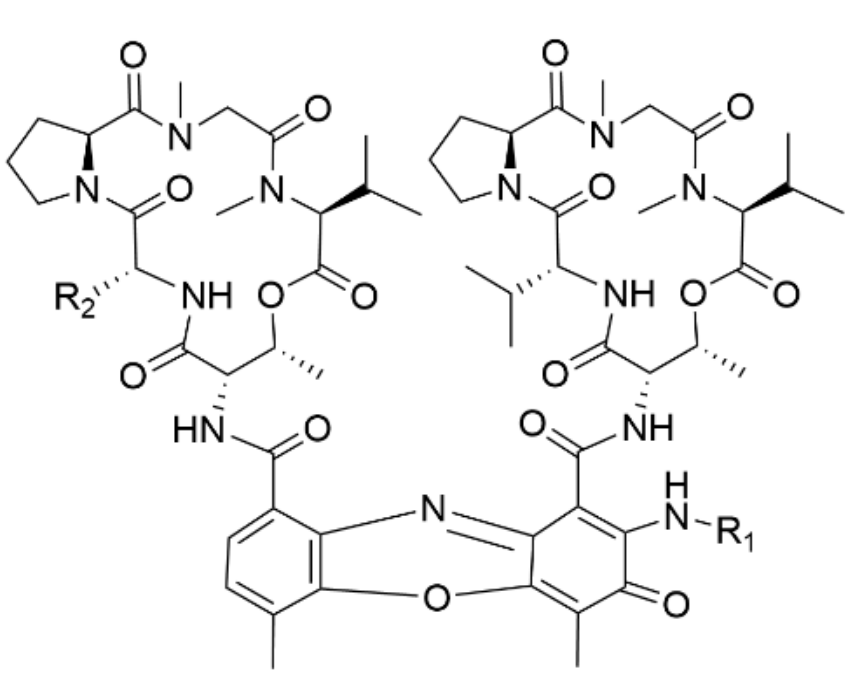

155 R_1=Ac R_2=*iso*-propyl
156 R_1=H R_2=CH_3
157 R_1=H R_2=*iso*-propyl

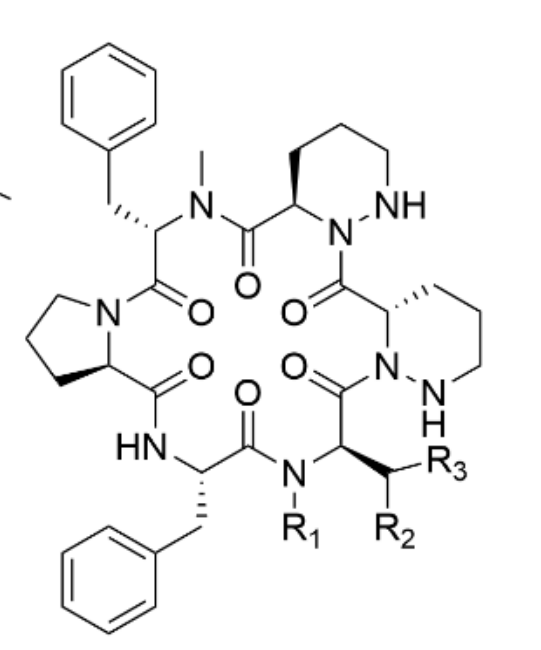

158 R_1=H R_2=CH_3 R_3=C_2H_5
159 R_1=OH R_2=CH_3 R_3=C_2H_5
160 R_1=OH R_2=CH_3 R_3=CH_3
161 R_1=OH R_2= R_3=$CH(CH_3)_2$

Supplementary Fig. S5 The chemical structures of compounds **143–161.**

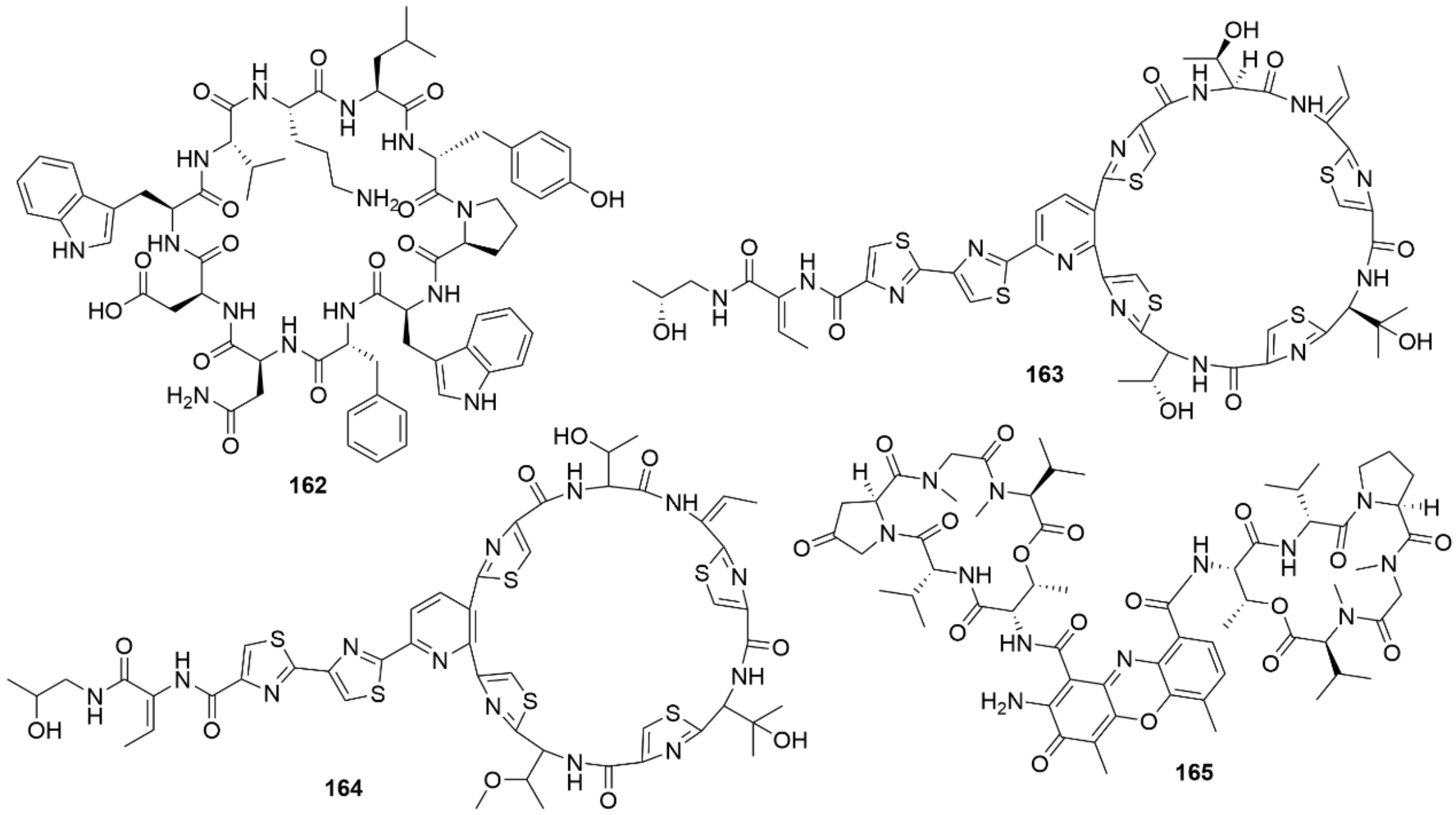

Supplementary Fig. S6 The chemical structures of compounds **162–165.**

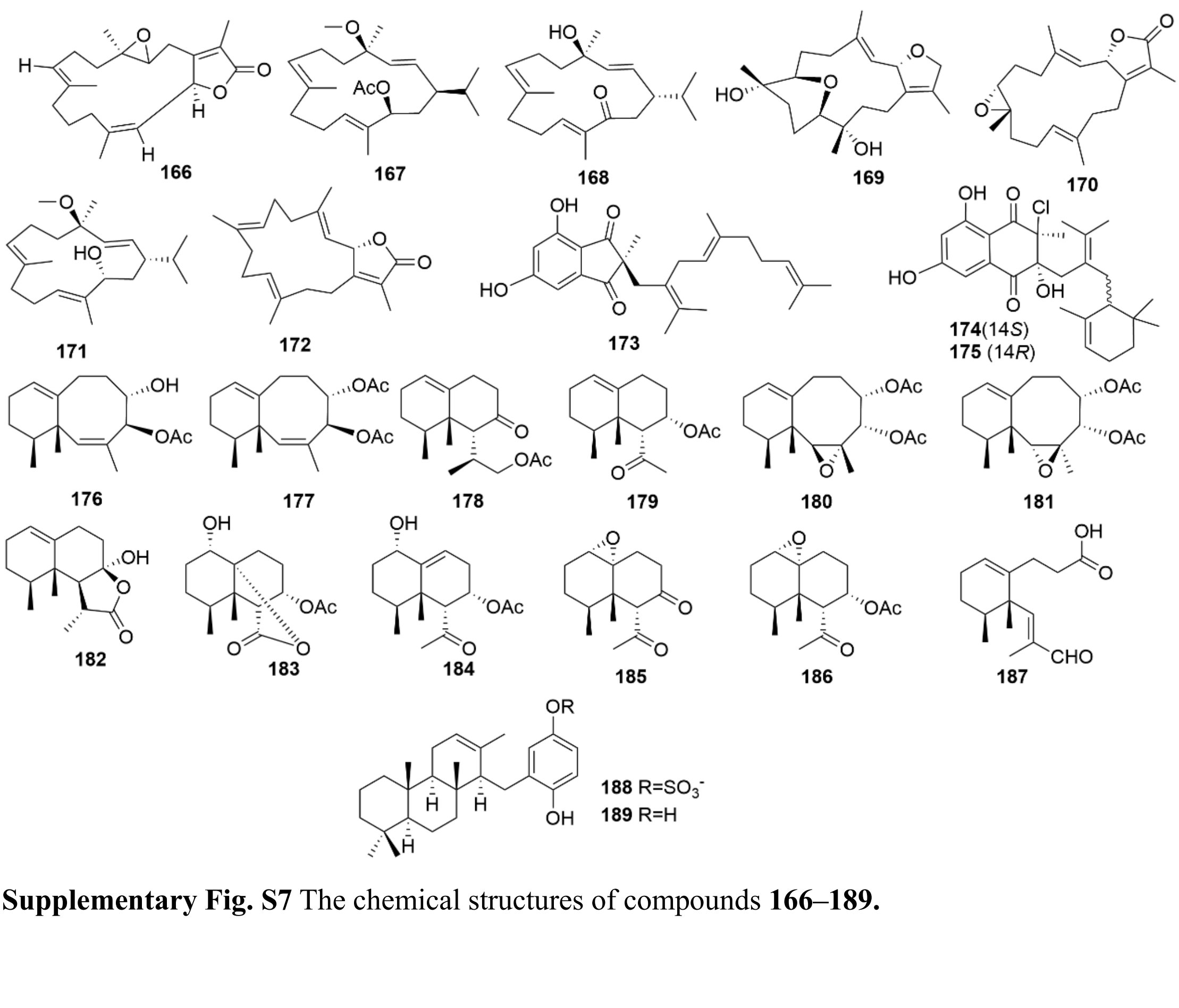

Supplementary Fig. S7 The chemical structures of compounds **166–189.**

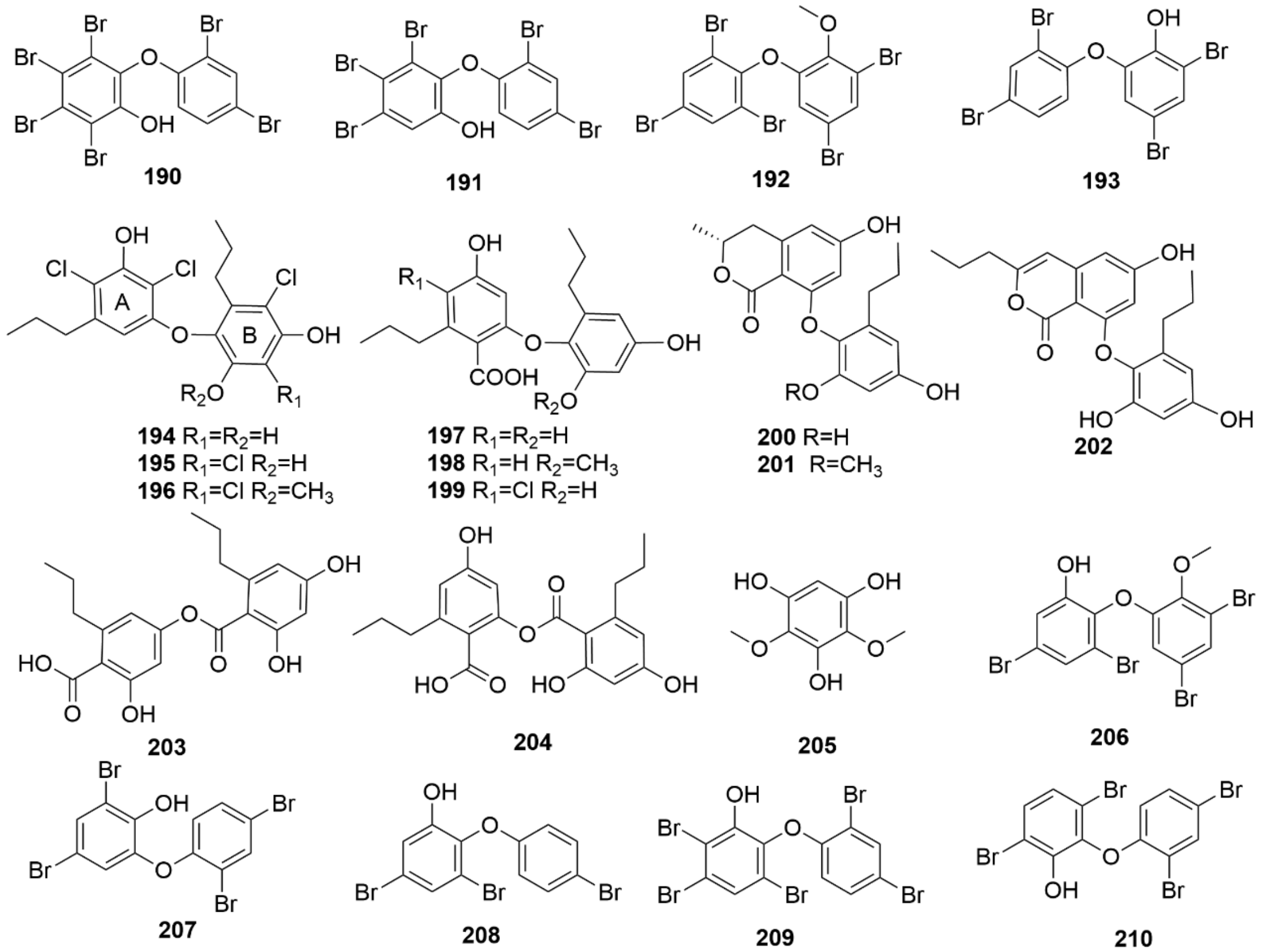

Supplementary Fig. S8 The chemical structures of compounds **190–210.**

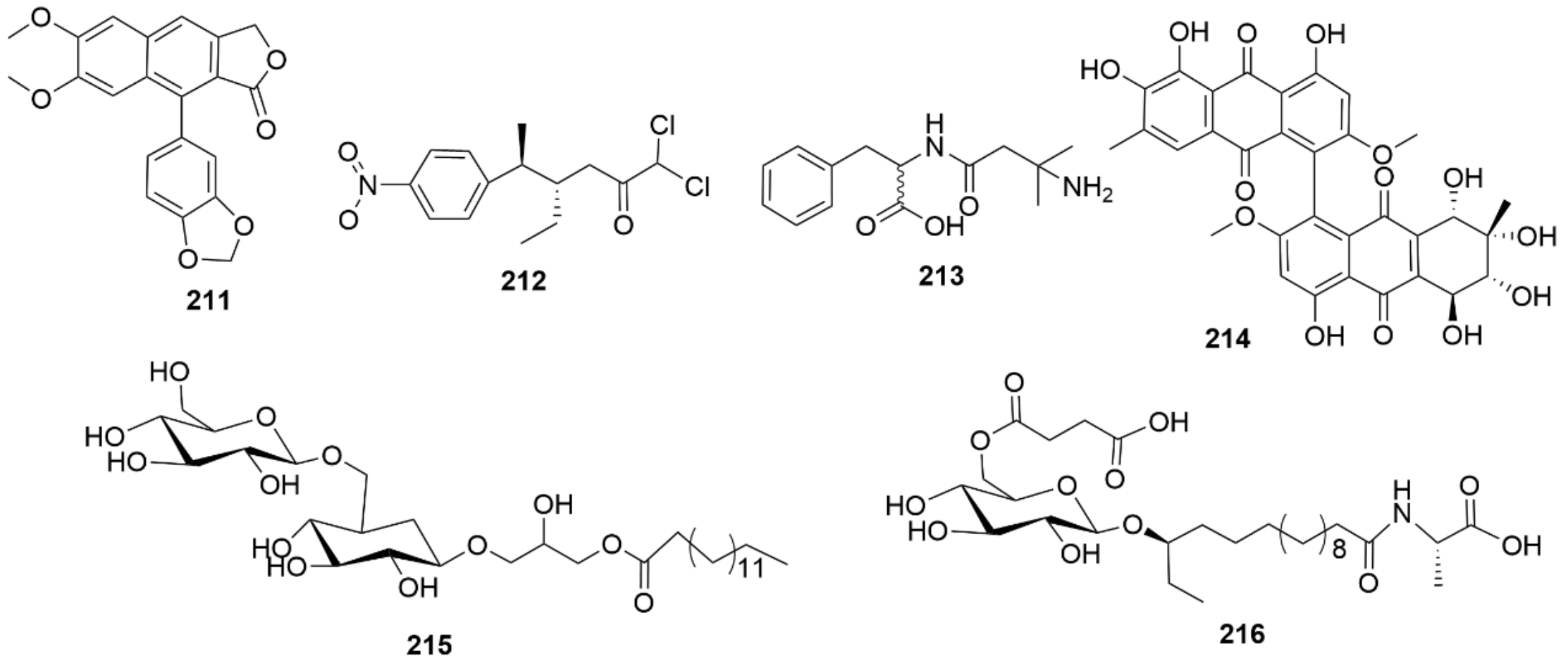

Supplementary Fig. S9 The chemical structures of compounds **211–216.**

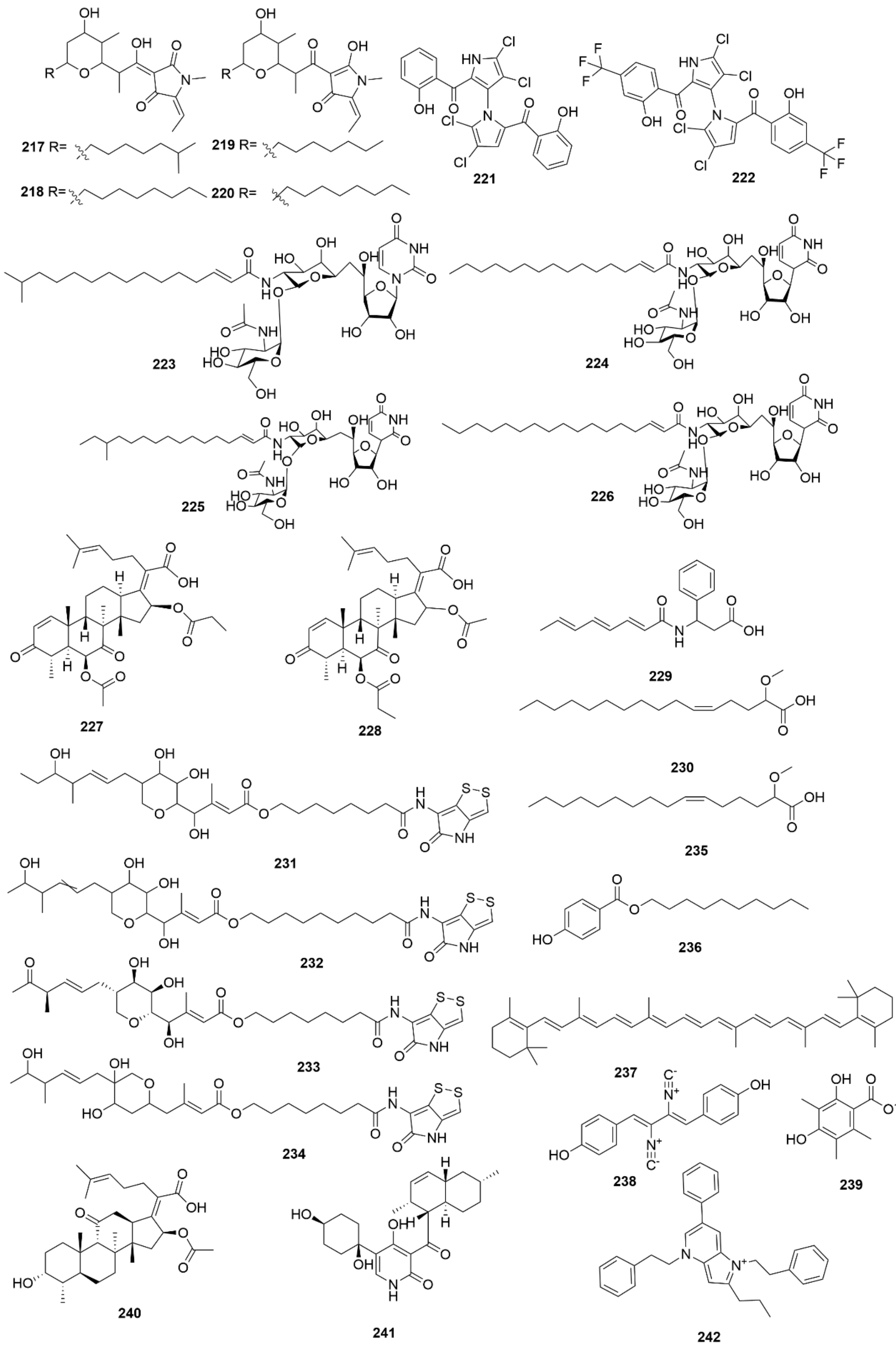

Supplementary Fig. S10 The chemical structures of compounds **217–242.**

Supplementary Table S1. Antibiotics marketed from 2003 to 2025

Name	Clinical Indications	Molecular Mechanism	Type	Year
Prulifloxacin	Bronchitis Respiratory tract infections	DNA gyrase inhibitor		2004
Sitafloxacin hydrate	Gastroenteritis Gram-negative bacterial infection Pneumonia	DNA gyrase inhibitor, DNA topoisomerase IV inhibitor		2008
Besifloxacin hydrochloride	Ocular bacterial infection	DNA gyrase inhibitor, DNA topoisomerase IV inhibitor		2009
Nemonoxacin	Diabetic complications PneumoniaGram-positive bacterial infection	DNA topoisomerase IV inhibitor		2016
Pazufloxacin mesylate	Conjunctivitis, otitis media Pneumonia	DNA gyrase inhibitor, DNA topoisomerase IV inhibitor		2014
Gemifloxacin mesylate	Acute rhinosinusitis Gram-negative bacterial infection Pneumonia	DNA gyrase inhibitor, DNA topoisomerase IV inhibitor		2003
Garenoxacin mesylate	Respiratory tract infections Pneumonia	DNA gyrase inhibitor, DNA topoisomerase IV inhibitor	Quinolones	2007
Zabofloxacin hydrochloride	Bronchitis Gram-positive bacterial infection Pneumonia	DNA gyrase inhibitor, DNA topoisomerase IV inhibitor		2015
Ozenoxacin	Acne Impetigo	DNA gyrase inhibitor, DNA topoisomerase IV inhibitor		2017
Delafloxacin meglumine	Gonorrhea Acute bacterial skin and skin structure infections	DNA gyrase inhibitor, DNA topoisomerase IV inhibitor		2017
Levonadifloxacin arginine salt	Acute bacterial skin and skin structure infections	DNA gyrase inhibitor, DNA topoisomerase IV inhibitor		2020
Alalevonadifloxacin mesylate	Acute bacterial skin and skin structure infections	DNA gyrase inhibitor, DNA topoisomerase IV inhibitor		2019
Lascufloxacin hydrochloride	Pneumonia	DNA gyrase inhibitor, DNA topoisomerase IV inhibitor		2019

Finafloxacin	Acute otitis externa	DNA gyrase inhibitor, DNA topoisomerase IV inhibitor		2014
Antofloxacin	BronchitisAcute pyelonephritis	DNA gyrase inhibitor		2009
Balofloxacin	Urethritis Urinary tract infection	DNA gyrase inhibitor		2003
Tigecycline	Acute bacterial skin and skin structure infections Gram-negative bacterial infection	Ribosomal protein inhibitor		2005
Sarecycline hydrochloride	Anthrax, cystitis, rosacea	Ribosomal protein inhibitor	Tetracyclines	2019
Omadacycline	Acute bacterial skin and skin structure infections	Ribosomal protein inhibitor		2018
Eravacycline	Complicated urinary tract infection	Ribosomal protein inhibitor		2018
Plazomicin	Gram-negative bacterial infection Pyelonephritis	Ribosomal protein inhibitor	Aminoglycosides	2018
Linezolid	Skin and soft tissue infections Bacterial pneumonia Tuberculosis	Monoamine oxidase inhibitor Ribosomal protein inhibitor		2000
Tedizolid	Acute bacterial skin and skin structure infection	Breast cancer-resistant protein inhibitor Monoamine oxidase inhibitor	Oxazolidines	2014
Contezolid	Complex skin and soft tissue infections	Monoamine oxidase inhibitor		2021
Retapamulin	Phagocytosis caused by *Staphylococcus aureus* or *Streptococcus pyogenes*	Ribosomal protein inhibitor		2007
Lefamulin	Acute bacterial skin and skin structure infections Pneumonia	Ribosomal protein inhibitor	Pleuromutilins	2019
Telithromycin	Pneumonia	Ribosomal protein inhibitor	Macrolides	2001

Fidaxomicin*	Clostridioides difficile-associated diarrhea infection MDR bacterial infection	DNA-directed RNA polymerase inhibitor		2014
Ceftaroline fosamil acetate	Osteomyelitis, sepsis, bacteremia Acute bacterial skin and skin structure infections, pneumonia	Penicillin-binding protein inhibitor		2010
Ceftobiprole medocaril	Bacteremia, neutropenia, Pneumonia Acute bacterial skin and skin structure infections	Penicillin-binding protein inhibitor		2008
Cefiderocol*	Complicated urinary tract infection Pyelonephritis	Penicillin-binding protein inhibitor	β-lactams	2019
Ertapenem	Endometritis Acute bacterial skin and skin structure infections	Penicillin-binding protein inhibitor		2001
Biapenem	Sepsis, pneumonia, lung abscess	Penicillin-binding protein inhibitor		2002
Doripenem	Bacterial meningitis Fibrosis, pneumonia	Dehydropeptidase I inhibitor Penicillin-binding protein inhibitor		2005
Tebipenem pivoxil	Gram-negative bacterial infection	Penicillin-binding protein inhibitor		2009
Ceftolozane/tazobactam	Complicated urinary tract infection Complicated intra-abdominal infection	Penicillin-binding protein inhibitor+β-lactamase inhibitor		2014
Ceftazidime/avibactam	Complicated urinary tract infection Complicated intra-abdominal infection	Penicillin-binding protein inhibitor+β-lactamase inhibitor	β-lactams + BLI	2015
Meropenem/vaborbactam	Complicated urinary tract infection	β-lactamase inhibitor		2017

Imipenem/cilastatin/relebactam	Complicated urinary tract infection Complicated intra-abdominal infection	Penicillin-binding protein inhibitor +β-lactamase inhibitor		2019
Daptomycin	*S. aureus*-caused bloodstream infection accompanied by infective endocarditis	Peptidoglycan synthesis inhibitor	Cyclic lipopeptide	2003
Sulbactam/durlobactam*	-	β-lactamase inhibitor	BLI	2023
Pretomanid (Dovprela) *	XDR-TB	DNA synthesis inhibitor	Nitroimidazole	2019

*：Narrow-spectrum antibiotic

Supplementary Table S2. Anti-*S. aureus* compounds reported from 1977 to 2025

No.	Compound	Origin	Type	MSSA	MRSA	Ref.
				MIC (μg/mL)		
1	2-n-pentyl-4-quinolinol	bacteria	alkaloid	-	-	(Wratten et al. 1977)
2	2-n-heptyl-4-quinolinol	bacteria	alkaloid	-	-	
3	diisocyanide	sponge	terpenoid	-	-	(Wratten et al. 1978)
4	formamid	sponge	terpenoid	-	-	
5	seco-manoalide	sponge	terpenoid	-	-	(De Silva et al. 1981)
6	*E*-neomanoalide	sponge	terpenoid	-	-	
7	*Z*-neomanoalide	sponge	terpenoid	-	-	
8	manoalide	sponge	terpenoid	-	-	(De Silva et al. 1980)
9	garveatin A	hydrop	polyketide	-		(Fahy et al. 1985)
10	spongionellin	sponge	terpenoid	-	-	(Kato et al. 1985)
11	dehydrospongionellin	sponge	terpenoid	-	-	
12	palmiromicin	bacteria	other	-	-	(Mal'tsev et al. 1991)
13	B-1015	bacteria	alkaloid	12.5	-	(Inukai et al. 1993)
14	aeroplysinin-1	sponge	phenol	-	-	(Teeyapant et al. 1993)
15	dienone	sponge	polyketide	-	-	
16	andrimid	bacteria	alkaloid	4	2	(Needham et al. 1994)
17	moiramide A	bacteria	alkaloid	2	0.5	
18	bastadin 8	sponge	alkaloid	-	-	(Pettit et al.

						1995)
19	bastadin 10	sponge	alkaloid	-	-	
20	bastadin 12	sponge	alkaloid	-	-	
21	N-ethylenemethyl ketone derivative of renierone	sponge	alkaloid	-	-	(Edrada, Proksch, Wray, Christ, et al. 1996)
22	1,6-dimethyl-7-methoxy-5,8-dihydroisoquinoline-5,8-dione	sponge	alkaloid	-	-	
23	renierone	sponge	alkaloid	-	-	
24	mimosamycin	sponge	alkaloid	-	-	
25	manzamine A	sponge	alkaloid	-	-	(Edrada, Proksch, Wray, Witte, et al. 1996)
26	macrolactin M	bacteria	macrolide	0.38	-	(Nagao et al. 1997)
27	TMD	bacteria	alkaloid	＜0.01	＜0.01	(Shiozawa et al. 1997)
28	TME	bacteria	alkaloid	＜0.01	＜0.01	
29	TMF	bacteria	alkaloid	＜0.01	＜0.01	
30	TMG	bacteria	alkaloid	＜0.01	＜0.01	
31	(*Z*)-2-methoxy-5-hexadecenoic acid	sponge	aliphatic acid	0.09	-	(Carballeira et al. 1998)
32	(*Z*)-2-methoxy-6-hexadecenoic acid	sponge	aliphatic acid	0.095	-	
33	2-chloro-6,8-dihydroxy-7-isopropyl-9H-benzo[e]pyrrolo[2,1-b][1,3]oxazin-9-one	bacteria	alkaloid	0.2−0.78	-	(Olson et al. 1998)
34	2-chloro-7-ethyl-6,8-dihydroxy-9H-benzo[e]pyrrolo[2,1-b][1,3]oxazin-9-one	bacteria	alkaloid	0.78−1.56	-	
35	1,2-dichloro-6,8-dihydroxy-7-isopropyl-9H-benzo[e]pyrrolo[2,1-b][1,3]oxazin-9-one	bacteria	alkaloid	0.78−1.56	-	
36	1,2-dichloro-7-ethyl-6,8-dihydroxy-9H-benzo[e]pyrrolo[2,1-b][1,3]oxazin-9-one	bacteria	alkaloid	3.125−12.5	-	

37	7-butyl-2-chloro-6,8-dihydroxy-9H-benzo[e]pyrrolo[2,1-b][1,3]oxazin-9-one	bacteria	alkaloid	1.56	-	
38	sesterstatin 2	sponge	terpenoid	-	-	(Pettit et al. 1998)
39	loloatin A	bacteria	peptide	4	4	(Gerard et al. 1999)
40	loloatin B	bacteria	peptide	2	2	
41	loloatin C	bacteria	peptide	0.5	0.5	
42	loloatin D	bacteria	peptide	8	8	
43	ilimaquinone	sponge	terpenoid	3.12	-	(Popov et al. 1999)
44	chiatoquinone	sponge	terpenoid	6.25	-	
45	lsospongiaquinone	sponge	terpenoid	12.5	-	
46	dicb'oceratidaquinone	sponge	terpenoid	12.5	-	
47	puupehenone	sponge	terpenoid	1.56	-	
48	chlorpuupehenone	sponge	terpenoid	12.5	-	
49	2,3,4,5-tetrabromo-6-(2,4-dibromophenoxy)phenol	sponge	phenol	0.36	-	
50	3,4,5-tribromo-2-(2,4-dibromophenoxy)phenol	sponge	phenol	0.36	-	
51	3,5-dibromo-2-(3,5-dibromo-2-methoxyphenoxy)phenol	sponge	phenol	0.15	-	
52	matemone	sponge	alkaloid	-	-	(Carletti et al. 2000)
53	12-epi-deoxoscalarin	sponge	steroid	-	-	(Erdogan et al. 2000)
54	4-hydroxy-3-tetraprenylaceticacid	sponge	phenol	-	-	
55	2,4-dibromo-6-chlorophenol	bacteria	phenol	-	-	(Jiang et al. 2000)
56	sumiki’s acid	fungus	other	-	-	
57	acetyl Sumiki’s acid	fungus	other	-	-	
58	pestalone	fungus	polyketide	-	0.037#	
59	bogorol A	bacteria	peptide	-	2	(Barsby et al. 2001)
60	arenosclerin A	sponge	alkaloid	112	10−100	(Torres et al. 2002)
61	arenosclerin B	sponge	alkaloid	100	5−100	
62	arenosclerin C	sponge	alkaloid	25	5−100	
63	haliclonacyclamine E	sponge	alkaloid	100	10−50	
64	lunatin	fungus	polyketide	-	-	(Jadulco et

						al. 2002)
65	cytoskyrin A	fungus	polyketide	-	-	
66	aurcuphenol	sponge	terpenoid	7.5−25	7.5−50	(El Sayed et al. 2002)
67	Sch 575948	sponge	alkaloid	-	-	(Yang et al. 2003)
68	(all *Z*)-2′-eicosa-5,8,11,14,17-pentaenoylphloroglucinol	brown algae	phenol	-	-	(Wisespong pand et al. 2003)
69	(all *Z*)-2′-eicosa-17 (*R*)–hydroxy-5,8,11,14-tetraenoylphloroglucinol	brown algae	phenol	-	-	
70	(5*Z*,8*Z*,11*Z*,13*E*,17*Z*)-2′-eicosa-15 (*S*)– hydroxy-5,8,11,13,17 –pentaenoylphloroglucinol	brown algae	phenol	-	-	
71	melophlin C	sponge	alkaloid	-	-	(Wang et al. 2003)
72	YM-266183	bacteria	peptide	0.025	1	(Nagai et al. 2003)
73	YM-266184	bacteria	peptide	0.025	0.39	
74	thiocillin I	bacteria	peptide	0.05	0.78	
75	thiocillin II	bacteria	peptide	0.013	0.2	
76	C03	other	peptide	50	-	(Mei et al. 2003)
77	7-deacetoxyyanuthone A	fungus	polyketide	50	-	(Li et al. 2003)
78	2,4-diacetylphloroglucinol	bacteria	phenol	-	1−4	(Isnansetyo, Cui, et al. 2003; Isnansetyo et al. 2001; Kamei et al. 2003)
79	MC21-A	bacteria	biphenyl	-	1−2	(Isnansetyo & Kamei 2003)
80	aaptamine	sponge	alkaloid	25	-	(Calcul et al. 2003)
81	isoaaptamine	sponge	alkaloid	6	-	
82	demethyl(oxy)aaptamine	sponge	alkaloid	25	-	
83	purpurealidin B	sponge	alkaloid	10	-	(Tilvi et al. 2004)
84	16-debromoaplysamine-4	sponge	alkaloid	200	-	

85	purpuramine I	sponge	alkaloid	50	-	
86	(1*Z*,4*Z*)-7RH-11-aminogermacra-1(10),4-diene	sponge	terpenoid	-	-	(Satitpatipan et al. 2004)
87	abyssomicin C	bacteria	macrolide	-	4−13	(Riedlinger et al. 2004)
88	24*R*-O-Methylmanoalide	sponge	terpenoid	-	-	(Namikoshi et al. 2004)
89	24*S*-O-Methylmanoalide	sponge	terpenoid	-	-	
90	manoalide-25-acetate	sponge	terpenoid	-	-	
91	laurinterol acetate	aplysia	phenol	-	-	
92	laurinterol	aplysia	phenol	16	-	(Tsukamoto et al. 2005)
93	debromolaurinterol acetate	aplysia	phenol	6	-	
94	debromolaurinterol	aplysia	phenol	10	-	
95	(+)-epoxydon	fungus	polyketide	-	12.5	(Li et al. 2005)
96	(+)-epoxydon monoacetate	fungus	polyketide	-	12.5	
97	gentisyl alc.	fungus	polyketide	-	12.5	
98	3-chlorogentisyl alc	fungus	polyketide	-	50	
99	methylhydroquinone	fungus	polyketide	-	6.2	
100	bis (2,3-dibromo-4, 5-dihydroxybenzyl) ether	red algae	phenol	70	-	(Han et al. 2005)
101	1-hydroxy-1 norresistomycin	bacteria	polyketide	64	-	(Gorajana et al. 2005)
102	refluostatin	bacteria	terpenoid	-	-	(Yao et al. 2006)
103	lasiodiplodin	fungus	macrolide	25	-	(Yang et al. 2006)
104	de-O-methyllasiodiplodin	fungus	macrolide	6.25	-	
105	5-hydroxy-de-O-methyllasiodiplodin	fungus	macrolide	100	-	
106	1-methyl-1,4 dihydroquinoline	bacteria	alkaloid	-	-	(Uzair et al. 2006)
107	BE-43472B	bacteria	polyketide	0.0275−0.0605	0.0605−0.2475	(Socha et al. 2006)
108	BE-43472A	bacteria	polyketide	0.125−0.5	0.25−0.5	
109	bisanthraquinone derivative 3	bacteria	polyketide	4.0−8.0	4.0−80	
110	bisanthraquinone derivative 4	bacteria	polyketide	0.5−＞32*	0.5−＞32	
111	bisanthraquinone derivative 5	bacteria	polyketide	4	4	
112	bisanthraquinone derivative 6	bacteria	polyketide	0.06−0.12	0.06	
113	bisanthraquinone derivative 7	bacteria	polyketide	1.0−2.0	0.125−0.5	
114	bisanthraquinone derivative 8	bacteria	polyketide	0.126	0.126−0.5	

115	3,8-dihydroxy-1-propylanthraquinone-2-carboxylicacid	bacteria	polyketide	-	-	(Poumale et al. 2006)
116	8-hydroxy-3-methoxy-1-propylanthraquinone	bacteria	polyketide	-	-	
117	amacanthin A	sponge	alkaloid	3.12−12.5	6.25−12.5	(Oh et al. 2006; Oh et al. 2005)
118	bromodeoxytopsentin	sponge	alkaloid	50	5-−100	
119	bromotopsentin	sponge	alkaloid	12.5−25	25	
120	hamacanthin A	sponge	alkaloid	3.12	3.12	
121	trans-4,5-dihydro hamacanthin A	sponge	alkaloid	12.5	12.5−25	
122	hamacanthin B	sponge	alkaloid	3.12−6.25	3.12−6.25	
123	6-debromo hamacanthin A	sponge	alkaloid	6.25	6.25−12.5	
124	6-debromohamacanthin B	sponge	alkaloid	50	50	
125	(-)-agelasidine A	sponge	alkaloid	50	-	(Medeiros et al. 2006)
126	asperflavin ribofuranoside	fungus	polyketide	50	50	(Y Li et al. 2006)
127	dehydroxybisdethiobis(methylthio)gliotoxin	fungus	alkaloid	31.2	31.2	(X Li et al. 2006)
128	bisdethiobis(methylthio)gliotoxin	fungus	alkaloid	31.2	31.2	
129	gliotoxin	fungus	alkaloid	1	1	
130	awajanoran	fungus	polyketide	-	-	(Jang et al. 2006)
131	holomycin	bacteria	alkaloid	-	-	(Cui et al. 2006)
132	2-aminophenoxazin-3-one	bacteria	alkaloid	-	-	(Bitzer et al. 2006)
133	2-amino-6-hydroxyphenoxazin-3-one	bacteria	alkaloid	-	-	
134	2-amino-8-benzoyl-6-hydroxyphenoxazin-3-one	bacteria	alkaloid	-	-	
135	hydroxyphenoxazin-3-one	bacteria	alkaloid	-	-	
136	12*R*,13*S*-dihydroxyfumitremorgin C	fungus	alkaloid	125	125	(Zhang et al. 2007)
137	fumitremorgin C	fungus	alkaloid	125	125	
138	brevianamide F	fungus	alkaloid	125	125	
139	hedistin	other invertebrate	peptide	7.5−15	-	(Tasiemski et al. 2007)

140	dehydroxychlorofusarielin B	fungus	terpenoid	62.5	62.5	(Nguyen et al. 2007)
141	fusarielin A	fungus	terpenoid	32.5	32.5−62.5	
142	fusarielin B	fungus	terpenoid	62.5	62.5	
143	macrolactin Q	bacteria	macrolide	0.7	-	(Lu et al. 2007)
144	CgPep33	other	peptide	40	-	(Liu et al. 2007)
145	isoptilocaulina	sponge	alkaloid	170	1.3−85	(Kossuga et al. 2007)
146	demethylaaptamine	sponge	alkaloid	25	-	(Jang et al. 2007)
147	demethyloxyaaptamine	sponge	alkaloid	25	-	
148	6-tribromo-5-methoxymethyl-benzene-1,2-diol	red algae	phenol	2.8	-	(Etahiri et al. 2007)
149	chlorohydroaspyrone A	fungus	polyketide	62.5	125	(Zhang et al. 2008)
150	chlorohydroaspyrone B	fungus	polyketide	62.5	125	
151	macrolactin T	bacteria	macrolide	5.5	-	(Xue et al. 2008)
152	macrolactin B	bacteria	macrolide	4.5	-	
153	tetrahydrobostrycin	fungus	polyketide	-	-	(Xu et al. 2008)
154	1-deoxytetrahydrobostrycin	fungus	polyketide	-	-	
155	zafrin	bacteria	terpenoid	-	60	(Uzair et al. 2008)
156	nigrospoxydon A	fungus	polyketide	64	＞128	(Trisuwan et al. 2008)
157	ZF51(A)	fungus	other	12.5	-	(Tan et al. 2008)
158	lipoxazolidinone A	bacteria	alkaloid	2	2	(Sunga et al. 2008)
159	anhydrofusarubin	fungus	polyketide	12.5	-	(C-L Shao et al. 2008)
160	3-O-methylfusarubin	fungus	polyketide	50	-	(C Shao et al. 2008)
161	fusarubin	fungus	polyketide	12.5	-	
162	lynamicin A	bacteria	alkaloid	1.8	2	(McArthur et al. 2008)
163	lynamicin B	bacteria	alkaloid	0.8	1	
164	lynamicin C	bacteria	alkaloid	1.1	1.5	
165	lynamicin D	bacteria	alkaloid	3	3	
166	lynamicin E	bacteria	alkaloid	＞20	12	

167	macrolactin S	bacteria	macrolide	0.7	-	(Han et al. 2008; Lu et al. 2008)
168	macrolactin A	bacteria	macrolide	6.3	-	
169	cyclo-(L-Pro-L-Phe)	bacteria	peptide	-	-	(Li et al. 2008)
170	deoxycymodienol	othersea weed	biphenyl	-	32-	(Kontiza et al. 2008)
171	cymodienol	othersea weed	biphenyl	-	64	
172	isocymodiene	othersea weed	biphenyl	-	32−64	
173	nodosol	othersea weed	terpenoid	-	16	
174	ascochytatin	fungus	phenol	-	-	(Kanoh, Okada, et al. 2008)
175	sulfoalkylresorcinol	fungus	phenol	-	12.5	(Kanoh, Adachi, et al. 2008)
176	(-)-marinopyrrole A	bacteria	alkaloid	-	0.61 μM	(Hughes et al. 2008)
177	(-)-marinopyrrole B	bacteria	alkaloid	-	1.1 μM	
178	asphodelin	bacteria	polyketide	3	-	(El-Gendy et al. 2008)
179	4,7-bichrysophanol	bacteria	polyketide	1.6	-	
180	justicidin B	bacteria	other	1	-	
181	1,1-dichloro 4-ethyl-5-(4-nitro-phenyl)-hexan-2-one	bacteria	other	0.1	-	
182	palmitoleic acid	brown algae	aliphatic acid	-	-	(Desbois et al. 2008)
183	HTA	brown algae	aliphatic acid	-	-	
184	3,4,5-trimethyl-1,2-resorcinol	fungus	phenol	-	-	(Cui et al. 2008)
185	decarboxydihydrocitrinone	fungus	polyketide	-	-	
186	aeroplysinin-1	sponge	phenol	-	-	(Abou-Shoer et al. 2008)
187	clavatol	fungus	polyketide	62.5	31.0−62.5	(Yang et al. 2009)
188	2,3,6-tribromo-4,5-dihydroxybenzyl ethyl ether	red algae	phenol	102	-	(Xu et al. 2009)

189	2,3,6-tribromo-4,5-dihydroxybenzyl methyl ether	red algae	phenol	50	-	
190	*Z*-dihydrorhodophytin	red algae	other	-	-	(Vairappan et al. 2009)
191	nigrosporapyrone A	fungus	polyketide	128	-	(Trisuwan et al. 2009)
192	echinomycin	bacteria	peptide	0.011	0.033	(Socha et al. 2009)
193	FD-991	bacteria	peptide	1	2	
194	2-(2’,4’-Dibromophenoxy)-4,6-dibromophenol	sponge	phenol	0.117−0.313	0.313−0.625	(Shridhar et al. 2009)
195	ethylparaben	bacteria	other	＞100	-	(Quévrain et al. 2009)
196	butylparaben	bacteria	other	25–50	-	
197	octylparaben	bacteria	other	25–50	-	
198	decylparaben	bacteria	other	0.78–1.56	-	
199	hydroxydecylparaben	bacteria	other	25–50	-	
200	methyldecylparaben	bacteria	other	12.5–25	-	
201	hydroxymethyldecylparaben	bacteria	other	12.5–25	-	
202	dodecylparaben	bacteria	other	25–50	-	
203	dodec-5-enylparaben	bacteria	other	6.25–12.5	-	
204	alternaramide	fungus	peptide	-	-	(Kim et al. 2009)
205	motualevic acid A	sponge	alkaloid	10.9± 4.0	9.3 ± 2.7	(Keffer et al. 2009)
206	motualevic acid F	sponge	alkaloid	1.2± 0.3	3.9 ± 1.0	
207	MC21-B	bacteria	biphenyl	-	1−4	(Isnansetyo et al. 2009)
208	eicosapentaenoic acid	brown algae	other	-	-	(Desbois et al. 2009)
209	aureobasidin	fungus	aliphatic acid	-	-	(Abdel-Lateff et al. 2009)
210	(3*R*,5*S*)-3,5-dihydroxydecanoic acid	fungus	aliphatic acid	-	-	
211	(5*R*,3*Z*)-5-hydroxydec-3-enoic acid	fungus	aliphatic acid	-	-	
212	agelasine-D	sponge	alkaloid	1.25	-	(Xu et al. 2010)
213	cyclo(*S*-Pro-*S*-Phe)	bacteria	peptide	0.25−0.5	-	(Wang et al. 2010)
214	cyclo(*R*-Pro-*S*-Phe)	bacteria	peptide	-	-	
215	ioniol II	red algae	terpenoid	-	32−64	(Smyrnioto

						poulos et al. 2010)
216	bromotetrasphaerol	red algae	terpenoid	-	32−128	
217	coronopifoliol	red algae	terpenoid	64	16−32	
218	1-acetyl-β-carboline	bacteria	alkaloid	-	-	(Shin et al. 2010)
219	3,9-dimethyldibenzo[*b*,*d*]furan-1,7-diol	fungus	polyketide	-	-	(Roue et al. 2010)
220	butyrolactone I	fungus	polyketide	-	-	
221	bromomethylchlamydosporol A	fungus	polyketide	15.6	15.6	(Rateb et al. 2010)
222	bromomethylchlamydosporol B	fungus	polyketide	15.6	15.6	
223	chlamydosporol	fungus	polyketide	31.5	31.5− 62.5	
224	(-)-marinopyrroles A	bacteria	alkaloid	-	0.31	(Nenkep et al. 2010)
225	(-)-marinopyrroles B	bacteria	alkaloid	-	0.63	
226	(-)-marinopyrroles C	bacteria	alkaloid	-	0.16	
227	(±)-marinopyrrole F	bacteria	alkaloid	-	3.1	
228	2,3,5,7-tetrabromobenzofuro[3,2-b] pyrrole	bacteria	alkaloid	-	-	(Hughes et al. 2010)
229	4,4′,6-tribromo-2,2′-biphenol	bacteria	biphenyl	-	-	
230	bromophene	bacteria	biphenyl	-	-	
231	pentabromopseudilin	bacteria	alkaloid	-	-	
232	crossbyanol B	bacteria	polyketide	-	2.0−3.9	(Feher et al. 2010)
233	spongotine	coral	alkaloid	12.5	12.5	(Zoraghi et al. 2011)
234	cis-3,4-dihyrohyrohamacanthin B	coral	alkaloid	12.5	12.5	
235	bro modeoxytopsentin	coral	alkaloid	6.25	6.25	
236	fascioquinol A	sponge	terpenoid	2.4−2.5	-	(Zhang et al. 2011)
237	fascioquinol B	sponge	terpenoid	0.95−1	-	
238	flavuside A	fungus	other	15.6	31.2	(Yang et al. 2011)
239	flavuside B	fungus	other	15.6	31.2	
240	phomaligol A	fungus	polyketide	31.2	62.5	
241	fijimycin A	bacteria	peptide	-	4−32	(Sun et al. 2011)
242	fijimycin B	bacteria	peptide	-	＞32	

243	fijimycin C	bacteria	peptide	-	8−32	
244	etamycin A	bacteria	peptide	-	4−16	
245	lobophorin E	bacteria	macrolide	32	-	(Niu et al. 2011)
246	lobophorin F	bacteria	macrolide	8	-	
247	deoxynyboquinone	bacteria	alkaloid	1	-	(Li et al. 2011)
248	pseudonocardian A	bacteria	alkaloid	2	-	
249	pseudonocardian B	bacteria	alkaloid	4	-	
250	conidiogenone B	fungus	terpenoid	-	8	(Gao et al. 2011)
251	(-)-trypethelone	fungus	polyketide	-	-	(Elsebai, Natesan, et al. 2011)
252	conioscleroderolide	fungus	polyketide	7.8	-	(Elsebai, Kehraus, et al. 2011)
253	(−) scleroderolide	fungus	polyketide	7.8	-	
254	siphonellinol A	sponge	terpenoid	-	-	(Aqil et al. 2011)
255	(+)-sydonic acid	fungus	terpenoid	2.6	-	(Zheng et al. 2012)
256	expansol G	fungus	terpenoid	6.4	-	
257	(+)-sydowic acid	fungus	terpenoid	5.4	-	
258	sorbicillin	fungus	polyketide	-	-	(Zhao et al. 2012)
259	2',3'-dihydrosorbicillin	fungus	polyketide	-	-	
260	2,3-dihydro-7-hydroxy-6,8-dimethyl-2-[(E)-prop-l-enyl]chromen-4-one	fungus	polyketide	-	-	
261	dibutylphthalate	fungus	polyketide	-	-	
262	6,8-di-O-methylnidurufin	fungus	polyketide	-	-	(Y Zhang et al. 2012)
263	rabelomycin	bacteria	polyketide	1	-	(W Zhang et al. 2012)
264	phenanthroviridone	bacteria	polyketide	0.25	-	
265	fluostatin C	bacteria	polyketide	16	-	
266	fluostatin F	bacteria	polyketide	32	-	
267	5-hydroxymethyl-furoic acid	fungus	other	-	-	(L Zhang et al. 2012)
268	isochromophilone VIII	fungus	polyketide	-	-	

269	*R*-3-hydroxyundecanoic acid methylester-3-O-α-L-rhamnopyranoside	mangrove	other	-	-	(Zeng et al. 2012)
270	ageloxime B	sponge	alkaloid	10	20	(Yang et al. 2012)
271	porric acid D	fungus	biphenyl	100	-	(Xu et al. 2012)
272	altenusin	fungus	biphenyl	25	-	
273	fradimycin A	bacteria	polyketide	6	-	(Xin et al. 2012)
274	fradimycin B	bacteria	polyketide	2	-	
275	MK844-mF10	bacteria	polyketide	4	-	
276	peptidolipin B	Pyrosomella verticilliata	peptide	64	64	(Wyche et al. 2012)
277	peptidolipin E	Pyrosomella verticilliata	peptide	64	64	
278	xanthocillin X	fungus	alkaloid	1*	-	(Wyche et al. 2012)
279	meleagrin	fungus	alkaloid	64	-	(Hamed et al. 2021; Wyche et al. 2012)
280	brevianamide M	fungus	alkaloid	-	-	(Miao et al. 2012)
281	6,8-di-O-methylnidurufin	fungus	polyketide	-	-	
282	6,8-di-O-methylaverufin	fungus	polyketide	-	-	
283	guignardone I	fungus	terpenoid	-	-	(Mei et al. 2012)
284	guignardone B	fungus	terpenoid	-	-	
285	albican-11,14-diol	fungus	terpenoid	-	-	(X-H Liu et al. 2012)
286	citreamicin θ A	bacteria	polyketide	1	0.25	(L-L Liu et al. 2012)
287	citreamicin θ B	bacteria	polyketide	1*	0.25	
288	citreaglycon A	bacteria	polyketide	16	8	
289	dehydrocitreaglycon A	bacteria	polyketide	16	8	
290	aspergillumarin A	fungus	polyketide	50	-	(Li et al. 2012)
291	aspergillumarin B	fungus	polyketide	50	-	

292	5- bromotoluhydroquinone	fungus	phenol	-	6.2	(Leutou et al. 2012)
293	4-O-methyltoluhydroquinone	fungus	phenol	-	12.5	
294	toluhydroquinone	fungus	phenol	-	6.2	
295	gentisyl alcohol	fungus	phenol	-	12.5	
296	trichodermaquinone	fungus	polyketide	-	8	(Khamthong, Rukachaisirikul, Tadpetch, et al. 2012)
297	emodin	fungus	polyketide	-	4	
298	penicillanthranins A	fungus	polyketide	16	16	(Khamthong, Rukachaisirikul, Phongpaichit, et al. 2012)
299	antimycin B2	bacteria	polyketide	32	-	(Han et al. 2012)
300	2-nonylquinoxalin-6-ol	bacteria	alkaloid	-	2−3	(Gnanamani et al. 2012)
301	kojic acid	fungus	polyketide	-	-	(Du, Li, Li, & Wang 2012)
302	N-[2-(4-p-hydroxyphenol) ethyl] acetamide	fungus	alkaloid	-	-	
303	cristatumin A	fungus	alkaloid	64	-	(Du, Li, Li, Shang, et al. 2012)
304	tardioxopiperazine A	fungus	alkaloid	8	-	
305	12-hydroxy isolaurene	red algae	terpenoid	52	-	(Alarif et al. 2012)
306	8,11-dihydro-12-hydroxy isolaurene	red algae	terpenoid	31	-	
307	isolauraldehyde	red algae	terpenoid	27	-	
308	aspochalasin D	fungus	polyketide	4.01	-	(Zheng et al. 2013)
309	3-((6-methylpyrazin-2-yl)methyl)-1*H*-indole	bacteria	alkaloid	96	-	(Yang et al. 2013)
310	4a-dechloronapyradiomycin A1	bacteria	polyketide	4	-	(Z Wu et al. 2013)

311	3-dechloro-3-brominapyradiomycin A1	bacteria	polyketide	0.5	-	
312	napyradiomycin A1	bacteria	polyketide	1	-	
313	18-oxonapyradiomycin A1	bacteria	polyketide	32	-	
314	napyradiomycin B1	bacteria	polyketide	1	-	
315	napyradiomycin B3	bacteria	polyketide	0.5	-	
316	naphthomevalin	bacteria	polyketide	1	-	
317	11′,12′-dehydroelaiophylin	bacteria	macrolide	2	2−＞64	(C Wu et al. 2013)
318	11,11′-O-dimethyl-14′-deethyl-14′-methylelaiophylin	bacteria	macrolide	32	16−32	
319	elaiophylin	bacteria	macrolide	1	1−2	
320	11-O-methylelaiophylin	bacteria	macrolide	2	1−2	
321	11,11′-O-dimethylelaiophylin	bacteria	macrolide	16	4−16	
322	efomycin G	bacteria	macrolide	2	22	
323	penicitrinol J	fungus	polyketide	-	-	(Sabdaningsih et al. 2020; Wang et al. 2013)
324	penicitrinol K	fungus	polyketide	-	-	(Wang et al. 2013)
325	gageomacrolactin 1	bacteria	macrolide	0.0125	-	(Tareq et al. 2013)
326	gageomacrolactin 2	bacteria	macrolide	0.063	-	
327	gageomacrolactin 3	bacteria	macrolide	0.056	-	
328	yicathin C	fungus	polyketide	-	-	(Sun et al. 2013)
329	marfuraquinocin A	bacteria	terpenoid	8	＞128	(Song et al. 2013)
330	marfuraquinocin B	bacteria	terpenoid	64	-	
331	marfuraquinocin C	bacteria	terpenoid	8	-	
332	marfuraquinocin D	bacteria	terpenoid	8	-	
333	psammaplysin F	sponge	alkaloid	42.8	40−80	(Ramsey et al. 2013)
334	psammaplysin H	sponge	alkaloid	20.4−45.0	40−80	
335	chrysophanol	fungus	polyketide	-	-	(Maarisit et al. 2013)
336	rubelin A	fungus	polyketide	-	-	
337	14-acetoxyrubellin A	fungus	polyketide	-	-	
338	14-acetoxyrubellin C	fungus	polyketide	-	-	
339	16-deethylindanomycin	bacteria	terpenoid	4.0−8.0		
340	iso-16-deethylindanomycin	bacteria	terpenoid	-	-	

341	16-deethylindanomy cin methyl ester	bacteria	terpenoid	-	-	(Lian et al. 2013)
342	iso-16-deethylindanomycin methyl ester	bacteria	terpenoid	-	-	
343	cyclo-(L-Pro-L-Tyr)	fungus	peptide	32	-	(Li et al. 2013)
344	cyclo-(L-Pro-L-Leu)	fungus	peptide	64	-	
345	1-acetyl--corboline	bacteria	alkaloid	16−32	32−128	(Lee et al. 2013)
346	epi-ilimaquinone	sponge	terpenoid	31.25	62.5	(Kumar et al. 2013)
347	zamamiphidin A	sponge	alkaloid	32	-	(Kubota et al. 2013)
348	1-(3-Bromo-5-methylphenyl)-1*H*-indole	bacteria	alkaloid	1.56	-	(Gunasekaran et al. 2013)
349	helalomycin-1	bacteria	terpenoid	1.5	1.3	(El-Sayed et al. 2013)
350	2-hydroxy-5-((6-hydroxy-4-oxo-4H-pyran-2-yl)methyl)-2-propylchroman-4-one	bacteria	polyketide	-	2	(Djinni et al. 2013)
351	napyradiomycin 1	bacteria	terpenoid	-	16	(Cheng et al. 2013)
352	napyradiomycin 2	bacteria	terpenoid	-	64	
353	napyradiomycin 3	bacteria	terpenoid	-	＞64	
354	napyradiomycin 4	bacteria	terpenoid	-	＞64	
355	napyradiomycin 5	bacteria	terpenoid	-	＞64	
356	napyradiomycin 6	bacteria	terpenoid	-	＞64	
357	napyradiomycin B2	bacteria	terpenoid	-	16-64	
358	napyradiomycin B4	bacteria	terpenoid	-	32	
359	(+)-butyrolactone I	fungus	polyketide	0.31	-	(Z-q Chen et al. 2013)
360	butyrolactone IV	fungus	polyketide	0.31	-	
361	butyrolactone II	fungus	polyketide	0.31	-	
362	butyrolactone III	fungus	polyketide	3.12	-	
363	methyl 2,4-dihydroxy-3,5,6-trimethylbenzoate	fungus	polyketide	0.31	-	
364	3,4-bis(4-hydroxyphenyl)-1-phenethyl-1H-pyrrole-2,5-dione	bacteria	alkaloid	500	-	(Y Chen et al. 2013)
365	solwaric acid A	echinoderm	aliphatic acid	64	32	(Ellis et al. 2014)
366	solwaric acid B	echinode	aliphatic acid	-	-	

		rm				
367	microluside A	sponge	polyketide	12.4	-	(Eltamany et al. 2014)
368	dysidinoid A	sponge	terpenoid	-	8.0	(Jiao et al. 2014)
369	diaporthalasin 1	sponge	alkaloid	2	2	(Khamthong et al. 2014)
370	ianthelliformisamine A	sponge	alkaloid	5.74	-	(Khan et al. 2014)
371	ianthelliformisamine B	sponge	alkaloid	66.71	-	
372	didemnaketal D	echinoderm	polyketide	-	-	(Mohamed et al. 2014)
373	spiromastixone C	fungus	polyketide	11.59	23.17	(Niu et al. 2014)
374	spiromastixone E	fungus	polyketide	25.35	12.67	
375	spiromastixone F	fungus	polyketide	1.72	1.72	
376	spiromastixone G	fungus	polyketide	3.55	1.78	
377	spiromastixone H	fungus	polyketide	1.72	1.72	
378	spiromastixone I	fungus	polyketide	7.42	7.42	
379	spiromastixone J	fungus	polyketide	1.91	0.96	
380	chevalone E	sponge	terpenoid	-	-	(Prompanya et al. 2014)
381	rifamycin W	sponge	other	-	15.62	(Singh et al. 2014)
382	5-methoxydihydrosterigmatocystin	sponge	polyketide	12.5	-	(F Song et al. 2014)
383	desotamide A	bacteria	peptide	16	32	(Y Song et al. 2014)
384	desotamide B	bacteria	peptide	16	32	
385	indole-diterpenoid	fungus	terpenoid	9.95	-	(Sun et al. 2014)
386	gageostatin A	bacteria	peptide	16	-	(Tareq et al. 2014)
387	gageostatin B	bacteria	peptide	8−16	-	
388	gageostatin C	bacteria	peptide	64	-	
389	stachyin B	fungus	steroid	-	1.32	(Yu et al. 2014)
390	stachybocin B	fungus	steroid	-	3.6	
391	stachybocin A	fungus	steroid	-	3.27	
392	ilicicolin B	fungus	terpenoid	-	0.26	
393	penicimonoterpene (±)-1	fungus	terpenoid	8	-	(Zhao et al.

						2014)
394	sarcotrocheliol acetate	coral	polyketide	-	-	(Al-Footy et al. 2015)
395	sarcotrocheliol	coral	polyketide	-	-	
396	cembrene-C	coral	terpenoid	-	10 ± 2.0	
397	sarcophine	coral	other	10 ± 1.0	10 ± 0.5	
398	palustrol	coral	other	11 ± 1.0	8 ± 1.4	
399	hypofuran A	sponge	other	32	-	(Ding et al. 2015)
400	N-isobutyl-2-phenylacetamide	sponge	other	32	-	
401	citrantifidiol	sponge	other	32	-	
402	penicyclone A	fungus	polyketide	0.3	-	(Guo et al. 2015)
403	penicyclone B	fungus	polyketide	1	-	
404	penicyclone C	fungus	polyketide	0.9	-	
405	penicyclone D	fungus	polyketide	0.8	-	
406	penicyclone E	fungus	polyketide	0.5	-	
407	methyl (*Z*)-3-(3,4-dihydroxyphenyl)-2-formamidoacrylate	seaweed	phenol	2	-	(Li et al. 2015)
408	adametizine A	fungus	alkaloid	8	-	(Liu et al. 2015)
409	adametizine B	fungus	alkaloid	64	-	
410	spiromastol 1	fungus	phenol	0.5	-	(Niu et al. 2015)
411	spiromastol 2	fungus	phenol	4	-	
412	spiromastol 3	fungus	phenol	0.25	-	
413	spiromastol 9	fungus	phenol	8	-	
414	spiromastol 10	fungus	phenol	32	-	
415	spiromastol 11	fungus	phenol	32	-	
416	sphaerodactylomelol	red algae	terpenoid	-	-	(Rodrigues et al. 2015)
417	bromosphaerol	red algae	terpenoid	-	-	
418	12R-hydroxybromosphaerol	red algae	terpenoid	-	-	
419	DG-1	sponge	phenol	1	0.1ng/mL	(Sun et al. 2015)
420	DG-2	sponge	phenol	-	0.1ng/mL	
421	D-1	sponge	phenol	-	1	
422	halichonadin O	sponge	terpenoid	8	-	(Tanaka et al. 2015)
423	ergostere-7, 22-diene-3β, 5α, 6β-triol	fungus	steroid	-	-	(Tao et al. 2015)
424	ergostere-5, 7, 22-triene-3-β-ol	fungus	steroid	-	-	

425	p-hydroxyacetophenone	fungus	steroid	-	-	
426	ieodoglucomide C	bacteria	other	0.018	-	(Tareq et al. 2015)
427	ieodoglycolipid	bacteria	other	-	-	
428	spiculisporic acid F	echinoderm	polyketide	-	-	(Wang et al. 2015)
429	spiculisporic acid G	echinoderm	polyketide	-	-	
430	talaromycesone A	fungus	polyketide	-	3	(Wu, Ohlendorf, et al. 2015)
431	talaromycesone B	fungus	polyketide	-	10.4	
432	AS-186c	fungus	polyketide	-	1.29	
433	lindgomycin	sponge	polyketide	1.29	2.44	(Wu, Wiese, et al. 2015)
434	ascosetin	sponge	polyketide	1.33	1.47	
435	quinomycin G	sponge	alkaloid	32	32	(Zhen et al. 2015)
436	cembrene A	coral	terpenoid	30	-	(Al-Footy et al. 2016)
437	alismoxide	coral	other	30	-	
438	nephalsterol C	coral	steroid	30	-	
439	alismol-1	coral	terpenoid	15	-	
440	vineomycin A1	bacteria	polyketide	4	-	(Hu et al. 2016)
441	proximicin B	fungus	alkaloid	-	3.125	(Fang et al. 2020; Huang et al. 2016)
442	glycerol 1-hydroxy-2,5-dimethyl benzoate	fungus	phenol	-	12.5	(Huang et al. 2016)
443	citrinovirin	brown algae	terpenoid	12.4	-	(X-R Liang et al. 2016)
444	N-acetyl-N-demethylmayamycin	bacteria	polyketide	-	9.8	(Y Liang et al. 2016)
445	penicilloside B	fungus	other	-	-	(Murshid et al. 2016)
446	oxysporizoline	fungus	alkaloid	-	6.25	(Nenkep et al. 2016)
447	langcoquinone A	sponge	terpenoid	4.46	-	(Nguyen et al. 2016)
448	langcoquinone B	sponge	terpenoid	5.39	-	
449	engyodontochone A	fungus	polyketide	-	0.17±0.02µ	(Wu et al.

					M	2016)
450	JBIR-97/98	fungus	polyketide	-	0.1	
451	JBIR-99	fungus	polyketide	-	0.15	
452	engyodontochone B	fungus	polyketide	-	0.14	
453	engyodontochone C	fungus	polyketide	-	0.24	
454	engyodontochone D	fungus	polyketide	-	4.25	
455	engyodontochone E	fungus	polyketide	-	1.99	
456	dysidphenol A	sponge	terpenoid	100	-	(Zhang et al. 2016)
457	dysidphenol C	sponge	terpenoid	50	-	
458	smenospongimine	sponge	terpenoid	3.125	-	
459	smenospongine	sponge	terpenoid	3.125	-	
460	smenospongorine	sponge	terpenoid	3.125	-	
461	smenospongiarine	sponge	terpenoid	6.25	-	
462	smenospongidine	sponge	terpenoid	3.125	-	
463	penicilazaphilone B	fungus	terpenoid	250	-	(Zhou et al. 2016)
464	penicilazaphilone C	fungus	alkaloid	31.25	-	
465	trocheliane	coral	terpenoid	2.15	-	(Zubair et al. 2016)
466	marinocyanin A	bacteria	alkaloid	0.97	-	(Asolkar et al. 2017)
467	marinocyanin B	bacteria	alkaloid	11.64	-	
468	marinocyanin C	bacteria	alkaloid	15.63	-	
469	marinocyanin D	bacteria	alkaloid	15.64	-	
470	marinocyanin E	bacteria	alkaloid	15.64	-	
471	marinocyanin F	bacteria	alkaloid	15.64	-	
472	2-bromo-1-hydroxyphenazine	bacteria	alkaloid	15.94	-	
473	lavanducyanin	bacteria	alkaloid	0.97	-	
474	lobophorin K	coral	macrolide	40-80	>160	(Brana et al. 2017)
475	penicilone B	fungus	polyketide	3.13	3.13	(Chen et al. 2017)
476	penicilone C	fungus	polyketide	6.25	6.25	
477	penicilone D	fungus	polyketide	6.25	6.25	
478	rhodozepinone-1	sponge	alkaloid	-	-	(Elsayed et al. 2017)
479	(+)-agelasine B	sponge	alkaloid	2	1	(Hong et al. 2017)
480	agelasine D	sponge	alkaloid	1	8	
481	chalcomycin	bacteria	macrolide	4	-	(Jiang et al. 2017)
482	dihydrochalcomycin	bacteria	macrolide	32	-	

483	DEHP	bacteria	other	-	-	(Kalinovskaya et al. 2017)
484	cyclo-(L-Val-D-Pro)	bacteria	peptide	-	-	
485	cyclo-(L-Phe-D-Pro)	bacteria	peptide	-	-	
486	verruculide B2	fungus	terpenoid	32	-	(Kong et al. 2017)
487	5, 6-dihydro-1, 8-dihydroxy-3-methylbenzo [a] anthracene 7, 12-quinone	bacteria	polyketide	-	-	(Kurata et al. 2017)
488	heterocornol A	sponge	polyketide	25	-	(Lei, Lin, Han, Ma, Dong, et al. 2017)
489	heterocornol B	sponge	polyketide	25	-	
490	heterocornol C	sponge	polyketide	50	-	
491	heterocornol F	sponge	polyketide	50	-	
492	heterocornol I	sponge	polyketide	50	-	
493	heterocornol J	sponge	polyketide	25	-	
494	heterocornol K	sponge	polyketide	100	-	
495	heterocornol L	sponge	polyketide	50	-	
496	heterocornol Q	sponge	polyketide	50	-	
497	pestaloisocoumarin A	sponge	polyketide	25	-	(Lei, Lin, Han, Ma, Ma, et al. 2017)
498	pestaloisocoumarin B	sponge	polyketide	-	-	
499	gamahorin	sponge	polyketide	100	-	
500	pestalachloride B	sponge	other	3	-	
501	pestalachloride E	sponge	polyketide	25	-	
502	pestalalactone atropisomers	sponge	polyketide	50	-	
503	tulongicin A	sponge	alkaloid	1.2	-	(H-B Liu, G Lauro, RD O'Connor, et al. 2017)
504	dihydrospongotine C	sponge	alkaloid	3.7	-	
505	spongotine C	sponge	alkaloid	1.1	-	
506	dibromodeoxytopsentin	sponge	alkaloid	1.1	-	
507	altersolanol B	fungus	polyketide	-	32	(P Liu et al. 2017)
508	altersolanol C	fungus	polyketide	-	32	
509	auxarthrol C	fungus	polyketide	-	32	
510	alterporriol E	fungus	polyketide	-	32	

511	altersolanol A	fungus	other	-	0.5	
512	microcionamides C	sponge	peptide	5.6	-	(Mokhlesi et al. 2017)
513	microcionamide A.	sponge	peptide	5.5	-	
514	langcoquinone C	sponge	terpenoid	5	-	(Nguyen et al. 2017)
515	meroterpenoid	sponge	terpenoid	9.98	-	
516	pestalotionol	fungus	phenol	8	-	(Pan et al. 2017)
517	Neo-actinomycin A	bacteria	peptide	16	16	(Q Wang et al. 2017)
518	actinomycins D	bacteria	peptide	0.25	0.25	
519	actinomycin X_2	bacteria	peptide	0.25	0.25	
520	ethyl 3-methoxy-3,5-dimethoxy-2-propionylphenylacetate	fungus	other	-	7.8	(W Wang et al. 2017)
521	eleganstone A	sponge	terpenoid	64	-	(Yu et al. 2017)
522	precursor	sponge	terpenoid	32	-	
523	diterpene	sponge	terpenoid	64	-	
524	streptopertusacin A	sponge	alkaloid	-	40	
525	21,22-en-bafilomycin D	bacteria	other	-	12.5	(Zhang et al. 2017)
526	21,22-en-9-hydroxybafilomycin D	bacteria	other	-	-	
527	poecillasirin A	sponge	alkaloid	-	-	(H-B Liu, G Lauro, R O'Connor, et al. 2017)
528	poecillasirinol	sponge	alkaloid	-	-	
529	(*E*)-4-oxonon-2-enoic acid	fungus	other	16	-	(An et al. 2018)
530	helvolic acid	fungus	steroid	2	-	
531	15'-[(6-Bromo-1H-indol-3-yl)methyl]-3'-methylimidazolidine-2',4'-dione	sponge	alkaloid	-	-	(Bagalagel et al. 2018)
532	arthpyrone H	fungus	alkaloid	3.86	-	(Bao et al. 2018)
533	arthpyrone F	fungus	alkaloid	3.84	-	
534	arthpyrone I	fungus	alkaloid	6.49	-	
535	arthpyrone L	fungus	alkaloid	0.71	-	
536	anaephene B	bacteria	phenol	6.1	-	(Brumley et

						al. 2018)
537	streptopyrazinone A	bacteria	alkaloid	-	58	(Chen et al. 2018)
538	streptopyrazinone B	bacteria	alkaloid	-	60	
539	streptopyrazinone C	bacteria	alkaloid	-	62	
540	streptopyrazinone D	bacteria	alkaloid	-	65	
541	N-acetyl-L-isoleucine-L-leucinamide	bacteria	alkaloid	-	65	
542	peniginsengin C	fungus	terpenoid	8	-	(Cheng et al. 2018)
543	peniginsengin D	fungus	terpenoid	16	-	
544	peniginsengin E	fungus	terpenoid	32	-	
545	*R*-(+)-N-[15-methyl-3-(12-methyltridecanoyloxy)-hexadecanoyl]glycine	bacteria	other	-	58	(Chianese et al. 2018)
546	comp.4	bacteria	alkaloid	-	22	
547	comp.5	bacteria	alkaloid	-	93	
548	asperitaconic acid A	fungus	aliphatic acid	16	-	(El-Gendy et al. 2018)
549	asperitaconic acid B	fungus	aliphatic acid	32	-	
550	asperitaconic acid C	fungus	aliphatic acid	32	-	
551	cryptophomic acid	fungus	other	4.16	-	(Elsebai et al. 2018)
552	cryptodiol	fungus	other	3.56	-	
553	cryptotriol	fungus	other	1.7	-	
554	asperphenone A	fungus	other	21.27	-	(Imhoff et al. 2018)
555	asperphenone B	fungus	other	9.7	-	
556	emerimicin IV	fungus	peptide	25	100	(Inprasit et al. 2018)
557	coquinones D	sponge	terpenoid	11.18	-	(W Jiang et al. 2018)
558	strepoxepinmycin D	bacteria	polyketide	-	3	(Y-J Jiang et al. 2018)
559	strepoxepinmycin A	bacteria	polyketide	-	12	
560	strepoxepinmycin B	bacteria	polyketide	-	15	
561	strepoxepinmycin C	bacteria	polyketide	-	6	
562	medermycin	bacteria	polyketide	-	0.25	
563	actinomycin D2	sponge	peptide	-	0.25	(Kumla et al. 2018)
564	actinomycin D3	sponge	peptide	-	0.5	
565	actinomycin D4	sponge	peptide	-	0.25	
566	actinomycin D	sponge	peptide	-	0.25	

567	actinomycin D1	sponge	terpenoid	-	0.125	
568	(＋)-sclerodione	fungus	polyketide	-	23	(Li et al. 2018)
569	(＋)-scleroderolide	fungus	polyketide	-	7	
570	fumigatosides E	fungus	alkaloid	6.25 ± 0.13	-	(Moe et al. 2018)
571	fumiquinazoline C	fungus	alkaloid	1.565 ± 0.04	-	
572	fumiquinazoline G	fungus	alkaloid	12.5 ± 0.33	-	
573	alterlactone	sponge	polyketide	31.25	-	(Pang et al. 2018)
574	phocoenamicin B	bacteria	macrolide	8–16	-	(Sletta et al. 2018)
575	phocoenamicin C	bacteria	macrolide	32–64	-	
576	phocoenamicin	bacteria	macrolide	4-8	-	
577	pyrrospirone C	fungus	alkaloid	-	4	(Song et al. 2018)
578	pyrrospirone D	fungus	alkaloid	-	12	
579	pyrrospirone E	fungus	alkaloid	-	10	
580	pyrrospirone H	fungus	alkaloid	-	19	
581	pyrrospirone I	fungus	alkaloid	-	4	
582	pyrrospirone F	fungus	alkaloid	-	2	
583	penicipyrrodiether A	fungus	alkaloid	-	5.0	(J Wang et al. 2018)
584	chaephilone C	fungus	other	-	7.6 ± 0.5	(W Wang et al. 2018)
585	chaetoviridide B	fungus	other	-	7.8 ± 0.8	
586	chaetoviridide C	fungus	other	-	7.6 ± 0.4	
587	bacicyclin	bacteria	peptide	7.2	-	(Wiese et al. 2018)
588	nocardiopsistin A	bacteria	polyketide	-	12.5	(Xu et al. 2018)
589	nocardiopsistin C	bacteria	polyketide	-	12.5	
590	nocardiopsistin B	bacteria	polyketide	-	3.12	
591	diorcinol K	fungus	phenol	3.125	3.125	(Zhang et al. 2018)
592	diorcinol D,	fungus	phenol	6.25	6.25	
593	diorcinol I	fungus	phenol	6.25	6.25	
594	arabomycin	bacteria	polykeide	-	-	(Ahmad et al. 2019)
595	indolepyrazine A	bacteria	alkaloid	-	12	(Anjum et al. 2019)
596	indolepyrazine B	bacteria	alkaloid	-	12	

597	1,1′-dioxine-2,2′-dipropionic acid	fungus	other	25	-	(Bai et al. 2019)
598	citrinin H1	fungus	polyketide	6.25	-	
599	ladosporol E	fungus	polyketide	1.25	-	
600	cladosporol C	fungus	polyketide	6.25	-	
601	2-methyl acetate -3, 5, 6-trimethylpyrazine	fungus	alkaloid	12.5	-	
602	desertomycin G	other（seaweed）	macrolide	4	-	(Brana et al. 2019)
603	2, 2-bis (6-bromo-3-indolyl) ethylamine	sponge	alkaloid	8	-	(Campana et al. 2019)
604	3-hydroxy-2-methylpyridine	bacteria	alkaloid	256	-	(DT Cao et al. 2019)
605	4,5-dihydroxy-7-methylphthalide	bacteria	polyketide	128	-	(D-T Cao et al. 2019)
606	2’-deoxythymidine	bacteria	alkaloid	32	-	
607	2′-deoxyuridine	bacteria	alkaloid	32	-	
608	(2-(hydroxymethyl)-3-(2-(hydroxymethyl)-3-methylaziridin-1-yl) (2-hydroxyphenyl) methanone	sponge	alkaloid	3.4	-	(M-H Chen et al. 2019)
609	(+)-tersone E	fungus	alkaloid	31.2	-	(S-C Chen et al. 2019)
610	(+) ent-citridone A	fungus	alkaloid	31.5	-	
611	salinaphthoquinone B	fungus	polyketide	31	62	(da Silva et al. 2019)
612	nipyrone A	fungus	polyketide	64	128	(Ding et al. 2019)
613	nipyrone B	fungus	polyketide	64	128	
614	nipyronesC	fungus	polyketide	8	128	
615	germicidin C	fungus	polyketide	64	128	
616	5-bromotrisindoline	sponge	alkaloid	16	-	(El-Hawary et al. 2019)
617	6-bromotrisindoline	sponge	alkaloid	8	-	
618	2,3,5-tribromo-6-(3′,5′-dibromophenoxy)phenol	sponge	phenol	-	-	(Hanif et al. 2019)
619	versicolorin B	fungus	polyketide	6.25	12.5	(Hu et al. 2019)
620	averufin	fungus	polyketide	6.25	25	
621	ala-geninthiocin	bacteria	peptide	15	-	(Iniyan et al. 2019)
622	peyssonnoside A	red algae	terpenoid	-	16.7±0.3	(Khatri

						Chhetri et al. 2019)
623	3-bromo-2- (2',4'-dibromophenoxy)-6-iodophenol	sponge	phenol	3.4	-	(Ki et al. 2019)
624	2-(2',4'-dibromophenoxy)-4,6-dibromophenol	sponge	other	0.13	-	
625	2-(4'-bromophenoxy)-3,5-dibromophenol	sponge	other	0.12	-	
626	5-bromo-2-(2',4'-dibromophenoxy) phenol	sponge	other	2.85	-	
627	2-(2',4'-dibromophenoxy)-5,6-dibromo-3-chlorophenol	sponge	other	5.54	-	
628	2-(2',4'-dibromophenoxy)-3,5,6-tribromophenol	sponge	other	0.31	-	
629	3,6-dibromo-2- (2',4'-dibromophenoxy) phenol	sponge	other	1.97	-	
630	mycousfuran 1	fungus	polyketide	32	-	(Lee et al. 2019)
631	mycousfuran 2	fungus	polyketide	-	-	
632	terretonin G	fungus	terpenoid	8	-	(H-L Li et al. 2019)
633	anthraquinone dimer	fungus	polyketide	-	-	(JL Li et al. 2019)
634	2-(4-hydroxybenzyl)-4-(3-acetyl)quinazolin-one	fungus	alkaloid	8	-	(Liu et al. 2019)
635	aromatic bisabolene-type sesquiterpenoid	fungus	other	18	-	
636	2-(4-hydroxybenzoyl)-4(3H)-quinazolinone	fungus	alkaloid	8	-	
637	chrysotriazole A	fungus	alkaloid	32	-	
638	pinselin	sponge	alkaloid	57	-	(Pang et al. 2019)
639	frangula-emodin	sponge	polyketide	3.75	-	
640	SBR-22	bacteria	polyketide	32	64	(Peela et al. 2019)
641	lamellomorphamide A	sponge	alkaloid	-	-	(Ragini et al. 2019)
642	meroindenon	bacteria	terpenoid	128	-	(Ryu et al. 2019)
643	merochlorin E	bacteria	terpenoid	2	-	
644	merochlorin F	bacteria	terpenoid	1	-	
645	merochlorin C	bacteria	terpenoid	32	-	

646	viscosine	bacteria	polyketide	-	-	(Selim et al. 2019)
647	penicipyrroether A	fungus	alkaloid	-	1.7	(Song et al. 2019)
648	purpurealidin K	sponge	alkaloid	50	-	(Tilvi et al. 2019)
649	purealidin Q	sponge	alkaloid	-	-	
650	aerophobin 2	sponge	alkaloid	-	-	
651	aplysamine 2	sponge	alkaloid	-	-	
652	2-hydroxy-6-formyl-vertixanthone	fungus	polyketide	-	16.3 ± 0.9	(Wang, Gao, et al. 2019)
653	12-O-acetyl-sydowinin A	fungus	polyketide	-	32.6 ± 0.8	
654	aspergillusone A	fungus	polyketide	-	32.2 ± 0.3	
655	AGI-B4	fungus	polyketide	-	32.9 ± 0.3	
656	8-demethoxy-10-methoxy-wentiquinone C	fungus	polyketide	-	32.4 ± 0.5	
657	penicitol D	fungus	polyketide	-	7.8 ± 0.8	(Wang, Liao, et al. 2019)
658	1-epi-citrinin H1	fungus	polyketide	-	7.3 ± 0.8	
659	penicitrinol A	fungus	other	-	16.3 ± 0.5	
660	(3*S*,4*S*)-sclerotinin A	fungus	other	-	15.2 ± 0.4	
661	(3*S*)-hydroxy-4-epi-isosclerone	fungus	polyketide	-	33.6 ± 0.2	
662	(3*R*,4*S*)-6,8-dihydroxy-1,1-dimethyl-3,4,5-trimethylisochroman	fungus	other	-	32.1 ± 0.4	
663	1-N-methyl-(*E*, *Z*)-albonoursin	bacteria	alkaloid	12.5	25	(Xu et al. 2019)
664	streptonigrin	bacteria	alkaloid	0.78	0.78	
665	atranone Q	fungus	terpenoid	-	32	(Yang et al. 2019)
666	18-acetyl-cyclooctatin	bacteria	other	-	9.99	(Yi et al. 2019)
667	5-dehydroxy-cyclooctatin	bacteria	other	-	9	
668	cyclooctatin	bacteria	other	-	21	
669	chromomycin A3	bacteria	polyketide	-	0.42	
670	5,18-dedihydroxy-cyclooctatin	bacteria	terpenoid	-	7	
671	streptoglutarimide A	bacteria	alkaloid	-	9	(D Zhang et al. 2019)
672	streptoglutarimide E	bacteria	alkaloid	-	-	
673	streptoglutarimide H	bacteria	alkaloid	-	-	

674	streptoglutarimide B	bacteria	alkaloid	-	11	
675	streptoglutarimide C	bacteria	alkaloid	-	10	
676	streptoglutarimide D	bacteria	alkaloid	-	10	
677	streptoglutarimide F	bacteria	alkaloid	-	10	
678	streptoglutarimide G	bacteria	alkaloid	-	10	
679	streptoglutarimide I	bacteria	alkaloid	-	11	
680	streptoglutarimide J	bacteria	alkaloid	-	10	
681	streptovitacin A	bacteria	alkaloid	-	10	
682	phallusialide A	bacteria	alkaloid	-	32	(F Zhang et al. 2019)
683	phallusialide B	bacteria	alkaloid	-	64	
684	penibenzophenones A	fungus	polyketide	20	-	(C-J Zheng et al. 2019)
685	peninaphone A	fungus	polyketide	25	12.5	(Y-Y Zheng et al. 2019)
686	peninaphone B	fungus	polyketide	-	-	
687	peninaphone C	fungus	polyketide	-	-	
688	nitricquinomycin C	bacteria	polyketide	17.04	-	(Zhou et al. 2019)
689	(2S)-1-O-(5Z,8Z,11Z,14Z,17Z-eicosapentaenoyl)-2-O-(9Z,12Z,15Z-octadecatrienoyl)-3-β-d-galacto-pyranosyl-sn-glycerol/MGDG	brown algae	aliphatic acid	-	-	(Buedenbender et al. 2020)
690	(2S)-1-O-(8Z,11Z,14Z,17Z-eicostetraenoyl)-2-O-(6Z,9Z,12Z,15Z-octadecatetranoyl)-3-β-d-galacto-pyranosyl-sn-glycerol/MGDG	brown algae	aliphatic acid	-	-	
691	digalactosyldiacylglycerol	brown algae	aliphatic acid	-	-	
692	2, 2-bis (6-bromo-1h-indole-3-yl) ethylamine	sponge	alkaloid	2	2	(Campana et al. 2020)
693	2, 2-bis (6-fluoro-1h-indole-3-yl) ethylamine	sponge	alkaloid	16.	32	
694	napyradiomycin B7a	bacteria	terpenoid	-	48	(Carretero-Molina et al. 2020)
695	napyradiomycin D1	bacteria	terpenoid	-	12–24	
696	napyradiomycin A2b	bacteria	terpenoid	-	12–24	

697	napyradiomycin B5	bacteria	terpenoid	-	12–24	
698	naphthoradiomycin B2	bacteria	terpenoid	-	3-6	
699	bacvalactone 1	bacteria	macrolide	-	3	(Chakraborty et al. 2020)
700	bacvalactone 2	bacteria	macrolide	-	3.12	
701	bacvalactone 3	bacteria	macrolide	-	1.5	
702	pseudocerolide C	sponge	peptide	-	4	(M Chen et al. 2020)
703	pseudoceroxime A	sponge	peptide	-	5	
704	pseudoceroxime B	sponge	peptide	-	4	
705	pseudoceroxime C	sponge	peptide	-	13	
706	pseudoceroxime D	sponge	peptide	-	15	
707	pseudoceroxime E	sponge	peptide	-	7	
708	pseudocerolide D	sponge	peptide	-	8	
709	pseudocerolide E	sponge	peptide	-	7	
710	maculosin-O-a-L-rhamnopyranoside	bacteria	alkaloid	28	37	(S Chen et al. 2020)
711	fmaculosin	bacteria	alkaloid	37	37	
712	collismycin A	bacteria	alkaloid	-	8	(Z Chen et al. 2020)
713	chromomycin A9	bacteria	polyketide	0.06−0.25	0.06−0.25	(Cho et al. 2020)
714	chromomycin Ap	bacteria	polyketide	0.06−0.25	0.06−0.25	
715	chromomycin A2	bacteria	polyketide	0.06−0.25	0.06−0.25	
716	helvolinic acid 18	fungus	steroid	6.25	-	(Han et al. 2020)
717	culvularin	sponge	macrolide	-	-	(Handayani, Amilia, et al. 2020)
718	sydowinin B	sponge	polyketide	-	-	
719	nidulin	sponge	polyketide	0.78	0.78	(Handayani, Rendowati, et al. 2020)
720	2-chlorounguinol	sponge	polyketide	1.56	0.78	
721	unguinol	sponge	polyketide	3.12	3.12	
722	andrastone C	fungus	terpenoid	-	8−9	(Kaleem et al. 2020)
723	andrastone B	fungus	terpenoid	-	-	
724	mersaquinone	bacteria	polyketide	-	3.36	(Kim et al. 2020)
725	spanned macrolide 1	bacteria	macrolide	-	3.00	(Kizhakkekalam et al.

						2020)
726	spanned macrolide 2	bacteria	macrolide	-	0.38	
727	spanned macrolide 3	bacteria	macrolide	-	1.50	
728	aryl-enclosed macrocyclic polyketide（2'-[(8-ethyl-8-methyl-2,5-dioxo-1-oxacyclododecanyl)methoxy]-methyl benzoate)	seaweed	polyketide	-	3.75	(Kizhakkepatt Kizhakkekalam et al. 2020)
729	1-O-methyl-averantin	fungus	polyketide	-	4.84	(Liu et al. 2020)
730	purpuride E	fungus	terpenoid	-	6	(Ma et al. 2020)
731	purpuride F	fungus	terpenoid	-	3	
732	1,3-Benzodioxole-2-one-4-carboxylamide	fungus	alkaloid	-	256	(Phi Thi et al. 2020)
733	chaetoglobosin B	echinoderm	alkaloid	25.26	50.52	(Qi et al. 2020)
734	5-trisindoline	sponge	alkaloid	-	3.54	(Sayed et al. 2020)
735	6-trisindoline	sponge	alkaloid	-	1.77	
736	serratiochelin A	bacteria	peptide	10.73	-	(Schneider et al. 2020)
737	3-(3-chloro-4-hydroxyphenyl) propionic acid	bacteria	phenol	32	-	(Shaala et al. 2020)
738	3-phenylpropionic acid	bacteria	phenol	125	-	
739	3-(3, 5-dichloro-4-hydroxyphenyl) propionic acid	bacteria	phenol	32	-	
740	methyl 3-(3, 5-dichloro-4-hydroxyphenyl) propionate	bacteria	phenol	32	-	
741	dionemycin	bacteria	alkaloid	1	2	(Song et al. 2020)
742	20-26 compound 3	bacteria	alkaloid	2	2−4	
743	salviamone B	fungus	polyketide	-	64	(Wu, Meng, et al. 2020)
744	(1*S*,4*R*,15*R*)-1, 18-epoxy-neocryptotanshinone	fungus	polyketide	-	128	
745	(1*S*,15*R*)-1-hydroxy-neocryptotanshinone	fungus	polyketide	-	128	
746	(1*R*,15*R*)-1- hydroxy-neocryptotanshinone	fungus	polyketide	-	128	
747	(15*R*)-3-hydroxy-neocryptotanshinone A	fungus	polyketide	-	128	

748	(15*R*)-3-hydroxy-neocryptotanshinone B	fungus	polyketide	-	128	
749	(15*R*)-1-keto-neocryptotanshinone	fungus	polyketide	-	64	
750	(15*R*)-3-keto-neocryptotanshinone	fungus	polyketide	-	32	
751	neocryptotanshinone	fungus	polyketide	-	128	
752	kipukasin K	fungus	other	8−16	-	(Wu, Yao, et al. 2020)
753	aspergillusene E	fungus	terpenoid	-	-	
754	3,7-dihydroxy-1,9-dimethyldibenzofuran	fungus	polyketide	3.32	-	(Yang et al. 2020)
755	monaprenylindole A	bacteria	alkaloid	-	45	(Yi et al. 2020)
756	aspergillactone	fungus	polyketide	2-16	-	(Cen et al. 2021)
757	lactoquinomycin A	bacteria	polyketide	-	0.25–0.5	(Chung et al. 2021)
758	N-methyl lactoquinomycin A	bacteria	polyketide	-	1	
759	menoxymycin A	bacteria	polyketide	-	2	
760	paxilline	fungus	terpenoid	128	-	(Dai et al. 2021)
761	dehydroxypaxilline	fungus	terpenoid	64	-	
762	7-hydroxyl-13-dehydroxypaxilline	fungus	terpenoid	64	-	
763	3-deoxo-4b-deoxypaxilline	fungus	terpenoid	64	-	
764	epipaxilline	fungus	terpenoid	64	-	
765	7-hydroxypaxilline-13-ene	fungus	terpenoid	64	-	
766	paspaline	fungus	terpenoid	64	-	
767	4a-demethylpaspaline-4a-carboxylic acid	fungus	terpenoid	64	-	
768	paspalinine	fungus	terpenoid	64	-	
769	PC-M6	fungus	terpenoid	64	-	
770	emindole SB	fungus	terpenoid	32	-	
771	Paralemnolin J	sponge	terpenoid	0.125	-	(Elshamy et al. 2021)
772	paralemnolide A	sponge	terpenoid	0.062	-	
773	parathyrsoidin G	sponge	terpenoid	0.6	-	
774	paralemnolin X	sponge	terpenoid	0.5	-	
775	paralemnolin Y	sponge	terpenoid	0.525	-	
776	paralemnolin D	sponge	terpenoid	0.302	-	
777	paralemnolin E	sponge	terpenoid	0.525	-	
778	paralemnolin P	sponge	terpenoid	0.636	-	

779	6α-acetyl-1(10)-α-13-nornardosin-7-one	sponge	terpenoid	0.709	-	
780	6α-acetyl-7α-acetate-1(10)-α-13-nornardosine	sponge	terpenoid	0.6	-	
781	clavukoellian F	sponge	terpenoid	0.333	-	
782	dizinchydroxyneoaspergillin	fungus	other	7.8	7.8	(C Guo et al. 2021)
783	hydroxyneoaspergillic acid	fungus	alkaloid	3.9	3.9	
784	actetrophenol A	bacteria	other	-	4μg/mL	(L Guo et al. 2021)
785	lulworthinone	fungus	polyketide	6.25	1.56–6.25	(Jenssen et al. 2021)
786	purpuride D	fungus	terpenoid	3	4	(Kaleem et al. 2021)
787	berkedrimane A	fungus	terpenoid	3	4	
788	berkedrimane B	fungus	terpenoid	3	4	
789	purpuride A	fungus	terpenoid	3	4	
790	purpuride B	fungus	terpenoid	3	4	
791	minioluteumide D	fungus	terpenoid	3	4	
792	2,5-Bis(3-indolylmethyl)pyrazine	fungus	alkaloid	3	4	
793	L-N-acetyltryptophanol	fungus	alkaloid	3	4	
794	L-tryptophane	fungus	alkaloid	3	4	
795	tryptophol	fungus	alkaloid	3	4	
796	tryptamine	fungus	alkaloid	3	4	
797	mucorisocoumarin B	fungus	polyketide	3	4	
798	maculosin	fungus	alkaloid	30	19	
799	4-hydroxymethyl-quinoline	fungus	alkaloid	3	4	
800	micotinamide	fungus	alkaloid	3	4	
801	uridine	fungus	alkaloid	30	19	
802	purpurester A	fungus	polyketide	3	4	
803	phorbaketal C	sponge	other	-	-	(Kim et al. 2021)
804	phorbaketal B	sponge	other	-	-	
805	aspergilluone A	sponge	other	64	-	(Liu et al. 2021)
806	lobophorin B	bacteria	terpenoid	64	64	(Luo et al. 2021)
807	divergolide R	bacteria	terpenoid	32	32	(Luo et al. 2021; Tu et al. 2024)
808	aspulvinone B	sponge	terpenoid	16	16	(Machado et al. 2021)

809	aspulvinone H	sponge	terpenoid	16	16	
810	aspulvinone A	sponge	terpenoid	64	64	
811	aspulvinone R	sponge	terpenoid	8	8−16	
812	aspulvinone S	sponge	terpenoid	4	8−16	
813	aspulvinone T	sponge	terpenoid	＞64	＞64	
814	aspulvinone U	sponge	terpenoid	64	64	
815	stachybomycin E	fungus	steroid	16	16	(Mou et al. 2021)
816	stachybotrylactam acetate	fungus	steroid	16	16	
817	spiromastixone R	fungus	polyketide	1	-	(Niu et al. 2021)
818	spiromastixone P	fungus	polyketide	8	-	
819	spiromastixone Q	fungus	polyketide	4	-	
820	spiromastixone S	fungus	polyketide	32	-	
821	spiromastimellein A	fungus	polyketide	32	-	
822	spiromastimellein B	fungus	polyketide	16	-	
823	penicillenol H	fungus	other	2.5	2.5	(Pang, Chen, et al. 2021)
824	sohirnone A	fungus	phenol	10	20	(Pang, Zhou, et al. 2021)
825	dihydrodemethylsorbicillin	fungus	phenol	5	10	
826	5-hydroxy-dihydrodemethylsorbicillin	fungus	phenol	80	80	
827	2-deoxysohirnone C	fungus	phenol	-	40	
828	tetrahydrotrichodimerol	fungus	phenol	80	40	
829	dihydrotrichodimerol	fungus	phenol	80	80	
830	merochlorin I	bacteria	terpenoid	2	-	(Ryu et al. 2021)
831	merochlorin G	bacteria	terpenoid	16	-	
832	merochlorin A	bacteria	terpenoid	4	-	
833	merochlorin D	bacteria	terpenoid	1	-	
834	fusaripyridine A	fungus	alkaloid	≥13.7	-	(Shaala et al. 2021)
835	fusaripyridine B	fungus	alkaloid	≥14.2	-	
836	aspergiloxathene A	fungus	polyketide	3.13	12.5	(Song et al. 2021)
837	7-hydroxyoospolactone	fungus	polyketide	12.5	-	(Xu et al. 2021)
838	parapholactone	fungus	polyketide	12.5	-	
839	ergochrome C	fungus	polyketide	-	26	(Yong et al. 2021)

840	ergochrome D	fungus	polyketide	-	29	
841	ergochrome E	fungus	polyketide	-	16	
842	ergochrome F	fungus	polyketide	-	24	
843	ergochrome G	fungus	polyketide	-	35	
844	2-benzoxazolol	fungus	polyketide	-	6	
845	fTMKS8A	aplysia	polyketide	12.5	-	(Zhang et al. 2021)
846	cysrabelomycin	bacteria	polyketide	9.3	-	(Zhou et al. 2021)
847	xishaeleganin B	sponge	terpenoid	1.5	-	(Chen et al. 2022)
848	dictyoceratin A	sponge	terpenoid	2.9	-	
849	xishaeleganin C	sponge	terpenoid	11.1	-	
850	dactyloquinone C	sponge	terpenoid	11.1	-	
851	dictyoceratin B	sponge	terpenoid	12.1	-	
852	dactyloquinone B	sponge	terpenoid	178	-	
853	acresorbicillinol B	fungus	other	-	-	(Duan et al. 2022)
854	sterigmatocystin	fungus	polyketide	32	64	(Handayani et al. 2022)
855	iso-laurenisol	red algae	terpenoid	16	-	(Harizani et al. 2022)
856	bromolaurenisol	red algae	terpenoid	4	-	
857	prodigiosin	bacteria	other	-	1.56	(He et al. 2022)
858	2-methyl-3-heptyl-prodiginine	bacteria	other	-	1.56	
859	2-methyl-3-propyl-prodiginine	bacteria	other	-	50	
860	2-methyl-3-butyl-prodiginine	bacteria	other	-	50	
861	taladrimanin A	fungus	terpenoid	-	15.2	(Hong et al. 2022)
862	desferrioxamine B	brown algae	other	-	-	(Katif et al. 2022)
863	Resistomycin	bacteria	polyketide	-	0.125	(MC Kim et al. 2022)
864	chlororesistoflavin A	bacteria	polyketide	-	0.25	
865	chlororesistoflavin B	bacteria	polyketide	-	2.0	
866	resistoflavin	bacteria	polyketide	-	0.25	
867	neoasterric methyl ester	bacteria	other	64	-	(S Kim, TC Le, et al. 2022)
868	penicitrinon E	bacteria	polyketide	2	-	
869	(2*E*)-1-[(5-hydroxy-7-methoxy-2-methyl-4-oxo-4H-	fungus	polyketide	2	4	(Lai et al. 2022)

	1-benzopyran-3-yl)methyl]3-methyl-2-pentenedioate					
870	(2S,3S)-5-hydroxy-3-hydroxymethyl-7-methoxy-2-methyl-4-chromanone	fungus	polyketide	32	32	
871	(+)-aeroplysinin-1	sponge	alkaloid	-	32	(Lever et al. 2022)
872	ligiamycin A	bacteria	terpenoid	16	-	(Lim et al. 2022)
873	ligiamycin B	bacteria	terpenoid	64	-	
874	D-tryptophanα-aminoamide	whelk	alkaloid	32	-	(Lim et al. 2022; Linh et al. 2022)
875	chrysostorine	whelk	alkaloid	64	-	
876	N-(2-(1H-indol-3-yl)ethyl)−2-phenylacetamide	whelk	alkaloid	128	-	
877	inosine	whelk	alkaloid	64	-	
878	l-thymidine	whelk	alkaloid	64	-	
879	5-prenylcryptoechinulin A	fungus	alkaloid	114.32	-	(Lv et al. 2022)
880	cryptoechinulin A	fungus	alkaloid	101.3	-	
881	neoechinulin B	fungus	alkaloid	80.27	-	
882	7-prenylneoechinulin B	fungus	alkaloid	89.3	-	
883	talaroisocoumarin A	fungus	polyketide	-	36	(Ma et al. 2022)
884	5[(3*E*,5*E*)-nona-3,5-dien-1-yl]benzene-1,3-diol	fungus	other	16	16	(Machado et al. 2022)
885	siphonellinol C	sponge	terpenoid	6.25	12.5	(Musa et al. 2022)
886	callysterol	sponge	steroid	6.25	-	
887	sipholenol A	sponge	terpenoid	12.5	-	
888	sipholenone A	sponge	terpenoid	12.5	25	
889	petroselenic acid	sponge	other	25	-	
890	streptoindole A	bacteria	alkaloid	25	-	(Newaz et al. 2022)
891	streptoindole D	bacteria	alkaloid	14	-	
892	streptoindole B	bacteria	alkaloid	7	-	
893	α,2,5-trihydroxyacetophenone	fungus	phenol	200	128	(Ningsih et al. 2022)
894	(+)-10-epiagelasine B	sponge	alkaloid	1−2	-	(Pech-Puch et al. 2022)
895	(+)-8-epiagelasine T	sponge	alkaloid	16	-	

896	(+)-12-hydroxyagelasidine C	sponge	alkaloid	8	-	
897	(+)-ent-agelasine F	sponge	alkaloid	4	-	
898	(+)-agelasine B	sponge	alkaloid	2	-	
899	(+)-agelasidine C	sponge	alkaloid	8	-	
900	4α-hydroxy-17-methylincisterol	fungus	steroid	32	-	(Ren et al. 2022)
901	rugulosin A	fungus	polyketide	0.195	-	(Song, Dong, et al. 2022)
902	bacillisporin K	fungus	polyketide	12.5	-	
903	bacillisporin L	fungus	polyketide	25	-	
904	bacillisporin B	fungus	polyketide	12.5	-	
905	macrosporusone D	fungus	polyketide	6.25	-	
906	penicillide	fungus	polyketide	100	-	
907	thiolopyrrolone A	bacteria	alkaloid	100	-	(Song, Hu, et al. 2022)
908	thiolutin	bacteria	other	3.125	-	
909	discolin A	bacteria	alkaloid	64	-	(L Wang, V Linares-Otoya, et al. 2022)
910	discolin B	bacteria	alkaloid	64	-	
911	dispyrrolopyridine A	bacteria	alkaloid	0.5-1	-	
912	dispyrrolopyridine B	bacteria	alkaloid	4	-	
913	dispyrrole	bacteria	alkaloid	8	-	
914	discolin E	bacteria	alkaloid	8	-	(L Wang, M Marner, et al. 2022)
915	discolin C	bacteria	alkaloid	16	-	
916	discolin H	bacteria	alkaloid	32	-	
917	5-bromoisorotiorin	fungus	polyketide	-	-	(Z Wang et al. 2022)
918	5-bromosclerotiorin	fungus	polyketide	-	-	
919	penicilazaphilone H	fungus	polyketide	-	-	
920	hygrocin N	bacteria	macrolide	15	-	(Yi et al. 2022)
921	hygrocin O	bacteria	polyketide	24	-	
922	hygrocin R	bacteria	polyketide	9	-	
923	hygrocin T	bacteria	polyketide	44	-	
924	2-amino-6-hydroxy-7-methyl-1,4-naphthoquinone	bacteria	polyketide	10	-	
925	3'-methoxy(1,1',4',1”-terphenyl)-2',6'-diol	bacteria	other	5	-	

926	echoside C	bacteria	other	6	-	
927	echoside A	bacteria	other	8	-	
928	hygrocin U	bacteria	polyketide	3	-	
929	2-acetamide-6-hydroxy-7-methyl-1,4-naphthoquinone	bacteria	polyketide	3	-	
930	asperopiperazine A	fungus	peptide	2.83	-	(Youssef et al. 2022)
931	asperopiperazine B	fungus	peptide	2.1	-	
932	(+)-citreoisocoumarin	fungus	polyketide	2.22	-	
933	(-)-6,8-di-O-methylcitreoisocoumarin	fungus	polyketide	5	-	
934	5'-methoxy-6-methyl-biphenyl-3,4,3'-triol	fungus	polyketide	50	-	(K Zhang et al. 2022)
935	asperfumigatin	fungus	alkaloid	2.5	-	(R Zhang et al. 2022)
936	12,13-dihydroxyfumitremorgin C	fungus	alkaloid	1.025	-	
937	fumitremorgin B	fungus	alkaloid	9.6	-	
938	spirotryprostatin C	fungus	alkaloid	4.95	-	
939	(-)-chaetominine	fungus	alkaloid	10	-	
940	fumigaclavine C	fungus	alkaloid	4.58	-	
941	11-O-methylpseurotin A	fungus	alkaloid	4.45	-	
942	azaspirofuran B	fungus	alkaloid	2	-	
943	azaspirofuran A	fungus	alkaloid	2.05	-	
944	fumagiringillin	fungus	terpenoid	11.9	-	
945	fumagillin	fungus	terpenoid	1.15	-	
946	penibenzophenone E	fungus	other	0.415	-	
947	sulochrin	fungus	other	0.415	-	(R Huang et al. 2024; R Zhang et al. 2022)
948	dimethyl-2,3'-dimethylosoate	fungus	other	1.88	-	
949	13-oxofumitremorgin B	fungus	alkaloid	0.59	-	
950	6-O-propionyl-16-O-deacetylhelvolic acid	fungus	steroid	0.73	-	
951	16-O-propionyl-6-O-deacetylhelvolic acid	fungus	steroid	1.25	-	
952	aspergillusether J	fungus	phenol	-	16	(Y Zhang et al. 2022)
953	aspergillusidone C	fungus	phenol	-	32	
954	guisinol	fungus	phenol	-	16	
955	aspergillusether F	fungus	phenol	-	2	
956	nornidulin	fungus	polyketide	-	2	

957	acrucipentyn F	fungus	polyketide	39.4	-	(Zhuravleva et al. 2022)
958	spiromastol L	fungus	other	1−2	2	(Cai et al. 2023)
959	spiromastol M	fungus	polyketide	8-32	8	
960	spiromastol N	fungus	polyketide	4-8	4	
961	spiromastixone La	fungus	other	8-16	8	
962	spiromastixone L	fungus	polyketide	4-16	8	
963	spiromastixone G	fungus	polyketide	2-4	2	
964	aspertide F	fungus	peptide	8	-	(Chi et al. 2023)
965	12-bromo-communesin A	fungus	alkaloid	-	-	(Cochereau et al. 2023)
966	4-bromo-5-methyl-2-(1,2,2-trimethylcyclopent-3-en-1-yl) phenol	red algae	terpenoid	50	-	(Fakee et al. 2023)
967	2-clorounguinol	fungus	polyketide	-	2	(Handayani et al. 2023)
968	phocoenamicin D	bacteria	polyketide	-	64	(Kokkini et al. 2023)
969	phocoenamicin E	bacteria	polyketide	-	32−64	
970	maklamicin	bacteria	polyketide	-	< 0.26	
971	maklamicin B	bacteria	polyketide		1.02	
972	(+)-isoquinocitrinin B	fungus	alkaloid	2	2	(Lai et al. 2023)
973	(-)-isoquinocitrinin B	fungus	alkaloid	2	2	
974	aspergetherin A	fungus	polyketide		128	(Li et al. 2023)
975	aspergetherin C	fungus	biphenyl		64	
976	methyl chloroasterrate	fungus	biphenyl		64	
977	methyl 3,5-dichloroasterric acid	fungus	biphenyl		1−16	
978	5-deoxybostrycoidin	fungus	polyketide	12	6-12	
979	fusarisolin H	fungus	polyketide	12	6-12	
980	fusarisolin I	fungus	polyketide	25	3−25	(C Lin et al. 2023)
981	fusarisolin J	fungus	polyketide	12	3−6	
982	bacillisporin A	fungus	polyketide	4	4	(Machado et al. 2023)
983	sch 1385568	fungus	polyketide	64	16	
984	(±)-hamiavemin A	fungus	alkaloid	128	-	(Minh et al. 2023)

985	monomethylsulochrin 5-sulfonic acid	fungus	other	64	-	
986	monomethylsulochrin	fungus	other	16	-	
987	questin	fungus	polyketide	256	-	
988	trans-p-coumaroyl-L-tryptophan	fungus	alkaloid	128	-	
989	ergosterol	fungus	steroid	64	-	
990	3-(4-hydroxyphenyl)pyrazin-2-ol	fungus	alkaloid	64	-	
991	isosclerone	fungus	polyketide	256	-	
992	(*S*)-2-hydroxy-2-((*R*)-4-hydroxy-6-methoxy-1,7-dimethyl-3-oxo-1,3-dihydroisobenzofuran-1-yl)ethyl acetate	fungus	polyketide	31	-	(Pan et al. 2023)
993	nectriapyrone A	fungus	polyketide	22	-	
994	(*R*)-3,4-dihydro-4,6,8-trihydroxy-4,5-dimethyl-3-methyleneisochromen-1-one	fungus	polyketide	22	-	
995	phenamide	bacteria	other	0.04	-	(Qader et al. 2023)
996	isocyanogramide	bacteria	alkaloid	64	-	(Quynh et al. 2023)
997	caerulomycinamide	bacteria	alkaloid	64	-	
998	cyanogramide	bacteria	alkaloid	128	-	
999	marinacarboline F	bacteria	alkaloid	128	-	
1000	marinacarboline H	bacteria	alkaloid	128	-	
1001	cerulomyconitrile	bacteria	alkaloid	128	-	
1002	5'-deoxyuridine	bacteria	alkaloid	256	-	
1003	secodinorspongin A	sponge	terpenoid	16	-	(Tai et al. 2023)
1004	7-dechloronidulin	fungus	polyketide	4	-	(Thi Hoang Anh et al. 2023)
1005	2,4-dichlorounguinol	fungus	polyketide	32	-	
1006	2-chlorounguinol C	fungus	polyketide	32	-	
1007	emeguisin A	fungus	polyketide	128	-	
1008	emeguisin B	fungus	polyketide	256	-	
1009	cerevisterol	microalgae	steroid	32	-	(Thuy et al. 2023)
1010	(24*R*)-ergosta-7,22-diene-3β,5α,9α-triol	microalgae	steroid	64	-	
1011	ergosta-7,22-diene-3β,5α-diol	microalg	steroid	128	-	

		ae				
1012	(22*E*,24*S*)-5α,6α-epoxy-ergosta-8(9),22-diene-3β,7α-diol	microalgae	steroid	64	-	
1013	5α,8α-ep idioxyergosta-6,22-diene-3β-ol	microalgae	steroid	64	-	
1014	nocardiopyrone C	bacteria	other	10.1	-	(Tian et al. 2023)
1015	nocardiopyrone B	bacteria	other	10.7	-	
1016	1-hydroxy-4-methoxy-2-naphthoic acid	bacteria	other	10.9	-	
1017	secalonic acid D	fungus	polyketide	2	-	(Wu et al. 2023)
1018	*R*-wailupemycin K	bacteria	polyketide	32	-	(Xia et al. 2023)
1019	*S*-wailupemycin K	bacteria	polyketide	32	-	
1020	wailupemycin S	bacteria	polyketide	4	4	
1021	phyllofenone I	sponge	terpenoid	16	-	(Yu et al. 2023)
1022	phyllofenone K	sponge	terpenoid		-	
1023	cholesterol	sponge	steroid	1.5	-	(Bashar et al. 2024)
1024	asterripeptide A	fungus	peptide	48.73	-	(Chingizova et al. 2024)
1025	asterripeptide B	fungus	peptide	48.73	-	
1026	asterripeptide C	fungus	peptide	48	-	
1027	marinopyrrole A	bacteria	other	-	0.75±0.09	(Guo et al. 2024)
1028	20*R*, 25*R*-preshamixanthone	fungus	polyketide	-	50	(Ha et al. 2024)
1029	25*R*-O-methylarugosin A	fungus	polyketide	-	50	
1030	violaceol-I	fungus	phenol	-	50	
1031	violaceol-II	fungus	phenol	-	50	
1032	4-methoxycarbonyldiorcinol	fungus	phenol	-	50	
1033	4-carbethoxydiorcinal	fungus	phenol	-	25	
1034	aspergilol C	fungus	terpenoid	-	50	
1035	2-hydroxyemodic acid	fungus	polyketide	-	6.25	
1036	2ω-dihydroxyemodin	fungus	polyketide	-	6.25	
1037	ω-hydroxyemodin	fungus	polyketide	-	50	
1038	asperthecin	fungus	polyketide	-	12.5	
1039	austin	fungus	polyketide	-	25	
1040	lemnabourside	coral	terpenoid	8	-	(Han et al. 2024)

1041	biofloranate E	coral	terpenoid	32	-	
1042	biofloranate F	coral	terpenoid	32	-	
1043	biofloranate G	coral	terpenoid	64	-	
1044	biofloranate H	coral	terpenoid	64	-	
1045	biofloranate I	coral	terpenoid	64	-	
1046	lemnabourside H	coral	terpenoid	16	-	
1047	lemnabourside I	coral	terpenoid	32	-	
1048	spriomastixone Z1	fungus	polyketide	-	0.29	(Z Huang et al. 2024)
1049	spiromastixone U	fungus	polyketide	-	13.0	
1050	spiromastixone V	fungus	polyketide	-	3.9	
1051	spiromastixone W	fungus	polyketide	-	7.8	
1052	spiromastixone X	fungus	polyketide	-	2.24	
1053	spiromastixone Y	fungus	polyketide	-	2.24	
1054	spiromastixone Z	fungus	polyketide	-	1.15	
1055	spiromastixone Z2	fungus	polyketide	-	0.575	
1056	spiromastixone Z3	fungus	polyketide	-	1.31	
1057	spiromastixone Z4	fungus	polyketide	-	1.15	
1058	spiromastixone Z5	fungus	polyketide	-	5.22	
1059	spiromastixone T	fungus	polyketide	-	1.04	
1060	(±)-pestalachloride D	fungus	polyketide	50	-	(L Jiang et al. 2024)
1061	asperbutenolide A	fungus	other	-	4-8	(Y Jiang et al. 2024)
1062	halenaquinol	sponge	polyketide	-	128	(Jaepil Lee et al. 2024)
1063	halenaquinol sufate	sponge	polyketide	-	128	
1064	13,14,15,16-tetrahydroxestoquinol	sponge	polyketide	-	128	
1065	xestoquinol sulfate	sponge	polyketide	-	128	
1066	secoadociaquinone B	sponge	polyketide	-	128	
1067	xestolactone A	sponge	polyketide	-	128	
1068	tunicamycin VII	bacteria	other	0.13	8	(Jayho Lee et al. 2024)
1069	tunicamycin VIII	bacteria	other	0.13	8	
1070	corynetoxin U17a	bacteria	other	0.06	2	
1071	tunicamycin IX	bacteria	other	0.25	8	
1072	12*S*-aspertetranone D	fungus	alkaloid	-	3.75	(Sun et al. 2024)
1073	aspyrone	fungus	alkaloid	-	20	
1074	stonikacidin A	sponge	alkaloid	-	-	(Tabakmakher et al. 2024)

1075	quinosumycin	bacteria	alkaloid	64	8	(Tan et al. 2024)
1076	chromycone	bacteria	alkaloid	64	32	
1077	taladrimanin B	fungus	terpenoid	12.5	-	(Tang et al. 2024)
1078	tulongicin A	sponge	alkaloid	1-2	1-2	(Tsuruda et al. 2024)
1079	diaportinol	fungus	polyketide	200	-	(Wang et al. 2024)
1080	talarohydrazone A	fungus	alkaloid	32	-	(Wu et al. 2024)
1081	talarohydrazone B	fungus	alkaloid	64	-	
1082	talarohydrazone C	fungus	alkaloid	128	128	
1083	talarohydrazone D	fungus	alkaloid	128	64	
1084	2,4′-linked secalonic acid	fungus	polyketide	3.125		(Xu, Dong, et al. 2024)
1085	7-hydroxy-13-dehydroxypaxille	fungus	terpenoid	> 128	16	(L Yang et al. 2024)
1086	4α-demethylpaspaline-4α-carboxylic acid	fungus	terpenoid	32	32	
1087	seco-geldanamycin B	fungus	alkaloid	64	64	(Yi et al. 2024)
1088	4,5-dihydro-17-O-demethylgeldanamycin	fungus	alkaloid	64	64	
1089	4,5-dihydro-19-S-methylgeldanamycin	fungus	alkaloid	64	-	
1090	seco-geldanamycin A	fungus	alkaloid	64	64	
1091	vibridiazinane A	fungus	alkaloid	0.98	-	(Zha et al. 2024)
1092	vibripyrrolidine A	fungus	alkaloid	1.5	-	
1093	vibridiazinane B	fungus	alkaloid	3.9	-	
1094	1, 2-diethyl-diazinane	fungus	other	0.98	-	
1095	tetraethyl hydrazine	fungus	other	7.81	-	
1096	purpuride B	fungus	steroid	-	2	(W Zhang et al. 2024)
1097	penicitrinone A	fungus	polyketide	1	-	
1098	2,3,4-trimethyl-5,7-dihydroxy-2,3-dihydrobenzofuran	fungus	polyketide	1	-	
1099	purpuride	fungus	polyketide	-	8	
1100	maduraflavacin A	bacteria	other	-	-	(Zou et al. 2024)
1101	cotteslosin D	fungus	peptide	-	-	(Zheng et al. 2024)

1102	butyrolactone J	fungus	phenol	12.5	-	(Xinwan Zhang et al. 2024)
1103	methyl 6-acetyl-4-methoxy-5,7,8-trihydroxynaphthalene-2-carboxylate	fungus	polyketide	25	-	
1104	territrem A	fungus	polyketide	100	-	
1105	penicillanthranin A	fungus	polyketide	-	16	(Thamsiri et al. 2024)
1106	penicisteckin G	fungus	biphenyl	-	4	(X Huang et al. 2024)
1107	penicisteckin H	fungus	biphenyl	-	4	
1108	[(2*R*)-2,3-dihydroxypropyl] (2*R*)-2-hydroxypentadecanoate	coral	other	-	-	(Farouk et al. 2024)
1109	myristyl palmitate	coral	other	-	-	
1110	(Z)-2-(9-octadecenyloxy) ethanol	coral	other	-	-	
1111	24-methylene cholesterol	coral	other	-	-	
1112	23-demethylgorgosterol	coral	other	-	-	
1113	1,2-seco-trypacidin	fungus	polyketide	6.25	-	(Chen et al. 2024)
1114	β-Indomycinone	bacteria	polyketide	13.7	-	(Ghandourah 2025)
1115	γ-indomycinone	bacteria	polyketide	10.3	-	
1116	dysidamide B	sponge	other	64	-	(Tammam et al. 2025)
1117	dysidamide D	sponge	other	32	-	
1118	dysidamide E	sponge	other	4	-	
1119	coscilide D	sponge	other	32	-	(Tran et al. 2025)
1120	antarmycin A	bacteria	macrolides	-	1-4	(Zhou et al. 2025)
1121	antarmycin B	bacteria	macrolides	-	16-64	
1122	antarmycin C	bacteria	macrolides	-	0.25-1	
1123	rakicidin B1-2	fungus	peptide	8	16	(L Chen et al. 2020a)
1124	rakicidin J	fungus	peptide	8	> 16	(L Chen et al. 2020b)
1125	lithocarpinol C	fungus	polyketide	10.2	-	(Li et al. 2020)
1126	lithocarpinol D	fungus	polyketide	20.4	-	

1127	1*E*,3*R*,4*S*,5*E*,7*Z*-1-bromo-3,4,8-trichloro-7-(dichloromethyl)-3-methylocta-1,5,7-triene	red algae	terpenoid	108	-	(Shiyanga et al. 2020)
1128	julichrome Q10	bacteria	polyketide	32	16-32	(Dong et al. 2020)
1129	julichrome Q11	bacteria	polyketide	32	32	
1130	julichrome Q6.6	bacteria	polyketide	32	16	
1131	julichrome Q6	bacteria	polyketide	32-64	8-32	
1132	β-diversonolic ester	fungus	polyketide	10.4	46.9	(Guo et al. 2020)
1133	penibenzophenone C	fungus	polyketide	6.25	3.12	(Bai et al. 2021)
1134	penibenzophenone D	fungus	polyketide	12.5	6.25	
1135	hydoxysulochrin	fungus	polyketide	6.25	> 25	
1136	(3*S*,6*S*)-3,6-diisobutylpiperazine-2,5-dione	sponge	peptide	22	-	(Bhattacharya et al. 2021)
1137	2-ethylhexyl 1H-imidazole-4-carboxylate	bacteria	alkaloid	16	-	(M-H Chen et al. 2021)
1138	terrusnolide A	fungus	phenol	6.25	-	(W Chen et al. 2021)
1139	berninamycin A	bacteria	peptide	0.8	0.4	(De et al. 2021)
1140	berninamycin B	bacteria	peptide	4	1	
1141	berninamycin K	bacteria	peptide	8	8	
1142	simplifusidic acid I	fungus	steroid	0.078	-	(Cheng et al. 2021)
1143	simplifusidic acid H	fungus	steroid	2.5	-	
1144	tianyamycin A	bacteria	alkaloid	0.125	0.125	(L Guo et al. 2021)
1145	tianyamycin B	bacteria	alkaloid	0.125	0.125	
1146	tianyamycinC	bacteria	alkaloid	0.125	0.125	
1147	tianyamycin D	bacteria	alkaloid	0.125	0.125	
1148	L-N-acetyltryptophanol	bacteria	peptide	1.25	12.5	(Hamed et al. 2021)
1149	L-tryptophane	bacteria	peptide	0.025	0.1	
1150	tryptophol	bacteria	peptide	0.156	0.78	
1151	tryptamine	bacteria	peptide	0.156	0.78	
1152	sartorypyrone A	fungus	terpenoid	1−2	-	(Lv et al. 2021)
1153	myticalin A6	mussel	peptide	8	-	(Okimura et al. 2021)

1154	(*S*)-2-methyl-1-(4-oxo-3, 4-dihydroquinazolin-2-yl) Pr acetate	fungus	alkaloid	4	-	(Wang et al. 2021)
1155	brocazine G	fungus	alkaloid	0.25	-	(Fernando et al. 2022)
1156	actinopolymorphol F	bacteria	alkaloid	64		(S Kim, PF Hillman, et al. 2022)
1157	4-methyl-5,6-dihydro-2H-pyran-2-one	fungus	terpenoid	40	40	(Zaman et al. 2022)
1158	vermistatin	fungus	terpenoid	20	20	
1159	FK17-P2b1	fungus	terpenoid	10	10	
1160	FWYZ52-A	fungus	macrolides	-	-	(Zhao et al. 2022)
1161	aspergimarin G	fungus	polyketide	16−64	-	(S Lin et al. 2023)
1162	peniprenylphenol A	fungus	phenol	-	6	(Newaz et al. 2023)
1163	oxaliterpenoid	fungus	terpenoid	32	-	(Nguyen et al. 2023)
1164	aspergillusether A	fungus	terpenoid	64	-	
1165	aspergillusether J	fungus	terpenoid	8	-	
1166	guisinol	fungus	terpenoid	4	-	
1167	sarcophytonin B	coral	terpenoid	<0.5	-	(Ren et al. 2023)
1168	1,13-di-epi-13-acetoxy launine P	coral	terpenoid	16	-	
1169	13-oxo-thunbergol	coral	terpenoid	64	-	
1170	launine P	coral	terpenoid	32	-	
1171	(2*E*,4*E*)-1-(2,6-dihydroxy-3,5-dimethyl-phenyl)hexa-2,4-dien-1-one	fungus	other	128	-	(An et al. 2024)
1172	sorbicillin	fungus	other	128	-	
1173	β-carotene	brown algae	terpenoid	0.1125	-	(Hadjkacem et al. 2024)
1174	anserinone C	fungus	polyketide	21	-	(Hong et al. 2024)
1175	xiamycin	bacteria	terpenoid	-	32	(G Huang et al. 2024)
1176	chloroxiamycin	bacteria	terpenoid	-	16	
1177	2,4,6,9-tetrahydroxy-7-methyl-2-prenyl-1H-phenalene-1,3(2H)-dione	fungus	polyketide	50	25	(Morehouse et al. 2024)

1178	malfilanol C	fungus	terpenoid	-	-	(Niu et al. 2024)
1179	streptomine A	bacteria	alkaloid	-	-	(Pham et al. 2024)
1180	debilon	red algae	terpenoid	-	-	(Salman et al. 2024)
1181	phorbasterone-B	red algae	terpenoid	-	-	
1182	3β-hydroxy-5α,6β-methoxyergosta-7,22-dien-15-one	fungus	steroid	-	-	(Wen et al. 2024)
1183	asperbrunneo acid	fungus	terpenoid	200	-	(Xu, Han, et al. 2024)
1184	secalonic acid F1	fungus	polyketide	25	-	
1185	secalonic acid H	fungus	polyketide	50	-	
1186	penicillixanthone A	fungus	polyketide	6.25	-	
1187	chrysoxanthone C	fungus	polyketide	50	-	
1188	asperdichrome	fungus	polyketide	25	-	
1189	1,6-dihydroxy-3-methoxy-8-methylxanthone	fungus	polyketide	50	-	(J Yang et al. 2024)
1190	1,3,6-trihydroxy-8-methylxanthone	fungus	polyketide	12.5	-	
1191	alternariol	fungus	polyketide	50	-	
1192	lumichrome	fungus	alkaloid	200	-	(Xinjun Zhang et al. 2024)

-: Unreported

Supplementary Table S3. The source distribution of marine bacterial-derived active compounds

**Species**	*Streptomyces* sp.	*Actinomyces* sp.	*Bacillus* sp.	other
**Proportion**	35.7%	28.6%	14.3%	21.4%

References

Abdel-Lateff A, Elkhayat ES, Fouad MA, Okino T (2009) Aureobasidin, new antifouling metabolite from marine-derived fungus *Aureobasidium* sp. Nat Prod Commun 4(3):389−394

Abou-Shoer MI, Shaala LA, Youssef DTA, Badr JM, Habib A-AM (2008) Bioactive brominated metabolites from the Red Sea sponge *Suberea mollis*. J Nat Prod 71(8):1464−1467

Ahmad S, Nazir M, Tousif MI, Saleem M, Mustafa R, Khatoon T (2019) A new polyketide antibiotic from the marine bacterium *Streptomyces* sp. PGC 32. Chem Nat Compd 55(1):1−4

Al-Footy KO, Alarif WM, Asiri F, Aly MM, Ayyad S-EN (2015) Rare pyrane-based cembranoids from the Red Sea soft coral *Sarcophyton trocheliophorum* as potential antimicrobial-antitumor agents. Med Chem Res 24(2):505−512

Al-Footy KO, Alarif WM, Zubair MS, Ghandourah MA, Aly MM (2016) Antibacterial and cytotoxic properties of isoprenoids from the red sea soft coral, *Lobophytum* sp. Trop J Pharm Res 15(7):1431−1438

Alarif WM, Al-Lihaibi SS, Ayyad S-EN, Abdel-Rhman MH, Badria FA (2012) Laurene-type sesquiterpenes from the Red Sea red alga *Laurencia obtusa* as potential antitumor-antimicrobial agents. Eur J Med Chem 55:462−466

An CL, Kong FD, Ma QY, Xie QY, Yuan JZ, Zhou LM, Dai HF, Yu ZF, Zhao YX (2018) Chemical constituents of the marine-derived fungus *Aspergillus* sp. SCS-KFD66. Mar Drugs 16(12):468

An CL, Ma QY, Xie QY, Yang L, Yuan JZ, Dai HF, Zhao YX (2024) Chemical constituents of the marine-derived fungus *Acremonium* sp. AN-13. J Asian Nat Prod Res 26(4):489−496

Anjum K, Kaleem S, Yi W, Zheng G, Lian X, Zhang Z (2019) Novel antimicrobial indolepyrazines A and B from the marine-associated *Acinetobacter* sp. ZZ1275. Mar Drugs 17(2):89

Aqil F, Zahin M, El Sayed KA, Ahmad I, Orabi KY, Arif JM (2011) Antimicrobial, antioxidant, and antimutagenic activities of selected marine natural products and tobacco cembranoids. Drug Chem Toxicol (1977) 34(2):167−179

Asolkar RN, Singh A, Jensen PR, Aalbersberg W, Carte BK, Feussner K-D, Subramani R, Di Pasquale A, Rheingold AL, Fenical W (2017) Marinocyanins, cytotoxic bromo-phenazinone meroterpenoids from a marine bacterium from the *Streptomycete* clade MAR4. Tetrahedron 73(16):2234−2241

Bagalagel AA, Bogari HA, Ahmed SA, Diri RM, Elhady SS (2018) New bromoindole alkaloid isolated from the marine sponge *Hyrtios erectus*. Heterocycles 96(4):749−756

Bai M, Gao CH, Liu K, Zhao LY, Tang ZZ, Liu YH (2021) Two new benzophenones isolated from a mangrove-derived fungus *Penicillium* sp. J Antibiot 74(11):821−824

Bai M, Zheng CJ, Tang DQ, Zhang F, Wang HY, Chen G-Y (2019) Two new secondary metabolites from a mangrove-derived fungus *Cladosporium* sp. JS1-2. J Antibiot 72(10):779-782

Bao J, Zhai H, Zhu K, Yu JH, Zhang Y, Wang Y, Jiang CS, Zhang X, Zhang Y, Zhang H (2018) Bioactive pyridone alkaloids from a deep-sea-derived fungus *Arthrinium* sp. UJNMF0008. Mar Drugs 16(5):174

Barsby T, Kelly MT, Gagne SM, Andersen RJ (2001) Bogorol A produced in culture by a marine *Bacillus* sp. reveals a novel template for cationic peptide antibiotics. Org Lett 3(3):437−440

Bashar MAE, Hamed A, El-Tabakh MAM, Hashem AH, Zaki AA, Al-Askar AA, Abou-Amra ES, El-Beeh ME, Mehany ABM, Shaaban M, Abdelaziz AM, Shaaban KA, Hasaballah AI (2024) Anticancer, antimicrobial, insecticidal and molecular docking of sarcotrocheliol and cholesterol from the marine soft coral *Sarcophyton Trocheliophorum*. Sci Rep 14(1):28028

Bhattacharya D, Lai TK, Saha A, Selvin J, Mukherjee J (2021) Structural elucidation and antimicrobial activity of a diketopiperazine isolated from a *Bacillus* sp. associated with the marine sponge *Spongia officinalis*. Nat Prod Res 35(14):2315−2323

Bitzer J, Grosse T, Wang L, Lang S, Beil W, Zeeck A (2006) New aminophenoxazinones from a marine *Halomonas* sp.: fermentation, structure elucidation, and biological activity. J Antibiot 59(2):86−92

Brana AF, Sarmiento-Vizcaino A, Osset M, Perez-Victoria I, Martin J, Pedro Nd, Cruz Mdl, Diaz C, Vicente F, Reyes F, Garcia LA, Blanco G (2017) Lobophorin K, a new natural product with cytotoxic activity produced by *Streptomyces* sp. M-207 associated with the deep-sea coral *Lophelia pertusa*. Mar Drugs 15(5):144

Brana AF, Sarmiento-Vizcaino A, Perez-Victoria I, Martin J, Otero L, Palacios-Gutierrez JJ, Fernandez J, Mohamedi Y, Fontanil T, Salmon M, Cal S, Reyes F, Garcia LA, Blanco G (2019) Desertomycin G, a new antibiotic with activity against *Mycobacterium tuberculosis* and human breast tumor cell lines produced by *Streptomyces althioticus* MSM3, isolated from the Cantabrian sea intertidal macroalgae *Ulva* sp. Mar Drugs 17(2):114

Brumley D, Spencer KA, Gunasekera SP, Sauvage T, Biggs J, Paul VJ, Luesch H (2018) Isolation and characterization of anaephenes A-C, alkylphenols from a *Filamentous Cyanobacterium* (*Hormoscilla* sp., Oscillatoriales). J Nat Prod 81(12):2716−2721

Buedenbender L, Astone FA, Tasdemir D (2020) Bioactive molecular networking for mapping the antimicrobial constituents of the Baltic brown alga *Fucus vesiculosus*. Mar Drugs 18(6):311

Cai C, Chen Y, Zhou L, Gong N, Zhang H, Sun C, Ma J, Ju J (2023) Antimicrobial polyketides from the marine-derived fungus *Spiromastix* sp. SCSIO F190. J Nat Prod 86(3):589−595

Calcul L, Longeon A, Al-Mourabit A, Guyot M, Bourguet-Kondracki M-L (2003) Novel alkaloids of the aaptamine class from an Indonesian marine sponge of the genus *Xestospongia*. Tetrahedron 59(34):6539−6544

Campana R, Favi G, Baffone W, Lucarini S (2019) Marine alkaloid 2,2-bis(6-bromo-3-indolyl) ethylamine and its synthetic derivatives inhibit microbial biofilms formation and disaggregate developed biofilms. Microorganisms 7(2):28

Campana R, Mangiaterra G, Tiboni M, Frangipani E, Biavasco F, Lucarini S, Citterio B (2020) A fluorinated analogue of marine bisindole alkaloid 2,2-bis(6-bromo-1H-indol-3-yl)ethanamine as potential anti-biofilm agent and antibiotic adjuvant against *Staphylococcus aureus*. Pharmaceuticals 13(9):210

Cao D-T, Nguyen TL, Tran VH, Huong DTM, Quyen VT, Nguyen MA, Le-Thi HM, Chau VM, Pham VC (2019) Synthesis, structure and antimicrobial activity of novel metabolites from a marine actinomycete in Vietnam′s East Sea. Nat Prod Commun 14(1):1934578X1901400132

Cao DT, Tran VH, Vu VN, Mai HDT, Le THM, Vu TQ, Nguyen HH, Chau VM, Pham VC (2019) Antimicrobial metabolites from a marine-derived actinomycete *Streptomyces* sp. G278. Nat Prod Res 33(22):3223−3230

Carballeira NM, Emiliano A, Hernandez-Alonso N, Gonzalez FA (1998) Facile total synthesis and antimicrobial activity of the marine fatty acids (Z)-2-methoxy-5-hexadecenoic acid and (Z)-2-methoxy-6-hexadecenoic acid. J Nat Prod 61(12):1543−1546

Carletti I, Banaigs B, Amade P (2000) Matemone, a new bioactive bromine-containing oxindole alkaloid from the Indian Ocean sponge *Iotrochota purpurea*. J Nat Prod 63(7):981−983

Carretero-Molina D, Ortiz-Lopez FJ, Martin J, Oves-Costales D, Diaz C, de la Cruz M, Cautain B, Vicente F, Genilloud O, Reyes F (2020) New napyradiomycin analogues from *Streptomyces* sp. strain CA-271078. Mar Drugs 18(1):22

Cen S, Jia J, Ge Y, Ma Y, Li X, Wei J, Bai Y, Wu X, Song J, Bi H, Wu B (2021) A new antibacterial 3,5-dimethylorsellinic acid-based meroterpene from the marine fungus *Aspergillus* sp. CSYZ-1. Fitoterapia 152:104908

Chakraborty K, Kizhakkekalam VK, Joy M, Chakraborty RD (2020) Moving away from traditional antibiotic treatment: can macrocyclic lactones from marine macroalga-associated heterotroph be the alternatives? Appl Microbiol Biotechnol 104(16):7117−7130

Chen B, Zhao Q, Gu Y-C, Lan L, Wang C-Y, Guo Y-W (2022) Xishaeleganins A-D, sesquiterpenoid hydroquinones from Xisha marine sponge *Dactylospongia elegans*. Mar Drugs 20(2):118

Chen L, Lin F, Jiang H, Zhao W, Zhou J, Jiang H, Lian Y. (2020a). CN110698537

Chen L, Lin F, Jiang H, Zhao W, Zhou J, Jiang H, Lian Y. (2020b). CN110698541

Chen MH, Lian YY, Fang DS, Chen L, Jia J, Zhang WL, Lin R, Xie Y, Bi HK, Jiang H (2021) Identification and antimicrobial properties of a new alkaloid produced by marine-derived *Verrucosispora* sp. FIM06-0036. Nat Prod Res 35(22):4211−4217

Chen MH, Zhang WL, Chen L, Lin R, Xie Y, Fang DS, Lian YY, Jiang H (2019) Isolation, purification and identification of two new alkaloids metabolites from marine-derived *Verrucosispora* sp. FIM06025. Nat Prod Res 33(20):2897−2903

Chen M, Chai W, Zhu R, Song T, Zhang Z, Lian XY (2018) Streptopyrazinones A-D, rare metabolites from marine-derived *Streptomyces* sp. ZZ446. Tetrahedron 74(16):2100−2106

Chen M, Shen NX, Chen ZQ, Zhang FM, Chen Y (2017) Penicilones A-D, anti-MRSA azaphilones from the marine-derived fungus *Penicillium janthinellum* HK1-6. J Nat Prod 80(4):1081−1086

Chen M, Yan Y, Ge H, Jiao W-H, Zhang Z, Lin H-W (2020) Pseudoceroximes A-E and pseudocerolides A-E - bromotyrosine derivatives from a *Pseudoceratina* sp. marine sponge collected in the South China Sea. Eur J Org Chem 2020(17):2583−2591

Chen SC, Liu ZM, Tan HB, Chen YC, Li SN, Li HH, Guo H, Zhu S, Liu HX, Zhang WM (2019) Tersone A-G, new pyridone alkaloids from the deep-sea fungus *Phomopsis tersa*. Mar Drugs 17(7):394

Chen S, Zhang D, Chen M, Zhang Z, Lian XY (2020) A rare diketopiperazine glycoside from marine-sourced *Streptomyces* sp. ZZ446. Nat Prod Res 34(7):1046−1050

Chen W, Chen C, Long J, Lan S, Lin X, Liao S, Yang B, Zhou X, Wang J, Liu Y (2021) Bioactive secondary metabolites from the deep-sea derived fungus *Aspergillus* sp. SCSIO 41029. J Antibiot 74(2):156−159

Chen Y, Tang J, Tang X, Wang C, Lian Y, Shao Z, Yao X, Gao H (2013) New phenethylamine derivatives from *Arenibacter nanhaiticus* sp. nov. NH36AT and their antimicrobial activity. J Antibiot 66(11):655−661

Chen ZQ, Liu PP, Wang Y, Zhu WM (2013) Antibacterial polyketides of *Aspergillus terreus* OUCMDZ-1925 from Yellow River delta. Junwu Xuebao 32(2):277−285

Chen Z, Ou P, Liu L, Jin X (2020) Anti-MRSA activity of actinomycin X2 and collismycin A produced by *Streptomyces globisporus* WA5-2-37 from the intestinal tract of American Cockroach (Periplaneta americana). Front Microbiol 11:555

Chen Z, She J, Liu J, Peng B, Yuan Z, Zhou X (2024) Pyripyropene U, a new alkaloid from the sponge-derived fungus *Aspergillus* sp. SCSIO41420. Nat Prod Res 39(7):1939−1944

Cheng X, Liang X, Yao FH, Liu XB, Qi SH (2021) Fusidane-type antibiotics from the marine-derived fungus *Simplicillium* sp. SCSIO 41513. J Nat Prod 84(11):2945−2952

Cheng YB, Jensen PR, Fenical W (2013) Cytotoxic and antimicrobial napyradiomycins from two marine-derived streptomyces strains. Eur J Org Chem 2013(18):3751−3757

Cheng Z, Xu W, Liu L, Li S, Yuan W, Luo Z, Zhang J, Cheng Y, Li Q (2018) Peniginsengins B-E, new farnesylcyclohexenones from the deep sea-derived fungus *Penicillium* sp. YPGA11. Mar Drugs 16(10):358

Chi LP, Liu D, Li XM, Wan Y, Wang BG, Li X (2023) Aspertides A-E: antimicrobial pentadepsipeptides with a unique p-methoxycinnamoyl amide group from the marineisolates *Aspergillus tamarii* MA-21 and *Aspergillus insuetus* SD-512. J Agric Food Chem 71(36):13316−13324

Chianese G, Esposito FP, Parrot D, Ingham C, de Pascale D, Tasdemir D (2018) Linear aminolipids with moderate antimicrobial activity from the antarctic gram-negative bacterium *Aequorivita* sp. Mar

Drugs 16(6):187

Chingizova EA, Yurchenko EA, Chingizov AR, Klimovich AA, Pislyagin EA, Menchinskaya ES, Kuzmich AS, Trinh PTH, Ngoc NTD, Van TTT, Guzhova IV, Aminin DL, Yurchenko AN (2024) The effects of marine fungal asterripeptides A-C on *in vitro* and *in vivo Staphylococcus aureus* skin infection. Pharmaceuticals 17(10):1345

Cho E, Kwon OS, Chung B, Lee J, Sun J, Shin J, Oh KB (2020) Antibacterial activity of chromomycins from a marine-derived *Streptomyces microflavus*. Mar Drugs 18(10):522

Chung B, Kwon OS, Shin J, Oh KB (2021) Antibacterial activity and mode of action of lactoquinomycin A from *Streptomyces bacillaris*. Mar Drugs 19(1):7

Cochereau B, Robiou du Pont T, Pouchus YF, Tasdemir D, Meslet-Cladiere L, Roullier C (2023) Enzymatic bromination of marine fungal extracts for enhancement of chemical diversity. Biocatal Agric Biotechnol 51:102786

Cui H, Li F, Yan B, Shaaben K, Qin S, Laatsch H (2006) *Streptomyces* sp. M095 from Jiaozhou bay producing inhibitory-fungal antibiotic, holomycin. Zhongguo Haiyang Yaowu 25(1):11−15

Cui H, Mei W, Han Z, Wu J, Lin H, Hong K, Dai H (2008) Antibacterial metabolites from fermentation broth of marine fungus 095407. Zhongguo Yaowu Huaxue Zazhi 18(2):131−134

da Silva AB, Silveira ER, Wilke DV, Ferreira EG, Costa-Lotufo LV, Torres MCM, Ayala AP, Costa WS, Canuto KM, de Araujo-Nobre AR, Araujo AJ, Filho JDBM, Pessoa ODL (2019) Antibacterial salinaphthoquinones from a strain of the bacterium *Salinispora arenicola* recovered from the marine sediments of St. Peter and St. Paul Archipelago, Brazil. J Nat Prod 82(7):1831−1838

Dai LT, Yang L, Kong FD, Ma QY, Xie QY, Dai HF, Yu ZF, Zhao YX (2021) Cytotoxic indole-diterpenoids from the marine-derived fungus *Penicillium* sp. KFD28. Mar Drugs 19(11):613

De BC, Zhang W, Zhang G, Liu Z, Tan B, Zhang Q, Zhang L, Zhang H, Zhu Y, Zhang C (2021) Host-dependent heterologous expression of berninamycin gene cluster leads to linear thiopeptide antibiotics. Org Biomol Chem 19(41):8940−8946

De Silva ED, Scheuer PJ (1980) Manoalide, an antibiotic sesterterpenoid from the marine sponge *Luffariella variabilis* (Polejaeff). Tetrahedron Lett 21(17):1611

De Silva ED, Scheuer PJ (1981) Three new sesterterpenoid antibiotics from the marine sponge *Luffariella variabilis* (Polejaff). Tetrahedron Lett 22(33):3147

Desbois AP, Lebl T, Yan L, Smith VJ (2008) Isolation and structural characterisation of two antibacterial free fatty acids from the marine diatom, *Phaeodactylum tricornutum*. Appl Microbiol Biotechnol 81(4):755−764

Desbois AP, Mearns-Spragg A, Smith VJ (2009) A fatty acid from the diatom *Phaeodactylum tricornutum* is antibacterial against diverse bacteria including multi-resistant *Staphylococcus aureus* (MRSA). Mar Biotechnol 11(1):45−52

Ding LJ, Gu BB, Jiao WH, Yuan W, Li YX, Tang WZ, Yu HB, Liao XJ, Han BN, Li ZY, Xu SH, Lin HW (2015) New furan and cyclopentenone derivatives from the sponge-associated fungus *Hypocrea koningii* PF04. Mar Drugs 13(9):5579−5592

Ding L, Ren L, Li S, Song J, Han Z, He S, Xu S (2019) Production of new antibacterial 4-hydroxy-α-pyrones by a marine fungus *Aspergillus niger* cultivated in solid medium. Mar Drugs 17(6):344

Djinni I, Defant A, Kecha M, Mancini I (2013) Antibacterial polyketides from the marine alga-derived endophytic *Streptomyces sundarbansensis*: a study on hydroxypyrone tautomerism. Mar Drugs 11:124−135

Dong Y, Ding W, Sun C, Ji X, Ling C, Zhou Z, Chen Z, Chen X, Ju J (2020) Julichrome monomers from

marine gastropod mollusk-associated *Streptomyces* and stereochemical revision of julichromes Q3 · 5 and Q3 · 3. Chem Biodiversity 17(4):e2000057

Du FY, Li XM, Li CS, Shang Z, Wang BG (2012) Cristatumins A-D, new indole alkaloids from the marine-derived endophytic fungus *Eurotium cristatum* EN-220. Bioorg Med Chem Lett 22(14):4650−4653

Du FY, Li XM, Li CS, Wang BG (2012) Chemical constituents of *Aspergillus sydowii* EN-198, an endophytic fungus derived from the marine-mangrove plant Hibiscus tiliaceus. Haiyang Kexue 36(12):6−11

Duan C, Wang S, Huo R, Li E, Wang M, Ren J, Pan Y, Liu L, Liu G (2022) Sorbicillinoid derivatives with the radical scavenging activities from the marine-derived fungus *Acremonium chrysogenum* C10. J Fungi 8(5):530

Edrada RA, Proksch P, Wray V, Christ R, Witte L, Van Soest RWM (1996) Bioactive isoquinoline quinone from an undescribed Philippine marine sponge of the genus *Xestospongia*. J Nat Prod 59(10):973−976

Edrada RA, Proksch P, Wray V, Witte L, Mueller WEG, Van Soest RWM (1996) Four new bioactive manzamine-type alkaloids from the Philippine marine sponge *Xestospongia ashmorica*. J Nat Prod 59(11):1056−1060

El-Gendy MMA, Hawas UW, Jaspars M (2008) Novel bioactive metabolites from a marine derived bacterium *Nocardia* sp. ALAA 2000. J Antibiot 61(6):379−386

El-Gendy MMAA, Mohamed ZK, Hekal NZ, Ali FM, Yousef AEM (2018) Production of bioactive metabolites from different marine endophytic *Streptomyces* species and testing them against methicillin-resistant *Staphylococcus aureus* (MRSA) and cancer cell lines. BioTechnologia 99(1):13−35

El-Hawary SS, Sayed AM, Mohammed R, Hassan HM, Rateb ME, Amin E, Mohammed TA, El-Mesery M, Bin Muhsinah A, Alsayari A, Wajant H, Anany MA, Abdelmohsen UR (2019) Bioactive brominated oxindole alkaloids from the red sea sponge *Callyspongia siphonella*. Mar Drugs 17(8):465

El-Sayed MH, Awad HM (2013) Isolation and structure elucidation of helalomycin-1, a new antitumor antibiotic produced by a new marine bacterium *Streptomyces* sp. strain HuGu-11. J Appl Sci Res 9(3):1831−1844

El Sayed KA, Yousaf M, Hamann MT, Avery MA, Kelly M, Wipf P (2002) Microbial and chemical transformation studies of the bioactive marine sesquiterpenes (S)-(+)-curcuphenol and -curcudiol isolated from a deep reef collection of the Jamaican sponge Didiscus oxeata. J Nat Prod 65(11):1547−1553

Ellis GA, Wyche TP, Fry CG, Braun DR, Bugni TS (2014) Solwaric acids A and B, antibacterial aromatic acids from a marine *Solwaraspora* sp. Mar Drugs 12(2):1013−1022

Elsayed Y, Refaat J, Abdelmohsen UR, Ahmed S, Fouad MA (2017) Rhodozepinone, a new antitrypanosomal azepino-diindole alkaloid from the marine sponge-derived bacterium *Rhodococcus* sp. UA13. Med Chem Res 26(11):2751−2760

Elsebai MF, Ghabbour HA, Legrave N, Fontaine-Vive F, Mehiri M (2018) New bioactive chlorinated cyclopentene derivatives from the marine-derived fungus *Phoma* sp. Med Chem Res 27(8):1885−1892

Elsebai MF, Kehraus S, Lindequist U, Sasse F, Shaaban S, Guetschow M, Josten M, Sahl H-G, Koenig GM (2011) Antimicrobial phenalenone derivatives from the marine-derived fungus

Coniothyrium cereale. Org Biomol Chem 9(3):802−808

Elsebai MF, Natesan L, Kehraus S, Mohamed IE, Schnakenburg G, Sasse F, Shaaban S, Gutschow M, Konig GM (2011) HLE-inhibitory alkaloids with a polyketide skeleton from the marine-derived fungus *Coniothyrium cereale*. J Nat Prod 74(10):2282−2285

Elshamy AI, Mohamed TA, Elkady EM, Saleh IA, El-Beih AA, Alhammady MA, Ohta S, Umeyama A, Pare PW, Hegazy M-EF (2021) Paralemnolins X and Y, new antimicrobial sesquiterpenoids from the soft coral *Paralemnalia thyrsoide*. Antibiotics (Basel, Switz.) 10(10):1158

Eltamany EE, Abdelmohsen UR, Ibrahim AK, Hassanean HA, Hentschel U, Ahmed SA (2014) New antibacterial xanthone from the marine sponge-derived *Micrococcus* sp. EG45. Bioorg Med Chem Lett 24(21):4939−4942

Erdogan I, Sener B (2000) Antibacterial screening of terpenoids from some marine sponges. J Fac Pharm Gazi Univ 17(1):1−6

Etahiri S, El Kouri AK, Bultel-Ponce V, Guyot M, Assobhei O (2007) Antibacterial bromophenol from the marine red alga *Pterosiphonia complanata*. Nat Prod Commun 2(7):749−752

Fahy E, Andersen RJ, He CH, Clardy J (1985) Garveatin A, an antimicrobial 1(4H)-anthracenone derivative from the hydroid *Garveia annulata*. J Org Chem 50(7):1149

Fakee J, Bolton JJ, Le Roes-Hill M, Durrell KA, Antunes E, Beukes DR (2023) Antimicrobial activity of the secondary metabolites isolated from a South African red seaweed, *Laurencia corymbosa*. Molecules 28(5):2063

Fang C, Zhang Q, Zhu Y, Zhang L, Zhang W, Ma L, Zhang H, Zhang C (2020) Proximicins F and G and diproximicin A: aminofurans from the marine-derived *Verrucosispora* sp. SCSIO 40062 by overexpression of PPtase genes. J Nat Prod 83(4):1152−1156

Farouk BH, Abdel-Tawab AM, Mostafa ME, El-Ablack FZ, Hamed ESAE, Ayyad S-EN (2024) New bioactive mono-acyl glycerol ester from the Red Sea marine coral *Sclerophytum levi*. Nat Prod Res 26:1−7

Feher D, Barlow R, McAtee J, Hemscheidt TK (2010) Highly brominated antimicrobial metabolites from a marine *Pseudoalteromonas* sp. J Nat Prod 73(11):1963−1966

Fernando AL, Hulangamuwa W, Rafferty R. (2022). Synthetic efforts towards brocazine family of natural products and proposed stereochemical structure activity relationship studies. Abstracts, Midwest Regional Meeting of the American Chemical Society, MWRM-27

Gao SS, Li XM, Zhang Y, Li CS, Wang BG (2011) Conidiogenones H and I, two new diterpenes of cyclopiane class from a marine-derived endophytic fungus *Penicillium chrysogenum* QEN-24S. Chem Biodiversity 8(9):1748−1753

Gerard JM, Haden P, Kelly MT, Andersen RJ (1999) Loloatins A-D, cyclic decapeptide antibiotics produced in culture by a tropical marine bacterium. J Nat Prod 62(1):80−85

Ghandourah M (2025) Pluramycinone metabolites from *Streptomyces* sp. SA0215, isolated from the Saudi Red Sea sediments with antimicrobial and cytotoxic activity. Appl Biochem Microbiol 61(1): 110−116

Gnanamani A, Charyulu EM, Sekaran G, Mandal AB. (2012). IN2011DE01578.

Gorajana A, Kurada BVVSN, Peela S, Jangam P, Vinjamuri S, Poluri E, Zeeck A (2005) 1-Hydroxy-1-norresistomycin, a new cytotoxic compound from a marine actinomycete, *Streptomyces chibaensis* AUBN1/7. J Antibiot 58(8):526−529

Gunasekaran M, Thangavel S, Subramani K (2013) Structure elucidation and biological activities of 1-(3-bromo-5-methylphenyl)-1H-indole from marine *Streptomyces* sp. LCJ85. J Pharma Res

2(11):6

Guo C, Lin XP, Liao SR, Yang B, Zhou XF, Yang XW, Tian XP, Wang JF, Liu YH (2020) Two new aromatic polyketides from a deep-sea fungus *Penicillium* sp. SCSIO 06720. Nat Prod Res 34(9):1197−1205

Guo C, Wang P, Pang X, Lin X, Liao S, Yang B, Zhou X, Wang J, Liu Y (2021) Discovery of a dimeric zinc complex and five cyclopentenone derivatives from the sponge-associated fungus *Aspergillus ochraceopetaliformis*. ACS Omega 6(13):8942−8949

Guo F, Xiao F, Song H, Li X, Xiao Y, Qin Y, Lei X (2024) An optimized marinopyrrole A derivative targets 6-phosphoglucosamine synthetase to inhibit methicillin-resistant *Staphylococcus aureus*. ACS Cent Sci 10(11):2090−2098

Guo L, Yang Q, Wang G, Zhang S, Liu M, Pan X, Pescitelli G, Xie Z (2021) Ring D-modified and highly reduced angucyclinones from marine sediment-derived *Streptomyces* sp. Front Chem (Lausanne, Switz.) 9:756962

Guo W, Zhang Z, Zhu T, Gu Q, Li D (2015) Penicyclones A-E, antibacterial polyketides from the deep sea-derived fungus *Penicillium* sp. F23-2. J Nat Prod 78(11):2699−2703

Ha Y, Zhou Y, Ma M, Wang N, Wang P, Zhang Z (2024) Antimicrobial metabolites from the marine-derived fungus *Aspergillus* sp. ZZ1861. Phytochemistry (Elsevier) 224:114164

Hadjkacem F, Elleuch J, Pierre G, Fendri I, Michaud P, Abdelkafi S (2024) Production and purification of fucoxanthins and βcarotenes from *Halopteris scoparia* and their effects on digestive enzymes and harmful bacteria. Environ Technol 45(15):2923−2934

Hamed A, Abdel-Razek AS, Araby M, Abu-Elghait M, El-Hosari DG, Frese M, Soliman HSM, Stammler HG, Sewald N, Shaaban M (2021) Meleagrin from marine fungus *Emericella dentata* NQ45: crystal structure and diverse biological activity studies. Nat Prod Res 35(21):3830−3838

Han J, Liu M, Jenkins ID, Liu X, Zhang L, Quinn RJ, Feng Y (2020) Genome-inspired chemical exploration of marine fungus *Aspergillus fumigatus* MF071. Mar Drugs 18(7):352

Han L, Xu N, Shi J, Yan X, Zeng C (2005) Isolation and pharmacological activities of bromophenols from *Rhodomela confervoides*. Chin J Oceanol Limnol 23(2):226−229

Han WJ, Lu XLXu QZ, Liu XN, Jiao BH (2008) Isolation, identification and biological characterization of secondary metabolites produced by a marine *Bacillus subtilis*. Dier Junyi Daxue Xuebao 29(10):1234−1238

Han X, Wang H, Li B, Chen X, Li T, Yan X, Ouyang H, Lin W, He S (2024) New diterpenes and diterpene glycosides with antibacterial activity from soft coral *Lemnalia bournei*. Mar Drugs 22(4):157

Han Z, Xu Y, McConnell O, Liu L, Li Y, Qi S, Huang X, Qian P (2012) Two antimycin A analogues from marine-derived actinomycete *Streptomyces lusitanus*. Mar Drugs 10:668−676

Handayani D, Amilia R, Aminah I, Hertiani T, Ariantari NP, Proksch P (2020) Bioactive compounds from a marine sponge derived fungus *Penicillium oxalicum* WR3. Rasayan J Chem 13(3):1817−1823

Handayani D, Aminah I, Pontana Putra P, Eka Putra A, Arbain D, Satriawan H, Efdi M, Celik I, Ekawati Tallei T (2023) The depsidones from marine sponge-derived fungus *Aspergillus unguis* IB151 as an anti-MRSA agent: Molecular docking, pharmacokinetics analysis, and molecular dynamic simulation studies. Saudi Pharm J 31(9):101744

Handayani D, Dwinatrana K, Rustini R (2022) Antibacterial compound from marine sponge derived fungus *Aspergillus sydowii* DC08. Rasayan J Chem 15(4):2485−2492

Handayani D, Rendowati A, Aminah I, Ariantari NP, Proksch P (2020) Bioactive compounds from marine sponge derived fungus *Aspergillus unguis* WR8. Rasayan J Chem 13(4):2633−2638

Hanif N, Ardan MS, Tohir D, Setiawan A, de Voogd NJ, Farid M, Murni A, Tanaka J (2019) Polybrominated diphenyl ethers with broad spectrum antibacterial activity from the Indonesian marine sponge *Lamellodysidea herbacea*. J Appl Pharm Sci 9(12):1−6

Harizani M, Diakaki D-I, Perdikaris S, Roussis V, Ioannou E (2022) New C15 acetogenins from two species of *Laurencia* from the Aegean Sea. Molecules 27(6):1866

He S, Li P, Wang J, Zhang Y, Lu H, Shi L, Huang T, Zhang W, Ding L, He S, Liu L (2022) Discovery of new secondary metabolites from marine bacteria *Hahella* based on an omics strategy. Mar Drugs 20(4):269

Hong L-L, Sun J-B, Yang F, Liu M, Tang J, Sun F, Jiao W-H, Wang S-P, Zhang W, Lin H-W (2017) New diterpene alkaloids from the marine sponge *Agelas mauritiana*. RSC Adv 7(39):23970−23976

Hong X, Guan X, Lai Q, Yu D, Chen Z, Fu X, Zhang B, Chen C, Shao Z, Xia J, Qin J-J, Wang W (2022) Characterization of a bioactive meroterpenoid isolated from the marine-derived fungus *Talaromyces* sp. Appl Microbiol Biotechnol 106(8):2927−2935

Hong X, Tang L, Chen Z, Lai Q, Zhang B, Jiang Y, Wang X, He R, Lin J, Shao Z, Lin S, Wang W (2024) Benzoquinone and furopyridinone derivatives from the marine-derived fungus *Talaromyces* sp. MCCC 3A01752. Nat Prod Res 38(2):320−326

Hu J, Li Z, Gao J, He H, Dai H, Xia X, Liu C, Zhang L, Song F (2019) New diketopiperazines from a marine-derived fungus strain *Aspergillus versicolor* MF180151. Mar Drugs 17(5):262

Hu Z, Qin L, Wang Q, Ding W, Chen Z, Ma Z (2016) Angucycline antibiotics and its derivatives from marine-derived actinomycete *Streptomyces* sp. A6H. Nat Prod Res 30(22):2551−2558

Huang G, Wang Q, Wen H, Li J, He S, Wang X, Ding L (2024) Antibiofilm efficacy and mechanism of the marine chlorinated indole sesquiterpene against methicillin-resistant *Staphylococcus aureus*. Foodborne Pathog Dis 21(8):491−498

Huang P, Xie F, Ren B, Wang Q, Wang J, Wang Q, Abdel-Mageed WM, Liu M, Han J, Oyeleye A, Shen J, Song F, Dai H, Liu X, Zhang L (2016) Anti-MRSA and anti-TB metabolites from marine-derived *Verrucosispora* sp. MS100047. Appl Microbiol Biotechnol 100(17):7437−7447

Huang R, Ma S, Wu J, Riaz A, Song L, Zhou S, Zhou R, Qiu J, He J (2024) A new tetrahydroxybenzophenone from a marine-associated fungus of *Penicillium* sp. and its potential antibacterial activity. Nat Prod Res 16:1−8

Huang X, Cai J, Xiao J, Zhou X, Liu Y, Li Y, Yang B (2024) Antimicrobial isochroman-derived atropo-diastereomeric dimers from *Penicillium Steckii* SCISO 41228. Chem Biodiversity 22(2):e202402152

Huang Z, Liu D, Chen S, Ren J, Gao C, Li Z, Fan A, Lin W (2024) Brominated depsidones with antibacterial effects from a deep-sea-derived fungus *Spiromastix* sp. Mar Drugs 22(2):78

Hughes CC, Kauffman CA, Jensen PR, Fenical W (2010) Structures, reactivities, and antibiotic properties of the marinopyrroles A-F. J Org Chem 75(10):3240−3250

Hughes CC, Prieto-Davo A, Jensen PR, Fenical W (2008) The Marinopyrroles, antibiotics of an unprecedented structure class from a marine *Streptomyces* sp. Org Lett 10(4):629−631

Imhoff JF, Sun M, Wiese J, Tank M, Zeeck A (2018) First evidence of dehydroabietic acid production by a marine phototrophic gammaproteobacterium, the purple sulfur bacterium *Allochromatium vinosum* MT86. Mar Drugs 16(8):270

Iniyan AM, Sudarman E, Wink J, Kannan RR, Vincent SGP (2019) Ala-geninthiocin, a new broad spectrum thiopeptide antibiotic, produced by a marine *Streptomyces* sp. ICN19. J Antibiot 72(2):99−105

Inprasit T, Motina K, Pisitsak P, Chitichotpanya P (2018) Dyeability and antibacterial finishing of hemp fabric using natural bioactive neem extract. Fibers Polym 19(10):2121−2126

Inukai M, Isono F, Shiozawa H, Takahashi H, Haruyama H, Kodama K, Sakaida Y. (1993). JP05078355.

Isnansetyo A, Cui L, Hiramatsu K, Kamei Y (2003) Antibacterial activity of 2,4-diacetylphloroglucinol produced by *Pseudomonas* sp. AMSN isolated from a marine alga, against vancomycin-resistant *Staphylococcus aureus*. Int J Antimicrob Agents 22(5):545−547

Isnansetyo A, Horikawa M, Kamei Y (2001) In vitro anti-methicillin-resistant *Staphylococcus aureus* activity of 2,4-diacetylphloroglucinol produced by *Pseudomonas* sp. AMSN isolated from a marine alga. J Antimicrob Chemother 47(5):724−725

Isnansetyo A, Kamei Y (2003) MC21-A, a bactericidal antibiotic produced by a new marine bacterium, *Pseudoalteromonas phenolica* sp. nov. O-BC30T, against methicillin-resistant *Staphylococcus aureus*. Antimicrob Agents Chemother 47(2):480−488

Isnansetyo A, Kamei Y (2009) Anti-methicillin-resistant *Staphylococcus aureus* (MRSA) activity of MC21-B, an antibacterial compound produced by the marine bacterium *Pseudoalteromonas phenolica* O-BC30T. Int J Antimicrob Agents 34(2):131−135

Jadulco R, Brauers G, Edrada RA, Ebel R, Wray V, Sudarsono, Proksch P (2002) New metabolites from sponge-derived fungi *Curvularia lunata* and *Cladosporium herbarum*. J Nat Prod 65(5):730−733

Jang JH, Kanoh K, Adachi K, Shizuri Y (2006) New dihydrobenzofuran derivative, awajanoran, from marine-derived *Acremonium* sp. AWA16-1. J Antibiot 59(7):428−431

Jang KH, Chung SC, Shin J, Lee SH, Kim TI, Lee HS, Oh KB (2007) Aaptamines as sortase A inhibitors from the tropical sponge *Aaptos aaptos*. Bioorg Med Chem Lett 17(19):5366-5369

Jenssen M, Rainsford P, Juskewitz E, Andersen JH, Hansen EH, Isaksson J, Rämä T, Hansen KØ (2021) Lulworthinone, a new dimeric naphthopyrone from a marine fungus in the family *Lulworthiaceae* with antibacterial activity against clinical methicillin-resistant *Staphylococcus aureus* isolates. Front Microbiol 12:730740

Jiang L, Teng B, Zhang M, Chen S, Zhang D, Zhai L, Lin J, Lei H (2024) Pestalotiopols E-J, six new polyketide derivatives from a marine derived fungus *Pestalotiopsis* sp. SWMU-WZ04-1. Mar Drugs 22(1):15

Jiang S, Zhang L, Pei X, Deng F, Hu D, Chen G, Wang C, Hong K, Yao X, Gao H (2017) Chalcomycins from marine-derived *Streptomyces* sp. and their antimicrobial activities. Mar Drugs 15(6):153

Jiang W, Zhang Z, Shan T, Miao L, Zhou X. (2018). CN108485987

Jiang YJ, Zhang DS, Zhang HJ, Li JQ, Ding WJ, Xu CD, Ma ZJ (2018) Medermycin-type naphthoquinones from the marine-derived *Streptomyces* sp. XMA39. J Nat Prod 81(9):2120−2124

Jiang Y, Wang J, Zhang H, Tian X, Liang Z, Xu X, Bao J, Chen B (2024) Biological activity and sterilization mechanism of marine fungi-derived aromatic butenolide asperbutenolide A against *Staphylococcus aureus*. Chem Biodiversity 21(2):e202301826

Jiang Z, Boyd KG, Mearns-Spragg A, Adams DR, Wright PC, Burgess JG (2000) Two diketopiperazines and one halogenated phenol from cultures of the marine bacterium, *Pseudoalteromonas luteoviolacea*. Nat Prod Lett 14(6):435−440

Jiao WH, Li J, Liu Q, Xu TT, Shi GH, Yu HB, Yang F, Han BN, Li M, Lin HW (2014) Dysidinoid A, an unusual meroterpenoid with anti-MRSA activity from the South China sea sponge *Dysidea* sp. Molecules 19(11):18025

Kaleem S, Ge H, Yi W, Zhang Z, Wu B (2021) Isolation, structural elucidation, and antimicrobial evaluation of the metabolites from a marine-derived fungus *Penicillium* sp. ZZ1283. Nat Prod Res 35(15):2498−2506

Kaleem S, Qin L, Yi W, Lian X-Y, Zhang Z (2020) Bioactive metabolites from the mariana trench sediment-derived fungus *Penicillium* sp. SY2107. Mar Drugs 18(5):258

Kalinovskaya NI, Romanenko LA, Kalinovsky AI (2017) Antibacterial low-molecular-weight compounds produced by the marine bacterium *Rheinheimera japonica* KMM 9513T. Antonie Van Leeuwenhoek 110(5):719−726

Kamei Y, Isnansetyo A (2003) Lysis of methicillin-resistant Staphylococcus aureus by 2,4-diacetylphloroglucinol produced by *Pseudomonas* sp. AMSN isolated from a marine alga. Int J Antimicrob Agents 21(1):71−74

Kanoh K, Adachi K, Matsuda S, Shizuri Y, Yasumoto K, Kusumi T, Okumura K, Kirikae T (2008) New sulfoalkylresorcinol from marine-derived fungus, *Zygosporium* sp. KNC52. J Antibiot 61(3):192−194

Kanoh K, Okada A, Adachi K, Imagawa H, Nishizawa M, Matsuda S, Shizuri Y, Utsumi R (2008) Ascochytatin, a novel bioactive spirodioxynaphthalene metabolite produced by the marine-derived fungus, *Ascochyta* sp. NGB4. J Antibiot 61(3):142−148

Katif C, Chilczuk T, Sabour B, Belattmania Z, Hilmi A, Niedermeyer THJ, Barakate M (2022) Isolation and structure elucidation of desferrioxamine b and the new desferrioxamine B2 antibiotics from a brown marine macroalga *Carpodesmia tamariscifolia* associated *Streptomyces* isolate. Biointerface Res Appl Chem 12(4):5647−5662

Kato Y, Fusetani N, Matsunaga S, Hashimoto K (1985) Bioactive marine metabolites. XI. Spongionellin and dehydrospongionellin, new furanosesterterpenes which inhibit cell division of fertilized starfish eggs, from the marine sponge *Spongionella* sp. Chem Lett 14(10):1521−1524

Keffer JL, Plaza A, Bewley CA (2009) Motualevic acids A-F, antimicrobial acids from the aponge *Siliquariaspongia* sp. Org Lett 11(5):1087−1090

Khamthong N, Rukachaisirikul V, Phongpaichit S, Preedanon S, Sakayaroj J (2012) Bioactive polyketides from the sea fan-derived fungus *Penicillium citrinum* PSU-F51. Tetrahedron 68(39):8245−8250

Khamthong N, Rukachaisirikul V, Phongpaichit S, Preedanon S, Sakayaroj J (2014) An antibacterial cytochalasin derivative from the marine-derived fungus *Diaporthaceae* sp. PSU-SP2/4. Phytochem Lett 10:5−9

Khamthong N, Rukachaisirikul V, Tadpetch K, Kaewpet M, Phongpaichit S, Preedanon S, Sakayaroj J (2012) Tetrahydroanthraquinone and xanthone derivatives from the marine-derived fungus *Trichoderma aureoviride* PSU-F95. Arch Pharmacal Res 35(3):461−468

Khan FA, Ahmad S, Kodipelli N, Shivange G, Anindya R (2014) Syntheses of a library of molecules on the marine natural product ianthelliformisamines platform and their biological evaluation. Org Biomol Chem 12(23):3847−3865

Khatri Chhetri B, Lavoie S, Sweeney-Jones AM, Mojib N, Raghavan V, Gagaring K, Dale B, McNamara CW, Soapi K, Quave CL, Polavarapu PL, Kubanek J (2019) Peyssonnosides A-B, unusual diterpene glycosides with a sterically encumbered cyclopropane motif: Structure elucidation using an integrated spectroscopic and computational workflow. J Org Chem 84(13):8531−8541

Ki D-W, Awouafack MD, Wong CP, Nguyen HM, Thai QM, Nu LHT, Morita H (2019) Brominated diphenyl ethers including a new tribromoiododiphenyl ether from the Vietnamese marine

sponge *Arenosclera* sp. and their antibacterial activities. Chem Biodiversity 16(3):e1800593

Kim M-Y, Sohn JH, Ahn JS, Oh H (2009) Alternaramide, a cyclic depsipeptide from the marine-derived fungus *Alternaria* sp. SF-5016. J Nat Prod 72(11):2065−2068

Kim MC, Cullum R, Hebishy AMS, Mohamed HA, Faraag AHI, Salah NM, Abdelfattah MS, Fenical W (2020) Mersaquinone, a new tetracene derivative from the marine-derived *Streptomyces* sp. EG1 exhibiting activity against methicillin-resistant *Staphylococcus aureus* (MRSA). Antibiotics (Basel, Switz.) 9(5):252

Kim MC, Li Z, Cullum R, Molinski TF, Eid MAG, Hebishy AMS, Faraag AHI, Abdel Moneim AE, Abdelfattah MS, Fenical W (2022) Chlororesistoflavins A and B, chlorinated benzopyrene antibiotics produced by the marine-derived actinomycete *Streptomyces* sp. strain EG32. J Nat Prod 85(1):270−275

Kim S, Hillman PF, Lee JY, Lee J, Lee J, Cha SS, Oh D-C, Nam SJ, Fenical W (2022) Actinopolymorphols E and F, pyrazine alkaloids from a marine sediment-derived bacterium *Streptomyces* sp. J Antibiot 75(11):619−625

Kim S, Le TC, Han SA, Hillman PF, Hong A, Hwang S, Du YE, Kim H, Oh DC, Cha SS, Lee J, Nam S-J, Fenical W (2022) Saccharobisindole, neoasterric nethyl ester, and 7-chloro-4(1H)-quinolone: three new compounds isolated from the marine bacterium *Saccharomonospora* sp. Mar Drugs 20(1):35

Kim YG, Lee JH, Lee S, Lee YK, Hwang BS, Lee J (2021) Antibiofilm activity of phorbaketals from the marine sponge *Phorbas* sp. against *Staphylococcus aureus*. Mar Drugs 19(6):301

Kizhakkekalam VK, Chakraborty K, Joy M (2020) Oxygenated elansolid-type of polyketide spanned macrolides from a marine heterotrophic *Bacillus* as prospective antimicrobial agents against multidrug-resistant pathogens. Int J Antimicrob Agents 55(3):105892

Kizhakkepatt Kizhakkekalam V, Chakraborty K, Joy M (2020) Antibacterial and antioxidant aryl-enclosed macrocyclic polyketide from intertidal macroalgae associated heterotrophic bacterium *Shewanella algae*. Med Chem Res 29(1):145−155

Kokkini M, Oves-Costales D, Sanchez P, Melguizo A, Mackenzie TA, Perez-Bonilla M, Martin J, Giusti A, de Witte P, Vicente F, Genilloud O, Reyes F (2023) New phocoenamicin and maklamicin analogs from cultures of three marine-derived *Micromonospora* strains. Mar Drugs 21(8):443

Kong FD, Ma QY, Huang SZ, Wang P, Wang JF, Zhou LM, Yuan JZ, Dai HF, Zhao YX (2017) Chrodrimanins K-N and related meroterpenoids from the fungus *Penicillium* sp. SCS-KFD09 Isolated from a marine worm, *Sipunculus nudus*. J Nat Prod 80(4):1039−1047

Kontiza I, Stavri M, Zloh M, Vagias C, Gibbons S, Roussis V (2008) New metabolites with antibacterial activity from the marine angiosperm *Cymodocea nodosa*. Tetrahedron 64(8):1696−702

Kossuga MH, de Lira SP, Nascimento AM, Gambardella MTP, Berlinck RGS, Torres YR, Nascimento GGF, Pimenta EF, Silva M, Thiemann OH, Olivia G, Tempone AG, Melhem MSC, de Souza AO, Galetti FCS, Silva CL, Cavalcanti B, Pessoa CO, Moraes MO, Hadju E, Peixinho S, Rocha RM (2007) Isolation and biological activities of secondary metabolites from the sponges *Monanchora arbuscula*, *Aplysina* sp., *Petromica ciocalyptoides* and *Topsentia ophiraphidites*, from the ascidian *Didemnum ligulum* and from the octocoral *Carijoa riisei*. Quim Nova 30(5):1194−1202

Kubota T, Kamijyo Y, Takahashi-Nakaguchi A, Fromont J, Gonoi T, Kobayashi J-i (2013) Zamamiphidin A, a new manzamine related alkaloid from an Okinawan marine sponge *Amphimedon* sp. Org Lett 15(3):610−612

Kumar R, Subramani R, Aalbersberg W (2013) Three bioactive sesquiterpene quinones from the Fijian marine sponge of the genus *Hippospongia*. Nat Prod Res 27(16):1488−1491

Kumla D, Pereira JA, Dethoup T, Gales L, Freitas-Silva J, Costa PM, Lee M, Silva AMS, Sekeroglu N, Pinto MMM, Kijjoa A (2018) Chromone derivatives and other constituents from cultures of the marine sponge-associated fungus *Penicillium erubescens* KUFA0220 and their antibacterial activity. Mar Drugs 16(8):289

Kurata A, Sugiura M, Kokoda K, Tsujimoto H, Numata T, Kato C, Nakasone K, Kishimoto N (2017) Taxonomy of actinomycetes in the deep-sea calyptogena communities and characterization of the antibacterial compound produced by *Actinomadura* sp. DS-MS-114. Biotechnol Biotechnol Equip 31(5):1000−1006

Lai C, Chen J, Liu J, Tian D, Lan D, Liu T, Wu B, Bi H, Tang J (2022) New polyketides from a hydrothermal vent sediment fungus *Trichoderma* sp. JWM29-10-1 and their antimicrobial effects. Mar Drugs 20(11):720

Lai C, Tian D, Zheng M, Li B, Jia J, Wei J, Wu B, Bi H, Tang J (2023) Novel citrinin derivatives from fungus *Penicillium* sp. TW131-64 and their antimicrobial activities. Appl Microbiol Biotechnol 107(21):6607−6619

Lee DS, Eom SH, Jeong SY, Shin HJ, Je JY, Lee EW, Chung YH, Kim YM, Kang CK, Lee MS (2013) Anti-methicillin-resistant *Staphylococcus aureus* (MRSA) substance from the marine bacterium *Pseudomonas* sp. UJ-6. Environ Toxicol Pharmacol 35(2):171−177

Lee J, Choi JH, Lee J, Cho E, Lee YJ, Lee H-S, Oh KB (2024) Halenaquinol blocks staphylococcal protein A anchoring on cell wall surface by iinhibiting sortase A in *Staphylococcus aureus*. Mar Drugs 22(6):266

Lee J, Hwang JY, Oh D, Oh DC, Park HG, Shin J, Oh KB (2024) Tunicamycins from marine-derived *Streptomyces bacillaris* Inhibit MurNAc-pentapeptide translocase in *Staphylococcus aureus*. Mar Drugs 22(7):293

Lee J, Lee J, Kim GJ, Yang I, Wang W, Nam J-W, Choi H, Nam SJ, Kang H (2019) Mycousfurans A and B, antibacterial usnic acid congeners from the fungus *Mycosphaerella* sp., isolated from a marine sediment. Mar Drugs 17(7):422

Lei H, Lin X, Han L, Ma J, Dong K, Wang X, Zhong J, Mu Y, Liu Y, Huang X (2017) Polyketide derivatives from a marine-sponge-associated fungus *Pestalotiopsis heterocornis*. Phytochemistry (Elsevier) 142:51−59

Lei H, Lin X, Han L, Ma J, Ma Q, Zhong J, Liu Y, Sun T, Wang J, Huang X (2017) New metabolites and bioactive chlorinated benzophenone derivatives produced by a marine-derived fungus *Pestalotiopsis heterocornis*. Mar Drugs 15(3):69

Leutou AS, Yun K, Choi HD, Kang JS, Son BW (2012) New production of 5-bromotoluhydroquinone and 4-O-methyltoluhydroquinone from the marine-derived fungus *Dothideomycete* sp. J Microbiol Biotechnol 22(1):80−83

Lever J, Kreuder F, Henry J, Hung A, Allard P-M, Brkljaca R, Rix C, Taki AC, Gasser RB, Kaslin J, Wlodkowic D, Wolfender J-L, Urban S (2022) Targeted isolation of antibiotic brominated alkaloids from the marine sponge *Pseudoceratina durissima* using virtual screening and molecular networking. Mar Drugs 20(9):554

Li HL, Li XM, Li X, Yang SQ, Wang BG (2019) Structure, absolute configuration and biological evaluation of polyoxygenated meroterpenoids from the marine algal-derived *Aspergillus terreus* EN-539. Phytochem Lett 32:138−142

Li H, Liu H, Zhang W, Liu H, Liu Z, Li S, Ye W, Zhu M. (2020). CN110862371.

Li JX, Xu QH, Shang RY, Liu Q, Luo XC, Lin HW, Jiao WH (2023) Aspergetherins A-D, new chlorinated biphenyls with anti-MRSA activity from the marine sponge symbiotic fungus *Aspergillus terreus* 164018. Chem Biodiversity 20(4):e202300010

Li JL, Jiang X, Liu X, He C, Di Y, Lu S, Huang H, Lin B, Wang D, Fan B (2019) Antibacterial anthraquinone dimers from marine derived fungus *Aspergillus* sp. Fitoterapia 133:1−4

Li S, Mou Q, Xu X, Qi S, Leung PHM (2018) Synergistic antibacterial activity between penicillenols and antibiotics against methicillin-resistant *Staphylococcus aureus*. R Soc Open Sci 5(5):172466

Li S, Tian X, Niu S, Zhang W, Chen Y, Zhang H, Yang X, Zhang W, Li W, Zhang S, Ju J, Zhang C (2011) Pseudonocardians A-C, new diazaanthraquinone derivatives from a deap-sea actinomycete *Pseudonocardia* sp. SCSIO 01299. Mar Drugs 9:1428−1439

Li S, Wei M, Chen G, Lin Y (2012) Two new dihydroisocoumarins from the endophytic fungus *Aspergillus* sp. collected from the South China Sea. Chem Nat Compd 48(3):371−373

Li X, Choi HD, Kang JS, Lee C-O, Son BW (2003) New polyoxygenated farnesylcyclohexenones, deacetoxyyanuthone A and its hydro derivative from the marine-derived fungus *Penicillium* sp. J Nat Prod 66(11):1499−1500

Li X, Kim SK, Nam KW, Kang JS, Choi HD, Son BW (2006) A new antibacterial dioxopiperazine alkaloid related to gliotoxin from a marine isolate of the fungus *Pseudallescheria*. J Antibiot 59(4):248−250

Li X, Li XM, Zhang P, Wang BG (2015) A new phenolic enamide and a new meroterpenoid from marine alga-derived endophytic fungus *Penicillium oxalicum* EN-290. J Asian Nat Prod Res 17(12):1204−1212

Li YX, Jiao WH, Zhou XC, Zhang J, Ye BP (2013) Secondary metabolites of *Aspergillus aculeatus* CGD12 isolated from mangrove and their antibacterial activities. Zhongguo Shiyan Fangjixue Zazhi 19(19):71−76

Li Y, Li X, Lee U, Kang JS, Choi HD, Son BW (2006) A new radical scavenging anthracene glycoside, asperflavin ribofuranoside, and polyketides from a marine isolate of the fungus *Microsporum*. Chem Pharm Bull 54(6):882−883

Li Y, Li X, Son BW (2005) Antibacterial and radical scavenging epoxycyclohexenones and aromatic polyols from a marine isolate of the fungus *Aspergillus*. Nat Prod Sci 11(3):136-138

Li Z, Peng C, Shen Y, Miao X, Zhang H, Lin H (2008) L,L-Diketopiperazines from *Alcaligenes faecalis* A72 associated with South China Sea sponge *Stelletta tenuis*. Biochem Syst Ecol 36(3):230−234

Lian XY, Zhang Z (2013) Indanomycin-related antibiotics from marine streptomyces antibioticus PTZ0016. Nat Prod Res 27(23):2161−2167

Liang XR, Miao FP, Song YP, Liu XH, Ji NY (2016) Citrinovirin with a new norditerpene skeleton from the marine algicolous fungus *Trichoderma citrinoviride*. Bioorg Med Chem Lett 26(20):5029−5031

Liang Y, Xie X, Chen L, Yan S, Ye X, Anjum K, Huang H, Lian X, Zhang Z (2016) Bioactive polycyclic quinones from marine *Streptomyces* sp. 182SMLY. Mar Drugs 14(1):10

Lim HJ, An JS, Bae ES, Cho E, Hwang S, Nam SJ, Oh KB, Lee SK, Oh D-C (2022) Ligiamycins A and B, decalin-amino-maleimides from the co-culture of *Streptomyces* sp. and *Achromobacter* sp. isolated from the marine wharf roach, *Ligia exotica*. Mar Drugs 20(2):83

Lin C, Huang R, Liu J, Li H, Zhu L, Huang X, Ding B, Liu L, Huang H, Tao Y (2023) Antibacterial polyketides isolated from the marine-derived fungus *Fusarium solani* 8388. J Fungi 9(9):875

Lin S, Li J, Chen W, He J, Shi Y, Jin H, Ding L (2023) A new antibacterial dihydroisocoumarin from the marine sponge-associated fungus *Aspergillus* sp. Chem Nat Compd 59(2):246−248

Linh KTP, Phong NV, Binh PT, Thao NP, Thung DC, Nam NH, Van Thanh N (2022) Structural determination of new metabolites from the sea snail *Turbo chrysostomus* by NMR and DFT calculation. J Mol Struct 1270:133881

Liu HB, Lauro G, O'Connor R, Lohith K, Bifulco G, Bewley C. (2017). Poecillasirin A: A new tri-indole alkaloid from a deep water *Poecillastra* sp. Abstracts 254th ACS National Meeting & Exposition ORGN-400

Liu HB, Lauro G, O'Connor RD, Lohith K, Kelly M, Colin P, Bifulco G, Bewley CA (2017) Tulongicin, an antibacterial tri-indole alkaloid from a deep-water *Topsentia* sp. sponge. J Nat Prod 80(9):2556−2560

Liu LL, Xu Y, Han Z, Li YX, Lu L, Lai PY, Zhong JL, Guo XR, Zhang XX, Qian PY (2012) Four new antibacterial xanthones from the marine-derived actinomycetes *Streptomyces caelestis*. Mar Drugs 10:2571−2583

Liu P, Wang P, Xu J (2017) Complete assignment of NMR data of alterporriol I. Chem Nat Compd 53(4):653−657

Liu XH, Miao FP, Li XD, Yin XL, Ji NY (2012) A new sesquiterpene from an endophytic *Aspergillus versicolor* strain. Nat Prod Commun 7(7):819−820

Liu XY, Xu WF, Hai Y, Shao CL, Wei MY (2020) Research on secondary metabolites and their antimicrobial activities of the marine fungus *Penicillium chrysogenum*. Zhongguo Haiyang Yaowu 39(3):30−34

Liu YJ, Zhang JL, Li C, Mu XG, Liu XL, Wang L, Zhao YC, Zhang P, Li XD, Zhang XX (2019) Antimicrobial secondary metabolites from the seawater-derived fungus *Aspergillus sydowii* SW9. Molecules 24(24):4596

Liu Y, Ding L, He J, Zhang Z, Deng Y, He S, Yan X (2021) A new antibacterial chromone from a marine sponge-associated fungus *Aspergillus* sp. LS57. Fitoterapia 154:105004

Liu Y, Li XM, Meng LH, Jiang WL, Xu GM, Huang CG, Wang BG (2015) Bisthiodiketopiperazines and acorane sesquiterpenes produced by the marine-derived fungus *Penicillium adametzioides* AS-53 on different culture media. J Nat Prod 78(6):1294−1299

Liu Z, Dong S, Xu J, Zeng M, Song H, Zhao Y (2007) Production of cysteine-rich antimicrobial peptide by digestion of oyster (Crassostrea gigas) with alcalase and bromelin. Food Control 19(3):231−235

Lu X, Xu Q, Liu X, Jiao B, Ni K. (2007). CN101033219.

Lu XL, Xu QZ, Shen YH, Liu XY, Jiao BH, Zhang WD, Ni KY (2008) Macrolactin S, a novel macrolactin antibiotic from marine *Bacillus* sp. Nat Prod Res 22(4):342−347

Luo M, Tang L, Dong Y, Huang H, Deng Z, Sun Y (2021) Antibacterial natural products lobophorin L and M from the marine-derived *Streptomyces* sp. 4506. Nat Prod Res 35(24):5581−5587

Lv D, Xia J, Guan X, Lai Q, Zhang B, Lin J, Shao Z, Luo S, Zhangsun D, Qin J-J, Wang W (2022) Indole diketopiperazine alkaloids isolated from the marine-derived fungus *Aspergillus chevalieri* MCCC M23426. Front Microbiol 13:950857

Lv H, Wang K, Xue Y, Chen J, Su H, Zhang J, Wu Y, Jia J, Bi H, Wang H, Hong K, Li X (2021) Three new metabolites from the marine-derived fungus *Aspergillus* sp. WHUF03110. Nat Prod

Commun 16(10):1934578X211055009

Ma M, Ge H, Yi W, Wu B, Zhang Z (2020) Bioactive drimane sesquiterpenoids and isocoumarins from the marine-derived fungus *Penicillium minioluteum* ZZ1657. Tetrahedron Lett 61(7):151504

Ma M, Yi W, Qin L, Lian XY, Zhang Z (2022) Talaromydien a and talaroisocoumarin A, new metabolites from the marine-sourced fungus *Talaromyces* sp. ZZ1616. Nat Prod Res 36(1):460−465

Maarisit W, Ueda K (2013) Antimicrobial metabolites from a marine-derived fungus. Indones J Pharm 24(3):163−169

Machado FP, Kumla D, Pereira JA, Sousa E, Dethoup T, Freitas-Silva J, Costa PM, Mistry S, Silva AMS, Kijjoa A (2021) Prenylated phenylbutyrolactones from cultures of a marine sponge-associated fungus *Aspergillus flavipes* KUFA1152. Phytochemistry (Elsevier) 185:112709

Machado FP, Rodrigues IC, Gales L, Pereira JA, Costa PM, Dethoup T, Mistry S, Silva AMS, Vasconcelos V, Kijjoa A (2022) New alkylpyridinium anthraquinone, isocoumarin, c-Glucosyl resorcinol derivative and prenylated pyranoxanthones from the culture of a marine sponge-associated fungus, *Aspergillus stellatus* KUFA 2017. Mar Drugs 20(11):672

Machado FP, Rodrigues IC, Georgopolou A, Gales L, Pereira JA, Costa PM, Mistry S, Hafez Ghoran S, Silva AMS, Dethoup T, Sousa E, Kijjoa A (2023) New hybrid phenalenone dimer, highly conjugated dihydroxylated C28 steroid and azaphilone from the culture extract of a marine sponge-associated fungus, *Talaromyces pinophilus* KUFA 1767. Mar Drugs 21(3):194

Mal'tsev II, Kuznetsova TA, Mikhailov VV, Elyakov GB (1991) Novel antibiotic from a marine streptomycete. Khim Prir Soedin(2):298

McArthur KA, Mitchell SS, Tsueng G, Rheingold A, White DJ, Grodberg J, Lam KS, Potts BCM (2008) Lynamicins A-E, chlorinated bisindole pyrrole antibiotics from a novel marine actinomycete. J Nat Prod 71(10):1732−1737

Medeiros MA, Lourenco A, Tavares MR, Curto MJM, Feio SS, Roseiro JC (2006) (-)-Agelasidine A from *Agelas clathrodes*. Z. Naturforsch., C: J Biosci 61(7):472−476

Mei WL, Zheng B, Zhao YX, Zhong HM, Chen X-LW, Zeng Y-B, Dong W-H, Huang J-L, Proksch P, Dai H-F (2012) Meroterpenes from endophytic fungus A1 of mangrove plant *Scyphiphora hydrophyllacea*. Mar Drugs 10:1993−2001

Mei XT, Xu D, Xu SB (2003) Bacteriostatic and anti-influenza virus effects of marine active material C03. Zhongshan Daxue Xuebao, Ziran Kexueban 42(5):67−69

Miao FP, Li XD, Liu XH, Cichewicz RH, Ji NY (2012) Secondary metabolites from an algicolous *Aspergillus versicolor* strain. Mar Drugs 10:131−139

Minh LTH, Anh NM, Huyen VTT, Quyen VT, Dao PT, Huong DTM, Cuong PV, Tuan CD, Kiem PV, Tai BH (2023) A pair of undescribed alkaloid enantiomers from marine sponge-derived fungus *Hamigera avellanea* and their antimicrobial and cytotoxic activities. Chem Biodiversity 20(11):e202301425

Moe MK, Haug T, Sydnes MO, Sperstad SV, Li C, Vaagsfjord LC, de la Vega E, Stensvaag K (2018) Paralithocins, antimicrobial peptides with unusual disulfide connectivity from the red king crab, *Paralithodes camtschaticus*. J Nat Prod 81(1):140−150

Mohamed GA, Ibrahim SRM, Badr JM, Youssef DTA (2014) Didemnaketals D and E, bioactive terpenoids from a Red Sea ascidian *Didemnum* species. Tetrahedron 70(1):35−40

Mokhlesi A, Stuhldreier F, Wex KW, Berscheid A, Hartmann R, Rehberg N, Sureechatchaiyan P, Chaidir C, Kassack MU, Kalscheuer R, Broetz-Oesterhelt H, Wesselborg S, Stork B, Daletos G, Proksch P (2017) Cyclic cystine-bridged peptides from the marine sponge *Clathria basilana* induce

apoptosis in tumor cells and depolarize the bacterial cytoplasmic membrane. J Nat Prod 80(11):2941−2952

Morehouse NJ, Graham KM, Cox SL, Johnson JA, Gray CA (2024) Isolation of an antimicrobial racemic phenalenone derivative from a marine-derived *Penicillium* sp. fungus. Can J Chem 102(3):86−90

Mou P, Zhang Q, Peng J, Jiang X, Zhang L, Zhou Z, Zhang C, Zhu Y (2021) Antibacterial phenylspirodrimanes from the marine-derived fungus *Stachybotrys* sp. SCSIO 40434. Fitoterapia 152:104937

Murshid SSA, Badr JM, Youssef DTA (2016) Penicillosides A and B: new cerebrosides from the marine-derived fungus *Penicillium* species. Rev Bras Farmacogn 26(1):29−33

Musa A, Abdelgawad MA, Shaker ME, El-Ghorab AH, Parambi DGT, Hamed AA, Sayed AM, Hassan HM, Aboseada MA (2022) Screening and molecular docking of bioactive metabolites of the Red Sea sponge *Callyspongia siphonella* as potential antimicrobial agents. Antibiotics (Basel, Switz.) 11(12):1682

Nagai K, Kamigiri K, Arao N, Suzumura K-I, Kawano Y, Yamaoka M, Zhang H, Watanabe M, Suzuki K (2003) YM-266183 and YM-266184, novel thiopeptide antibiotics produced by *Bacillus cereus* isolated from a marine sponge. *I. Taxonomy*, fermentation, isolation, physico-chemical properties and biological properties. J Antibiot 56(2):123−128

Nagao T, Adachi K, Araki M, Nishijima M, Chen CL, Sano H. (1997). JP09301970

Namikoshi M, Suzuki S, Meguro S, Nagai H, Koike Y, Kitazawa A, Kobayashi H, Oda T, Yamada J (2004) Manoalide derivatives from a marine sponge *Luffariella* sp. collected in Palau. Fish Sci 70(1):152−158

Needham J, Kelly MT, Ishige M, Andersen RJ (1994) Andrimid and moiramides A-C, metabolites produced in culture by a marine isolate of the bacterium *Pseudomonas fluorescens*: structure elucidation and biosynthesis. J Org Chem 59(8):2058

Nenkep V, Yun K, Son BW (2016) Oxysporizoline, an antibacterial polycyclic quinazoline alkaloid from the marine-mudflat-derived fungus *Fusarium oxysporum*. J Antibiot 69(9):709−711

Nenkep V, Yun K, Zhang D, Choi HD, Kang JS, Son BW (2010) Induced production of bromomethylchlamydosporols A and B from the marine-derived fungus *Fusarium tricinctum*. J Nat Prod 73(12):2061−2063

Newaz AW, Yong K, Lian XY, Zhang Z (2022) Streptoindoles A-D, novel antimicrobial indole alkaloids from the marine-associated actinomycete *Streptomyces* sp. ZZ1118. Tetrahedron 104:132598

Newaz AW, Yong K, Yi W, Wu B, Zhang Z (2023) Antimicrobial metabolites from the Indonesian mangrove sediment-derived fungus *Penicillium chrysogenum* sp. ZZ1151. Nat Prod Res 37(10):1702−1708

Nguyen HM, Ito T, Kurimoto SI, Ogawa M, Win NN, Hung VQ, Nguyen HT, Kubota T, Kobayashi JI, Morita H (2017) New merosesquiterpenes from a Vietnamese marine sponge of *Spongia* sp. and their biological activities. Bioorg Med Chem Lett 27(14):3043−3047

Nguyen HM, Ito T, Win NN, Kodama T, Hung VQ, Nguyen HT, Morita H (2016) New antibacterial sesquiterpene aminoquinones from a Vietnamese marine sponge of *Spongia* sp. Phytochem Lett 17:288−292

Nguyen HP, Zhang D, Lee U, Kang JS, Choi HD, Son BW (2007) Dehydroxychlorofusarielin B, an antibacterial polyoxygenated decalin derivative from the marine-derived fungus *Aspergillus* sp. J Nat Prod 70(7):1188−1190

Nguyen THA, Do TQ, Nguyen TL, Le Thi HM, Nguyen MA, Murphy BT, Dam TX, Huong DTM, Cuong PV (2023) New sesterterpenoid from the marine fungus *Penicillium oxalicum* M893. Nat Prod Commun 18(8):1934578X231191636

Ningsih BNS, Rukachaisirikul V, Pansrinun S, Phongpaichit S, Preedanon S, Sakayaroj J (2022) New aromatic polyketides from the marine-derived fungus *Pseudopithomyces maydicus* PSU-AMF350 and their antimicrobial activity. Nat Prod Res 36(19):4982−4989

Niu S, Huang S, Wang J, He J, Chen M, Zhang G, Hong B (2024) Malfilanol C, a new sesquiterpenoid isolated from the deep-sea-derived *Aspergillus puniceus* A2 fungus. Nat Prod Res 38(8):1362−1368

Niu S, Li S, Chen Y, Tian X, Zhang H, Zhang G, Zhang W, Yang X, Zhang S, Ju J, Zhang C (2011) Lobophorins E and F, new spirotetronate antibiotics from a South China Sea-derived *Streptomyces* sp. SCSIO 01127. J Antibiot 64(11):711−716

Niu S, Liu D, Hu X, Proksch P, Shao Z, Lin W (2014) Spiromastixones A-O, antibacterial chlorodepsidones from a deep sea-derived *Spiromastix* sp. fungus. J Nat Prod 77(4):1021−1030

Niu S, Liu D, Proksch P, Shao Z, Lin W (2015) New polyphenols from a deep sea *Spiromastix* sp. fungus, and their antibacterial activities. Mar Drugs 13(4):2526−2540

Niu S, Liu D, Shao Z, Huang J, Fan A, Lin W (2021) Chlorinated metabolites with antibacterial activities from a deep-sea-derived *Spiromastix fungus*. RSC Adv 11(47):29661−29667

Oh KB, Mar W, Kim S, Kim JY, Lee TH, Kim JG, Shin D, Sim CJ, Shin J (2006) Antimicrobial activity and cytotoxicity of bis(indole) alkaloids from the sponge *Spongosorites* sp. Biol Pharm Bull 29(3):570−573

Oh KB, Mar W, Kim S, Kim JY, Oh MN, Kim JG, Shin D, Sim CJ, Shin J (2005) Bis(indole) alkaloids as sortase A inhibitors from the sponge *Spongosorites* sp. Bioorg Med Chem Lett 15(22):4927−4931

Okimura K, Matsubara K, Suzuki R, Ito H, Sato A, Sugimoto K (2021) Examination of the antimicrobial peptide myticalin A6 active site. Biol Pharm Bull 44(4):515−521

Olson E, Trew SJ, Wrigley SK, Pairet L, Hayes MA, Martin S, Kau DA. (1998). WO9825931

Pan C, Shi Y, Auckloo BN, ul Hassan SS, Akhter N, Wang K, Ye Y, Chen C-TA, Tao X, Wu B (2017) Isolation and antibiotic screening of fungi from a hydrothermal vent site and characterization of secondary metabolites from a *Penicillium* Isolate. Mar Biotechnol 19(5):469−479

Pan D, Lin S, Huang X, Lv D, Lai Q, Xia J, Shao Z, Wang W (2023) New polyketides and sesquiterpenoids from the deep-sea sulphide-derived fungus *Phoma* sp. 3A00413. Fitoterapia 168:105546

Pang X, Cai G, Lin X, Salendra L, Zhou X, Yang B, Wang J, Wang J, Xu S, Liu Y (2019) New alkaloids and polyketides from the marine sponge-derived fungus *Penicillium* sp. SCSIO41015. Mar Drugs 17(7):398

Pang X, Chen W, Wang X, Zhou X, Yang B, Tian X, Wang J, Xu S, Liu Y (2021) New tetramic acid derivatives from the deep-sea-derived fungus *Penicillium* sp. SCSIO06868 with SARS-CoV-2 Mpro inhibitory activity evaluation. Front Microbiol 12:730807

Pang X, Lin X, Wang P, Zhou X, Yang B, Wang J, Liu Y (2018) Perylenequione derivatives with anticancer activities Isolated from the marine sponge-derived fungus, *Alternaria* sp. SCSIO41014. Mar Drugs 16(8):280

Pang X, Zhou X, Lin X, Yang B, Tian X, Wang J, Xu S, Liu Y (2021) Structurally various sorbicillinoids from the deep-sea sediment derived fungus *Penicillium* sp. SCSIO06871. Bioorg Chem

107:104600

Pech-Puch D, Forero AM, Fuentes-Monteverde JC, Lasarte-Monterrubio C, Martinez-Guitian M, Gonzalez-Salas C, Guillen-Hernandez S, Villegas-Hernandez H, Beceiro A, Griesinger C, Rodriguez J, Jimenez C (2022) Antimicrobial diterpene alkaloids from an agelas citrina sponge collected in the Yucatan Peninsula. Mar Drugs 20(5):298

Peela S, Girijashankar V, Ponamgi SPD, Mandali N, Nagaraj GP (2019) Novel tetracycline SBR-22 is a functional moiety deviation and bioactive against multidrug resistant strains. Process Biochem (Oxford, U. K.) 85:213−218

Pettit GR, Butler MS, Bass CG, Doubek DL, Williams MD, Schmidt JM, Pettit RK, Hooper JNA, Tackett LP, Filliatrault MJ (1995) Antineoplastic agents, 326. The stereochemistry of bastadins 8, 10, and 12 from the Bismarck Archipelago marine sponge *Lanthella basta*. J Nat Prod 58(5):680

Pettit GR, Cichacz ZA, Tan R, Hoard MS, Melody N, Pettit RK (1998) Antineoplastic agents. 386. isolation of sesterstatins 1-3 from the marine sponge *Hyrtios erecta*. J Nat Prod 61(1):13-16

Pham VC, Doan TMH, Do TQ, Le THM, Trinh TTV, Vo TQ, Vo TH. (2024). VN10042155

Phi Thi D, Doan Thi Mai H, Cao DD, Vu Thi Q, Nguyen MA, Le Thi HM, Tran DT, Chau VM, Pham VC (2020) Novel 1,3-benzodioxole from marine-derived actinomycete in East Vietnam Sea. Nat Prod Commun 15(5):1934578X20920042

Popov AM, Stekhova SI, Utkina NK, Rebachuk NM (1999) Antimicrobial and cytotoxic activity of sesquiterpenequinones and brominated diphenyl esters isolated from marine sponges. Pharm Chem J 33(2):71−73

Poumale HMP, Ngadjui BT, Helmke E, Laatsch H (2006) New anthraquinones from a marine *Streptomyces* sp. - isolation, structure determination and biological activities. Z Naturforsch, B: Chem Sci 61(11):1450−1454

Prompanya C, Dethoup T, Bessa LJ, Pinto MMM, Gales L, Costa PM, Silva AMS, Kijjoa A (2014) New isocoumarin derivatives and meroterpenoids from the marine sponge-associated fungus *Aspergillus similanensis* sp. nov. KUFA 0013. Mar Drugs 12(10):5160−5173

Qader M, Mweetwa LL, Rämä T, Thissera B, Milne BF, Abdelmohsen UR, Orfali R, Tawfike A, Esheli M, Oluwabusola ET, Jaysainghe L, Jaspars M, Rateb ME (2023) Discovery and structural assignment of (S)-sydosine from amphipod-derived *Aspergillus sydowii* MBC15-11F through HRMS, advanced Mosher, and molecular modelling analyses. J Appl Microbiol 134(8): lxad158

Qi J, Jiang L, Zhao P, Chen H, Jia X, Zhao L, Dai H, Hu J, Liu C, Shim SH, Xia X, Zhang L (2020) Chaetoglobosins and azaphilones from *Chaetomium globosum* associated with *Apostichopus japonicus*. Appl Microbiol Biotechnol 104(4):1545−1553

Quévrain E, Domart-Coulon I, Pernice M, Bourguet-Kondracki M-L (2009) Novel natural parabens produced by a *Microbulbifer* bacterium in its calcareous sponge host Leuconia nivea. Environ Microbiol 11(6):1527−1539

Quynh DT, Van TTT, Linh NT, Minh LTH, Quyen VT, Murphy BT, Huong DTM, Van Cuong P (2023) A new alkaloid from marine-derived actinomycete *Actinoalloteichus cyanogriseus* G631. Rec Nat Prod 17(6):1024−1030

Ragini K, Piggott AM, Karuso P (2019) Bisindole alkaloids from a new zealand deep-seamarine sponge *Lamellomorpha strongylata*. Mar Drugs 17(12):683

Ramsey DM, Amirul Islam M, Turnbull L, Davis RA, Whitchurch CB, McAlpine SR (2013) Psammaplysin F: A unique inhibitor of bacterial chromosomal partitioning. Bioorg Med Chem Lett 23(17):4862−4866

Rateb ME, Houssen WE, Legrave NM, Clements C, Jaspars M, Ebel R (2010) Dibenzofurans from the marine sponge-derived ascomycete Super1F1-09. Bot Mar 53(6):499−506

Ren L, Leng X, Li T, Liu J, Wang W, Yan X, Yan X, He S (2023) Four new cembranoids from the South China Sea soft coral *Sarcophyton trocheliophorum*. Nat Prod Res 37(1):39−46

Ren Z, Yang L, Ma Q, Xie Q, Dai H, Sun K, Zhao Y (2022) Meroterpenoids and steroids from the marine-derived fungus *Trametes* sp. ZYX-Z-16. Molecules 27(24):8782

Riedlinger J, Reicke A, Zaehner H, Krismer B, Bull AT, Maldonado LA, Ward AC, Goodfellow M, Bister B, Bischoff D, Suessmuth RD, Fiedler HP (2004) Biosynthetic capacities of actinomycetes: Abyssomicins, inhibitors of the para-aminobenzoic acid pathway produced by the marine *Verrucosispora* strain AB-18-032. J Antibiot 57(4):271−279

Rodrigues D, Alves C, Horta A, Pinteus S, Silva J, Culioli G, Thomas OP, Pedrosa R (2015) Antitumor and antimicrobial potential of bromoditerpenes isolated from the red alga, *Sphaerococcus coronopifolius*. Mar Drugs 13(2):713

Roue M, Domart-Coulon I, Ereskovsky A, Djediat C, Perez T, Bourguet-Kondracki M-L (2010) Cellular localization of clathridimine, an antimicrobial 2-aminoimidazole alkaloid produced by the Mediterranean Calcareous sponge *Clathrina clathrus*. J Nat Prod 73(7):1277−1282

Ryu MJ, Hillman PF, Lee J, Hwang S, Lee EY, Cha SS, Yang I, Oh DC, Nam SJ, Fenical W (2021) Antibacterial meroterpenoids, merochlorins G-J from the marine macterium *Streptomyces* sp. Mar Drugs 19(11):618

Ryu MJ, Hwang S, Kim S, Yang I, Oh DC, Nam SJ, Fenical W (2019) Meroindenon and merochlorins E and F, antibacterial meroterpenoids from a marine-derived sediment bacterium of the genus *Streptomyces*. Org Lett 21(15):5779−5783

Sabdaningsih A, Liu Y, Mettal U, Heep J, Riyanti, Wang L, Cristianawati O, Nuryadi H, Sibero MT, Marner M, Radjasa OK, Sabdono A, Trianto A, Schaeberle TF (2020) A new citrinin derivative from the Indonesian marine sponge-associated fungus *Penicillium citrinum*. Mar Drugs 18(4):227

Salman S, Shah FH, Shah M, Kim SJ (2024) Molecular docking, acute toxicity and antibacterial study of debilon and phorbasterone-B extracted from *Rhodophyta*. Lett. Drug Des. Discovery 21(10):1858−1863

Satitpatipan V, Suwanborirux K (2004) New nitrogenous germacranes from a Thai marine sponge, *Axinyssa n.* sp. J Nat Prod 67(3):503−505

Sayed AM, Alhadrami HA, El-Hawary SS, Mohammed R, Hassan HM, Rateb ME, Abdelmohsen UR, Bakeer W (2020) Discovery of two brominated oxindole alkaloids as staphylococcal DNA gyrase and pyruvate kinase inhibitors via inverse virtual screening. Microorganisms 8(2):293

Schneider Y, Jenssen M, Isaksson J, Hansen KS, Andersen JH, Hansen EH (2020) Bioactivity of serratiochelin a, a siderophore isolated from a co-culture of *Serratia* sp. and *Shewanella* sp. Microorganisms 8(7):1042

Selim RM, Shaaban M, Hamdy AA, Abou Zeid AA, Ata A (2019) Viscosine: A new microbial flavonoid from marine-derived, *Streptomyces* sp. RMS518F. Vietnam J Chem 57(3):288-295

Shaala LA, Alzughaibi T, Genta-Jouve G, Youssef DTA (2021) Fusaripyridines A and B; highly oxygenated antimicrobial alkaloid dimers featuring an unprecedented 1,4-bis(2-hydroxy-1,2-dihydropyridin-2-yl)butane-2,3-dione core from the marine fungus *Fusarium* sp. LY019. Mar Drugs 19(9):505

Shaala LA, Youssef DTA, Alzughaibi TA, Elhady SS (2020) Antimicrobial chlorinated 3-

phenylpropanoic acidderivatives from the red sea marine actinomycete *Streptomyces coelicolor* LY001. Mar Drugs 18(9):450

Shao CL, Wang CY, Deng DS, She ZG, Gu YC, Lin YC (2008) Crystal structure of a marine natural compound, anhydrofusarubin. Chin J Struct Chem 27(7):824−828

Shao CL, Hu G, Yang R, Xia XK, Wang C, She ZG, Lin YC (2008) Secondary metabolites of mangrove endophytic fungus B77 in south china sea. Zhongshan Daxue Xuebao, Ziran Kexueban 47(1):56−58

Shin HJ, Lee HS, Lee DS (2010) The synergistic antibacterial activity of 1-acetyl-*β*-carboline and *β*-lactams against methicillin-resistant *Staphylococcus aureus* (MRSA). J Microbiol Biotechnol 20(3):501−505

Shiozawa H, Shimada A, Takahashi S (1997) Thiomarinols D, E, F and G, new hybrid antimicrobial antibiotics produced by a marine bacterium; isolation, structure, and antimicrobial activity. J Antibiot 50(5):449−452

Shiyanga AN, Knott M, Kapewangolo P (2020) In vitro anti-HIV and antimicrobial activities of a halogenated monoterpene from a *Namibian Plocamium* species of marine algae. Curr Bioact Compd 16(3):363−367

Shridhar DMP, Mahajan GB, Kamat VP, Naik CG, Parab RR, Thakur NR, Mishra PD (2009) Antibacterial activity of 2-(2',4'-Dibromophenoxy)-4,6-dibromophenol from *Dysidea granulosa*. Mar Drugs 7(3):464−471

Singh S, Prasad P, Subramani R, Aalbersberg W (2014) Production and purification of bioactive substance against multi-drug resistant human pathogens from marine-sponge-derived *Salinispora* sp. Asian Pac J Trop Biomed 4(10):825−831

Sletta H, Klinkenberg G, Floegstad DK, Ellingsen TE, Nordborg A, Hakvaag S. (2018). NO341815

Smyrniotopoulos V, Vagias C, Rahman MM, Gibbons S, Roussis V (2010) Ioniols I and II, tetracyclic diterpenes with antibacterial activity, from *Sphaerococcus coronopifolius*. Chem Biodiversity 7(3):666−676

Socha AM, LaPlante KL, Rowley DC (2006) New bisanthraquinone antibiotics and semi-synthetic derivatives with potent activity against clinical *Staphylococcus aureus* and *Enterococcus faecium* isolates. Bioorg Med Chem 14(24):8446−8454

Socha AM, LaPlante KL, Russell DJ, Rowley DC (2009) Structure-activity studies of echinomycin antibiotics against drug-resistant and biofilm-forming *Staphylococcus aureus* and *Enterococcus faecalis*. Bioorg Med Chem Lett 19(5):1504−1507

Song F, Dong Y, Wei S, Zhang X, Zhang K, Xu X (2022) New antibacterial secondary metabolites from a marine-derived *Talaromyces* sp. strain BTBU20213036. Antibiotics (Basel, Switz.) 11(2):222

Song F, Hu J, Zhang X, Xu W, Yang J, Li S, Xu X (2022) Unique cyclized thiolopyrrolones from the marine-derived *Streptomyces* sp. BTBU20218885. Mar Drugs 20(3):214

Song F, Lin R, Yang N, Jia J, Wei S, Han J, Li J, Bi H, Xu X (2021) Antibacterial secondary metabolites from marine-derived fungus *Aspergillus* sp. IMCASMF180035. Antibiotics (Basel, Switz.) 10(4):377

Song F, Ren B, Chen C, Yu K, Liu X, Zhang Y, Yang N, He H, Liu X, Dai H, Zhang L (2014) Three new sterigmatocystin analogues from marine-derived fungus *Aspergillus versicolor* MF359. Appl Microbiol Biotechnol 98(8):3753−3758

Song T, Chen M, Chai W, Zhang Z, Lian XY (2018) New bioactive pyrrospirones C-I from a marine-derived fungus *Penicillium* sp. ZZ380. Tetrahedron 74(8):884−891

Song T, Tang M, Ge H, Chen M, Lian X, Zhang Z (2019) Novel bioactive penicipyrroether a and pyrrospirone J from the marine-derived *Penicillium* sp. ZZ380. Mar Drugs 17(5):292

Song Y, Huang H, Chen Y, Ding J, Zhang Y, Sun A, Zhang W, Ju J (2013) Cytotoxic and antibacterial marfuraquinocins from the deep South China Sea-derived *Streptomyces niveus* SCSIO 3406. J Nat Prod 76(12):2263−2268

Song Y, Li Q, Liu X, Chen Y, Zhang Y, Sun A, Zhang W, Zhang J, Ju J (2014) Cyclic hexapeptides from the deep South China Sea-derived *Streptomyces scopuliridis* SCSIO ZJ46 active against pathogenic Gram-positive bacteria. J Nat Prod 77(8):1937−1941

Song Y, Yang J, Yu J, Li J, Yuan J, Wong NK, Ju J (2020) Chlorinated bis-indole alkaloids from deep-sea derived *Streptomyces* sp. SCSIO 11791 with antibacterial and cytotoxic activities. J Antibiot 73(8):542−547

Sun C, Ha Y, Liu X, Wang N, Lian XY, Zhang Z (2024) Isolation and structure elucidation of new metabolites from the mariana-trench-associated fungus *Aspergillus* sp. SY2601. Molecules 29(2):459

Sun K, Li Y, Guo L, Wang Y, Liu P, Zhu W (2014) Indole diterpenoids and isocoumarin from the fungus, *Aspergillus flavus*, isolated from the prawn, *Penaeus vannamei*. Mar Drugs 12(7):3970−3981

Sun P, Maloney KN, Nam SJ, Haste NM, Raju R, Aalbersberg W, Jensen PR, Nizet V, Hensler ME, Fenical W (2011) Fijimycins A-C, three antibacterial etamycin-class depsipeptides from a marine-derived *Streptomyces* sp. Bioorg Med Chem 19(22):6557−6562

Sun RR, Miao FP, Zhang J, Wang G, Yin XL, Ji NY (2013) Three new xanthone derivatives from an algicolous isolate of *Aspergillus wentii*. Magn Reson Chem 51(1):65−68

Sun S, Canning CB, Bhargava K, Sun X, Zhu W, Zhou N, Zhang Y, Zhou K (2015) Polybrominated diphenyl ethers with potent and broad spectrum antimicrobial activity from the marine sponge *Dysidea*. Bioorg Med Chem Lett 25(10):2181−2183

Sunga MJ, Teisan S, Tsueng G, Macherla VR, Lam KS (2008) Seawater requirement for the production of lipoxazolidinones by marine *Actinomycete* strain NPS8920. J Ind Microbiol Biotechnol 35(7):761−765

Tabakmakher KM, Makarieva TN, Sabutski YE, Kokoulin MS, Menshov AS, Popov RS, Guzii AG, Shubina LK, Chingizova EA, Chingizov AR, Yurchenko EA, Fedorov SN, Grebnev BB, von Amsberg G, Dyshlovoy SA, Ivanchina NV, Dmitrenok PS (2024) Stonikacidin A, an antimicrobial 4-bromopyrrole alkaloid containing L-idonic acid core from the Northwestern Pacific marine sponge *Lissodendoryx papillosa*. Mar Drugs 22(9):396

Tai CJ, Chao CH, Ahmed AF, Yen CH, Hwang TL, Chang FR, Huang YM, Sheu JH (2023) New 3,4-seco-3,19-dinor- and spongian-based diterpenoid lactones from the marine sponge *Spongia* sp. Int J Mol Sci 24(2):1252

Tammam MA, Tsoureas N, Diakaki DI, Duarte CM, Roussis V, Ioannou E (2025) Thuwalamides A-E: Polychlorinated amides from the marine sponge *Lamellodysidea herbacea* collected from the Saudi Arabian Red Sea. Phytochemistry (Elsevier) 230:114315

Tan N, Pan JH, Peng GT, Mou CB, Tao YW, She ZG, Yang ZL, Zhou SN, Lin YC (2008) A copper coordination compound produced by a marine fungus *Fusarium* sp. ZZF51 with biosorption of Cu(II) ions. Chin J Chem 26(3):516−521

Tan S, Liu Y, Fu H, Xue Y, Feng F, Li J, Yan X, Wu S, He S, Ding L (2024) Quinosumycin, a novel anti-MRSA thioether-linked quinolinone-quinazolinone heterodimer from a marine actinomycete *Streptomyces diastaticus* NBU2966. J Oceanol Limnol 42(6):1991−997

Tanaka N, Suto S, Asai M, Kusama T, Takahashi-Nakaguchi A, Gonoi T, Fromont J, Kobayashi J-i (2015) Halichonadins M-Q, sesquiterpenes from an Okinawan marine sponge *Halichondria* sp. Heterocycles 90(1):173−185

Tang L, Xia J, Chen Z, Lin F, Shao Z, Wang W, Hong X (2024) Cytotoxic and antibacterial meroterpenoids isolated from the marine-derived fungus *Talaromyces* sp. M27416. Mar Drugs 22(4):186

Tao HM, Lin XP, He YY, Zhang L, Liu YH (2015) Antibacterial metabolites of marine fungus *Penicillium* sp. F00120 from the deep sea sediments in the South China Sea. Zhongguo Yaoxue Zazhi (Beijing, China) 50(9):755−757

Tareq FS, Kim JH, Lee MA, Lee H-S, Lee JS, Lee YJ, Shin HJ (2013) Antimicrobial gageomacrolactins characterized from the fermentation of the marine-derived bacterium *Bacillus subtilis* under optimum growth conditions. J Agric Food Chem 61(14):3428−3434

Tareq FS, Lee HS, Lee YJ, Lee JS, Shin HJ (2015) Ieodoglucomide C and Ieodoglycolipid, new glycolipids from a marine-derived bacterium *Bacillus licheniformis* 09IDYM23. Lipids 50(5):513−519

Tareq FS, Lee MA, Lee HS, Lee JS, Lee YJ, Shin HJ (2014) Gageostatins A-C, antimicrobial linear lipopeptides from a marine *Bacillus subtilis*. Mar Drugs 12(2):871

Tasiemski A, Schikorski D, Le Marrec-Croq F, Pontoire-Van Camp C, Boidin-Wichlacz C, Sautiere P-E (2007) Hedistin: A novel antimicrobial peptide containing bromotryptophan constitutively expressed in the NK cells-like of the marine annelid, *Nereis diversicolor*. Dev Comp Immunol 31(8):749−762

Teeyapant R, Woerdenbag HJ, Kreis P, Hacker J, Wray V, Witte L, Proksch P (1993) Antibiotic and cytotoxic activity of brominated compounds from the marine sponge *Verongia aerophoba*. Z Naturforsch, C: Biosci 48(11):939

Thamsiri P, Rukachaisirikul V, Phongpaichit S, Preedanon S, Sakayaroj J, Muanprasat C, Hadsadee S, Jungsuttiwong S (2024) Penicitrisochromans A-C, new isochromans from the marine-derived fungus *Penicillium citrinum* PSU-MF100. Nat Prod Res 20:1−7

Thi Hoang Anh N, Mai Anh N, Thi Thu Huyen V, Thi Dao P, Thi Mai Huong D, Van Cuong P, Thanh Xuan D, Huu Tai B, Thi Hong Minh L, Van Kiem P (2023) Antimicrobial activity of depsidones and macrocyclic peptides Isolated from marine sponge-derived fungus *Aspergillus nidulans* M256. Chem Biodiversity 20(12):e202301660

Thuy NTT, Minh Hien HT, Ha NC, Thom LT, Hong DD, Thinh NV, Dan NT, Hoi ND, Loan VT, Quang HD, Hang DTT, Kiem PV, Dat NT, Nhiem NX (2023) Chemical constituents from the marine microalgae *Thraustochytrium pachydermum*. Nat Prod Commun 18(3):1934578X231157145

Tian Y, Chen Z, Ma M, Jiang Y (2023) Nocardiopyrone C, a new antimicrobial pyran-2-one derivative from a marine-derived actinomycete strain *Nocardiopsis aegyptia* ZSN1. Rec Nat Prod 17(5):952−957

Tilvi S, Majik MS (2019) 2D NMR studies of bromotyrosine alkaloid, purpurealidin K from marine sponge *Psammaplysilla purpurea*. ChemistrySelect 4(21):6568−6571

Tilvi S, Rodrigues C, Naik CG, Parameswaran PS, Wahidhulla S (2004) New bromotyrosine alkaloids from the marine sponge *Psammaplysilla purpurea*. Tetrahedron 60(45):10207−10215

Torres YR, Berlinck RGS, Nascimento GGF, Fortier SC, Pessoa C, de Moraes MO (2002) Antibacterial activity against resistant bacteria and cytotoxicity of four alkaloid toxins isolated from the marine sponge *Arenosclera brasiliensis*. Toxicon 40(7):885−891

Tran HNK, To LH, Heo SJ, Kim EA, Kang N, Kim MJ, Tran LVH, Lee YJ (2025) Sesterterpenoids isolated from the marine sponge *Coscinoderma bakusi*. Phytochemistry (Elsevier) 230:114331

Trisuwan K, Rukachaisirikul V, Sukpondma Y, Preedanon S, Phongpaichit S, Rungjindamai N, Sakayaroj J (2008) Epoxydons and a pyrone from the marine-derived fungus *Nigrospora* sp. PSU-F5. J Nat Prod 71(8):1323−1326

Trisuwan K, Rukachaisirikul V, Sukpondma Y, Preedanon S, Phongpaichit S, Sakayaroj J (2009) Pyrone derivatives from the marine-derived fungus *Nigrospora* sp. PSU-F18. Phytochemistry (Elsevier) 70(4):554−557

Tsukamoto S, Yamashita Y, Ohta T (2005) New cytotoxic and antibacterial compounds isolated from the sea hare, *Aplysia kurodai*. Mar Drugs 3(2):22−28

Tsuruda S, Sato T, Aoyama H, Sirimangkalakitti N, Fujimura S, Arisawa M, Murai K (2024) Total synthesis and antibacterial activity of marine tris-indole alkaloid tulongicin A. J Nat Prod 87(6):1556−1562

Tu L, Shen S, Yan Z, Li X, Liu K, Xu J, Luo M (2024) Discovery of olimycin E from *Streptomyces* sp. 11695. Nat Prod Res 39(13):3908−3913

Uzair B, Ahmed N, Ahmad VU, Kousar F (2006) A new antibacterial compound produced by an indigenous marine bacteria - fermentation, isolation, and biological activity. Nat Prod Res 20(14):1326−1331

Uzair B, Ahmed N, Ahmad VU, Mohammad FV, Edwards DH (2008) The isolation, purification and biological activity of a novel antibacterial compound produced by *Pseudomonas stutzeri*. FEMS Microbiol Lett 279(2):243−250

Vairappan CS, Lee TK (2009) C-15 halogenated acetogenin with antibacterial activity against food pathogens. Malays. J Sci 28(3):263−268

Wang CY, Wang BG, Wiryowidagdo S, Wray V, van Soest R, Steube KG, Guan HS, Proksch P, Ebel R (2003) Melophlins C-O, thirteen novel tetramic acids from the marine sponge *Melophlus sarassinorum*. J Nat Prod 66(1):51−56

Wang FR, Zhang SL, Kong FD, Ma QY, Xie QY, Dai HF, Chen P, Zhao YX (2021) Secondary metabolites and their antibacterial activity from the marine fungus *Aspergillus clavatonanicus* HNMF114. Tianran Chanwu Yanjiu Yu Kaifa 33(6):76−81

Wang G, Dai S, Chen M, Wu H, Xie L, Luo X, Li X (2010) Two diketopiperazine cyclo(Pro-Phe) isomers from marine bacterium *Bacillus subtilis* sp. 13-2. Chem Nat Compd 46(4):583−585

Wang J, Nong XH, Amin M, Qi SH (2018) Hygrocin C from marine-derived Streptomyces sp. SCSGAA 0027 inhibits biofilm formation in *Bacillus amyloliquefaciens* SCSGAB0082 isolated from South China Sea gorgonian. Appl Microbiol Biotechnol 102(3):1417−1427

Wang L, Linares-Otoya V, Liu Y, Mettal U, Marner M, Armas-Mantilla L, Willbold S, Kurtan T, Linares-Otoya L, Schaeberle TF (2022) Discovery and biosynthesis of antimicrobial phenethylamine alkaloids from the marine flavobacterium *Tenacibaculum discolor* sv11. J Nat Prod 85(4):1039−1051

Wang L, Marner M, Mettal U, Liu Y, Schaeberle TF (2022) Seven new alkaloids isolated from marine flavobacterium *Tenacibaculum discolor* sv11. Mar Drugs 20(10):620

Wang ML, Lu CH, Xu QY, Song SY, Hu ZY, Zheng ZH (2013) Four new citrinin derivatives from a marine-derived *Penicillium* sp. fungal strain. Molecules 18:5723−5735

Wang Q, Zhang Y, Wang M, Tan Y, Hu X, He H, Xiao C, You X, Wang Y, Gan M (2017) Neo-actinomycins A and B, natural actinomycins bearing the 5H-oxazolo[4,5-b]phenoxazine

chromophore, from the marine-derived *Streptomyces* sp. IMB094. Sci Rep 7(1):1−8

Wang R, Guo ZK, Li XM, Chen FX, Zhan XF, Shen MH (2015) Spiculisporic acid analogues of the marine-derived fungus, *Aspergillus candidus* strain HDf2, and their antibacterial activity. Antonie Van Leeuwenhoek 108(1):215−219

Wang W, Chen R, Luo Z, Wang W, Chen J (2017) Two new benzoate derivatives and one new phenylacetate derivative from a marine-derived fungus *Engyodontium album*. Nat Prod Res 31(7):758−764

Wang W, Gao M, Luo Z, Liao Y, Zhang B, Ke W, Shao Z, Li F, Chen J (2019) Secondary metabolites isolated from the deep sea-derived fungus *Aspergillus sydowii* C1-S01-A7. Nat Prod Res 33(21):3077−3082

Wang W, Liao Y, Chen R, Hou Y, Ke W, Zhang B, Gao M, Shao Z, Chen J, Li F (2018) Chlorinated azaphilone pigments with antimicrobial and cytotoxic activities isolated from the deep sea derived fungus *Chaetomium* sp. NA-S01-R1. Mar Drugs 16(2):61

Wang W, Liao Y, Zhang B, Gao M, Ke W, Li F, Shao Z (2019) Citrinin monomer and dimer derivatives with antibacterial and cytotoxic activities isolated from the deep sea-derived fungus *Penicillium citrinum* NLG-S01-P1. Mar Drugs 17(1):46

Wang W, Wang J, Song F, Jia R, Wang L, Xu X, Yang N (2024) New secondary metabolites from marine-derived fungus *Talaromyces minnesotensis* BTBU20220184. Mar Drugs 22(6):237

Wang Z, Zeng Y, Zhao W, Dai H, Chang W, Lv F (2022) Structures and biological activities of brominated azaphilones produced by *Penicillium sclerotiorum* E23Y-1A. Phytochem Lett 52:138−142

Wen HM, Zhang YW, Feng FJ, Huang GB, Lv YH, Zhang ZY, Ding LJ (2024) Antibacterial oxygenated ergostane-type steroids produced by the marine sponge-derived fungus *Aspergillus* sp. J Asian Nat Prod Res 26(5):548−554

Wiese J, Abdelmohsen UR, Motiei A, Humeida UH, Imhoff JF (2018) Bacicyclin, a new antibacterial cyclic hexapeptide from *Bacillus* sp. strain BC028 isolated from *Mytilus edulis*. Bioorg Med Chem Lett 28(4):558−561

Wisespongpand P, Kuniyoshi M (2003) Bioactive phloroglucinols from the brown alga *Zonaria diesingiana*. J Appl Phycol 15(2-3):225−228

Wratten SJ, Faulkner DJ, Hirotsu K, Clardy J (1978) Diterpenoid isocyanides from the marine sponge *Hymeniacidon amphilecta*. Tetrahedron Lett(45):4345

Wratten SJ, Wolfe MS, Andersen RJ, Faulkner DJ (1977) Antibiotic metabolites from a marine pseudomonad. Antimicrob Agents Chemother 11(3):411

Wu B, Ohlendorf B, Oesker V, Wiese J, Malien S, Schmaljohann R, Imhoff JF (2015) Acetylcholinesterase inhibitors from a marine fungus *Talaromyces* sp. strain LF458. Mar Biotechnol 17(1):110−119

Wu B, Wiese J, Labes A, Kramer A, Schmaljohann R, Imhoff JF (2015) Lindgomycin, an unusual antibiotic polyketide from a marine fungus of the *Lindgomycetaceae*. Mar Drugs 13(8):4617−4632

Wu B, Wiese J, Wenzel-Storjohann A, Malien S, Schmaljohann R, Imhoff JF (2016) Engyodontochones, antibiotic polyketides from the marine fungus *Engyodontium album* strain LF069. Chem Eur J 22(22):7452−7462

Wu C, Tan Y, Gan M, Wang Y, Guan Y, Hu X, Zhou H, Shang X, You X, Yang Z, Xiao C (2013) Identification of elaiophylin derivatives from the marine-derived actinomycete *Streptomyces* sp. 7-145 using PCR-based screening. J Nat Prod 76(11):2153-2157

Wu JS, Meng QY, Zhang YH, Shi XH, Fu XM, Zhang P, Li X, Shao CL, Wang CY (2020) Annular oxygenation and rearrangement products of cryptotanshinone by biotransformation with marine-derived fungi *Cochliobolus lunatus* and *Aspergillus terreus*. Bioorg Chem 103:104192

Wu JS, Yao GS, Shi XH, Rehman SU, Xu Y, Fu XM, Zhang XL, Liu Y, Wang CY (2020) Epigenetic agents trigger the production of bioactive nucleoside derivatives and bisabolane sesquiterpenes from the marine-derived fungus *Aspergillus versicolor*. Front Microbiol 11:85

Wu J, Shui H, Zhang M, Zeng Y, Zheng M, Zhu KK, Wang SB, Bi H, Hong K, Cai YS (2023) Aculeaxanthones A-E, new xanthones from the marine-derived fungus *Aspergillus aculeatinus* WHUF0198. Front Microbiol 14:1138830

Wu J, Wang W, Yang Y, Shah M, Peng J, Zhou L, Zhang G, Che Q, Li J, Zhu T, Li D (2024) Phenylhydrazone alkaloids from the deep-sea cold seep derived fungus *Talaromyces amestolkiae* HDN21-0307. J Nat Prod 87(5):1407−1415

Wu Z, Li S, Li J, Chen Y, Saurav K, Zhang Q, Zhang H, Zhang W, Zhang W, Zhang S, Zhang C (2013) Antibacterial and cytotoxic new napyradiomycins from the marine-derived *Streptomyces* sp. SCSIO 10428. Mar Drugs 11(6):2113

Wyche TP, Hou Y, Vazquez-Rivera E, Braun D, Bugni TS (2012) Peptidolipins B-F, antibacterial lipopeptides from an ascidian-derived *Nocardia* sp. J Nat Prod 75(4):735−740

Xia K, Shang J, Sun J, Zhu W, Fu P (2023) Expanding the chem. diversity of secondary metabolites produced by two marine-derived enterocin- and wailupemycin-producing *Streptomyces* strains. ACS Omega 8(31):28886−28897

Xin W, Ye X, Yu S, Lian X-Y, Zhang Z (2012) New capoamycin-type antibiotics and polyene acids from marine *Streptomyces fradiae* PTZ0025. Mar Drugs 10:2388−2402

Xu D, Nepal KK, Chen J, Harmody D, Zhu H, McCarthy PJ, Wright AE, Wang G (2018) Nocardiopsistins A-C: New angucyclines with anti-MRSA activity isolated from a marine sponge-derived *Nocardiopsis* sp. HB-J378. Synth Syst Biotechnol 3(4):246−251

Xu J, Nakazawa T, Ukai K, Kobayashi H, Mangindaan REP, Wewengkang DS, Rotinsulu H, Namikoshi M (2008) Tetrahydrobostrycin and 1-deoxytetrahydrobostrycin, two new hexahydroanthrone derivatives, from a marine-derived fungus *Aspergillus* sp. J Antibiot 61(7):415−419

Xu W, Liang Q, Liang Z, Li X (2010) Antimicrobial chemical constituents from three marine sponges. Zhongguo Yaoxue Zazhi 45(5):332−334

Xu X, Dong Y, Yang J, Wang L, Ma L, Song F, Ma X (2024) Secondary metabolites from marine-derived fungus *Penicillium rubens* BTBU20213035. J Fungi 10(6):424

Xu X, Han J, Lin R, Polyak SW, Song F (2019) Two new piperazine-triones from a marine-derived *Streptomycetes* sp. strain SMS636. Mar Drugs 17(3):186

Xu X, Han J, Zhang X, Xu W, Yang J, Song F (2024) Investigation on the chemical constituents of the marine-derived fungus strain *Aspergillus brunneoviolaceus* MF180246. Nat Prod Res 38(8):1369−1374

Xu X, Li J, Zhang K, Wei S, Lin R, Polyak SW, Yang N, Song F (2021) New isocoumarin analogues from the marine-derived fungus *Paraphoma* sp. CUGBMF180003. Mar Drugs 19(6):313

Xu X, Song F, Fan X, Fang N, Shi J (2009) A novel bromophenol from marine red alga *Symphyocladia latiuscula*. Chem Nat Compd 45(6):811−813

Xu X, Zhao S, Wei J, Fang N, Yin L, Sun J (2012) Porric acid D from marine-derived fungus *Alternaria* sp. isolated from Bohai Sea. Chem Nat Compd 47(6):893−895

Xue C, Tian L, Xu M, Deng Z, Lin W (2008) A new 24-membered lactone and a new polyene δ-lactone

from the marine bacterium *Bacillus marinus*. J Antibiot 61(11):668−674

Yang B, He Y, Lin S, Zhang J, Li H, Wang J, Hu Z, Zhang Y (2019) Antimicrobial dolabellanes and atranones from a marine-derived strain of the toxigenic fungus *Stachybotrys chartarum*. J Nat Prod 82(7):1923−1929

Yang F, Hamann MT, Zou Y, Zhang MY, Gong XB, Xiao JR, Chen WS, Lin HW (2012) Antimicrobial metabolites from the Paracel Islands sponge *Agelas mauritiana*. J Nat Prod 75(4):774−778

Yang G, Nenkep VN, Siwe XN, Leutou AS, Feng Z, Zhang D, Choi HD, Kang JS, Son BW (2009) An acetophenone derivative, clavatol, and a benzodiazepine alkaloid, circumdatin A, from the marine-derived fungus *Cladosporium*. Nat Prod Sci 15(3):130−133

Yang G, Sandjo L, Yun K, Leutou AS, Kim G-D, Choi HD, Kang JS, Hong J, Son BW (2011) Flavusides A and B, antibacterial cerebrosides from the marine-derived fungus *Aspergillus flavus*. Chem Pharm Bull 59(9):1174−1177

Yang J, Zhang X, Xu W, Xu X, Song F (2024) A new phenyl 6,7-dihydroxygeranyl ether derivative from a marine-derived fungus strain of *Penicillium arabicum* ZH3-9. Nat Prod Res 38(13):2231−2236

Yang LJ, Peng XY, Zhang YH, Liu ZQ, Li X, Gu YC, Shao CL, Han Z, Wang CY (2020) Antimicrobial and antioxidant polyketides from a deep-sea-derived fungus *Aspergillus versicolor* SH0105. Mar Drugs 18(12):636

Yang L, Quan M-M, Ma Q-Y, Xie Q-Y, Gai C-J, Dai H-F, Hao YE, Zhao Y-X (2024) Antibacterial indole-diterpenoid alkaloids from the marine fungus *Penicillium* sp. ZYX-Z-718. Chem Biodiversity 21(12):e202401871

Yang RY, Li CY, Lin YC, Peng GT, She ZG, Zhou SN (2006) Lactones from a brown alga endophytic fungus (Number ZZF36) from the South China Sea and their antimicrobial activities. Bioorg Med Chem Lett 16(16):4205−4208

Yang SW, Chan TM, Pomponi SA, Chen G, Wright AE, Patel M, Gullo V, Pramanik B, Chu M (2003) A new bicyclic guanidine alkaloid, Sch 575948, from a marine sponge, *Ptilocaulis spiculifer*. J Antibiot 56(11):970−972

Yang XW, Zhang GY, Ying JX, Yang B, Zhou XF, Steinmetz A, Liu YH, Wang N (2013) Isolation, characterization, and bioactivity evaluation of 3-((6-methylpyrazin-2-yl)methyl)-1H-indole, a new alkaloid from a deep-sea-derived actinomycete *Serinicoccus profundi* sp. nov. Mar Drugs 11:33−39

Yao CBF, Schiebel M, Helmke E, Anke H, Laatsch H (2006) Marine bacteria. XXXIII. *Prefluostatin* and new urauchimycin derivatives produced by *Streptomycete* isolates. Z Naturforsch, B: Chem Sci 61(3):320−325

Yi KX, Xie QY, Ma QY, Yang L, Dai HF, Zhao YX, Hao YE (2024) Diverse ansamycin derivatives from the marine-derived *Streptomyces* sp. ZYX-F-97 and their antibacterial activities. Fitoterapia 173:105814

Yi W, Ge ZW, Wu B, Zhang Z (2020) New metabolites from the marine-derived bacterium *Pseudomonas* sp. ZZ820R. Fitoterapia 143:104555

Yi W, Li Q, Song T, Chen L, Li XC, Zhang Z, Lian XY (2019) Isolation, structure elucidation, and antibacterial evaluation of the metabolites produced by the marine-sourced *Streptomyces* sp. ZZ820. Tetrahedron 75(9):1186−1193

Yi W, Newaz AW, Yong K, Ma M, Lian XY, Zhang Z (2022) New hygrocins K-U and streptophenylpropanamide A and bioactive compounds from the marine-associated

Streptomyces sp. ZZ1956. Antibiotics (Basel, Switz.) 11(11):1455

Yong K, Kaleem S, Yi W, Wu B, Zhang Z (2021) New polyhydroxanthones from the marine-associated fungus *Penicillium* sp. ZZ1750. Tetrahedron Lett 81:153354

Youssef DTA, Shaala LA, Genta-Jouve G (2022) Asperopiperazines A and B: antimicrobial and cytotoxic dipeptides from a tunicate-derived fungus *Aspergillus* sp. DY001. Mar Drugs 20(7):451

Yu HB, Gu BB, Wang SP, Cheng CW, Yang F, Lin HW (2017) New diterpenoids from the marine sponge *Dactylospongia elegans*. Tetrahedron 73(47):6657−6661

Yu HB, Hu B, Ning Z, He Y, Men XL, Yin ZF, Jiao BH, Liu XY, Lin HW (2023) Phyllofenones F-M, scalarane sesterterpenes from the marine sponge *Phyllospongia foliascens*. Mar Drugs 21(10):507

Yu X, Yu L, Jiang X (2014) Synthesis, bacteriostatic activity and antifouling capability of acrylamides containing capsaicin moieties. Yingyong Huaxue 31(5):594−599

Zaman Kau, Wu X, Sarotti AM, Cao S (2022) New and bioactive polyketides from Hawaiian marine-derived fungus *Trichoderma* sp. FM652. Nat Prod Res 36(23):5984-5990

Zeng YB, Wang H, Zuo WJ, Zheng B, Yang T, Dai HF, Mei WL (2012) A fatty acid glycoside from a marine-derived fungus isolated from mangrove plant *Scyphiphora hydrophyllacea*. Mar Drugs 10:598−603

Zha X, Li Y, Zhao H, Tan Y, Zhou S (2024) Pyrrolidine, piperazine, and diazinane alkaloids from the marine bacterium strain *Vibrio ruber* ZXR-93. Molecules 29(18):4446

Zhang D, Noviendri D, Nursid M, Yang X, Son BW (2007) 12,13-dihydroxyfumitremorgin C, fumitremorgin C, and brevianamide F, antibacterial diketopiperazine alkaloids from the marine-derived fungus *Pseudallescheria* sp. Nat Prod Sci 13(3):251−254

Zhang D, Yang X, Kang JS, Choi HD, Son BW (2008) Chlorohydroaspyrones A and B, antibacterial aspyrone derivatives from the marine-derived fungus *Exophiala* sp. J Nat Prod 71(8):1458−1460

Zhang D, Yi W, Ge H, Zhang Z, Wu B (2019) Bioactive streptoglutarimides A-J from the marine-derived *Streptomyces* sp. ZZ741. J Nat Prod 82(10):2800−2808

Zhang F, Braun DR, Chanana S, Rajski SR, Bugni TS (2019) Phallusialides A-E, pyrrole-derived alkaloids discovered from a marine-derived *Micromonospora* sp. bacterium using MS-based metabolomics approaches. J Nat Prod 82(12):3432−3439

Zhang H, Khalil ZG, Capon RJ (2011) Fascioquinols A-F: Bioactive meroterpenes from a deep-water southern Australian marine sponge, *Fasciospongia* sp. Tetrahedron 67(14):2591−2595

Zhang K, Zhang X, Lin R, Yang H, Song F, Xu X, Wang L (2022) New secondary metabolites from the marine-derived fungus *Talaromyces mangshanicus* BTBU20211089. Mar Drugs 20(2):79

Zhang L, Li DL, Chen YC, Tao MH, Zhang WM (2012) Study on secondary metabolites of marine fungus *Penicillium* sp. FS60 from the South China Sea. Zhongyaocai 35(7):1091−1094

Zhang R, Wang H, Chen B, Dai H, Sun J, Han J, Liu H (2022) Discovery of anti-MRSA secondary metabolites from a marine-derived fungus *Aspergillus fumigatus*. Mar Drugs 20(5):302

Zhang W, Liu Z, Li S, Lu Y, Chen Y, Zhang H, Zhang G, Zhu Y, Zhang G, Zhang W, Liu J, Zhang C (2012) Fluostatins I-K from the South China Sea-derived *Micromonospora rosaria* SCSIO N160. J Nat Prod 75(11):1937−1943

Zhang W, Yuan Y, Pang X, Liu Y, Zhang X, Liu Q, Wang J (2024) Structurally diverse secondary metabolites from a deep-sea derived *Cladosporium* sp. SCSIO 41318 and their biological evaluation. Chem Biodiversity 21(12):e202401751

Zhang X, Chen L, Chai W, Lian XY, Zhang Z (2017) A unique indolizinium alkaloid streptopertusacin A and bioactive bafilomycins from marine-derived *Streptomyces* sp. HZP-2216E. Phytochemistry (Elsevier) 144:119−126

Zhang X, Dong Y, Liu X, Wang R, Lu J, Song F (2024) New bisabolane-type sesquiterpenoid from *Aspergillus sydowii* BTBU20213012. Nat Prod Res 38(16):2792−2799

Zhang X, Jia R, Yang J, Song Z, Xu X, Song F (2024) A butyrolactone derivative from marine-derived fungal strain *Aspergillus terreus* BTBU20211037. Nat Prod Res 2:1−7

Zhang X, Xu H-Y, Huang AM, Wang L, Wang Q, Cao PY, Yang PM (2016) Antibacterial meroterpenoids from the south China sea sponge *Dysidea* sp. Chem Pharm Bull 64(7):1036−1042

Zhang Y, Li XM, Wang BG (2012) Anthraquinone derivatives produced by marine-derived fungus *Aspergillus versicolor* EN-7. Biosci, Biotechnol, Biochem 76(9):1774−1776

Zhang Y, Li Z, Huang B, Liu K, Peng S, Liu X, Gao C, Liu Y, Tan Y, Luo X (2022) Anti-osteoclastogenic and antibacterial effects of chlorinated polyketides from the Beibu Gulf coral-derived fungus *Aspergillus unguis* GXIMD 02505. Mar Drugs 20(3):178

Zhang Z, Sibero MT, Kai A, Fukaya K, Urabe D, Igarashi Y (2021) TMKS8A, an antibacterial and cytotoxic chlorinated α-lapachone, from a sea slug-derived actinomycete of the genus *Streptomyces*. J Antibiot 74(7):464−469

Zhang Z, Song T, Lian X, Chen M. (2018). CN108913606

Zhao JC, Li XM, Gloer JB, Wang BG (2014) First total syntheses and antimicrobial evaluation of penicimonoterpene, a marine-derived monoterpenoid, and its various derivatives. Mar Drugs 12(6):3352−3370

Zhao R, Li J, Peng H, Li Y, Wang L, Ye B (2012) Isolation and structure elucidation of antibacterial metabolites from marine-derived *Penicillium* strain XGH2321. Zhongguo Kangshengsu Zazhi 37(4):261−264

Zhao W, Jiang H, Zhou J, Xie Y, Lin R, Jiang H. (2022). CN115109023

Zhen X, Gong T, Liu F, Zhang PC, Zhou WQ, Li Y, Zhu P (2015) A new analogue of echinomycin and a new cyclic dipeptide from a marine-derived *Streptomyces* sp. LS298. Mar Drugs 13(11):6947−6961

Zheng CJ, Liao HX, Mei RQ, Huang GL, Yang LJ, Zhou XM, Shao TM, Chen GY, Wang CY (2019) Two new benzophenones and one new natural amide alkaloid isolated from a mangrove-derived Fungus *Penicillium citrinum*. Nat Prod Res 33(8):1127−1134

Zheng CJ, Shao CL, Wang KL, Zhao DL, Wang YN, Wang CY (2012) Secondary metabolites and their bioactivities of a soft coral-derived fungus *Aspergillus versicolor* (ZJ-2008015). Zhongguo Haiyang Yaowu 31(2):7−13

Zheng CJ, Shao CL, Wu LY, Chen M, Wang KL, Zhao DL, Sun XP, Chen GY, Wang CY (2013) Bioactive phenylalanine derivatives and cytochalasins from the soft coral-derived fungus, *Aspergillus elegans*. Mar Drugs 11(6):2054

Zheng M, Huang Z, Zheng Y, Tang Y, Wang J, Yang S (2024) Cotteslosin D, a new cyclopentapeptide from a sponge-derived fungus *Aspergillus versicolour* 2-18. Nat Prod Res 14:1−6

Zheng YY, Liang ZY, Shen NX, Liu WL, Zhou XJ, Fu XM, Chen M, Wang CY (2019) New naphtha-γ-pyrones isolated from marine-derived fungus *Penicillium* sp. HK1-22 and their antimicrobial activities. Mar Drugs 17(6):322

Zhou B, Huang Y, Zhang HJ, Li JQ, Ding WJ (2019) Nitricquinomycins A-C, uncommon naphthopyrrolediones from the *Streptomyces* sp. ZS-A45. Tetrahedron 75(30):3958−3961

Zhou B, Ji YY, Zhang HJ, Shen L (2021) Gephyyamycin and cysrabelomycin, two new angucyclinone derivatives from the *Streptomyces* sp. HN-A124. Nat Prod Res 35(13):2117−2122

Zhou SL, Wang M, Zhao HG, Huang YH, Lin YY, Tan GH, Chen SL (2016) Penicilazaphilone C, a new antineoplastic and antibacterial azaphilone from the marine fungus *Penicillium* sclerotiorum. Arch Pharmacal Res 39(12):1621−1627

Zhou Z, Yang J, Ma J, Shang Z, Fang R, Tian X, Li Q, Ju J (2025) Antarmycins: discovery, biosynthesis, anti-pathogenic bacterial activity, and mechanism of action from deep-sea-derived *Pseudonocardia antarctica*. JACS Au 5(1):237−249

Zhuravleva OI, Oleinikova GK, Antonov AS, Kirichuk NN, Pelageev DN, Rasin AB, Menshov AS, Popov RS, Kim NY, Chingizova EA, Chingizov AR, Volchkova OO, von Amsberg G, Dyshlovoy SA, Yurchenko EA, Guzhova IV, Yurchenko AN (2022) New antibacterial chloro-containing polyketides from the alga-derived fungus *Asteromyces cruciatus* KMM 4696. J. Fungi 8(5):454

Zoraghi R, Worrall L, See RH, Strangman W, Popplewell WL, Gong H, Samaai T, Swayze RD, Kaur S, Vuckovic M, Finlay BB, Brunham RC, McMaster WR, Davies-Coleman MT, Strynadka NC, Andersen RJ, Reiner NE (2011) Methicillin-resistant *Staphylococcus aureus* (MRSA) pyruvate kinase as a target for bis-indole alkaloids with antibacterial activities. J Biol Chem 286(52):44716−44725

Zou Y, Shi J, Sun JL, Li LY, Yan ZY, Guo ZK, Jiao RH (2024) Maduraflavacins A-E, unusual phenyl polyene metabolites produced by a rare marine-derived *Actinomadura* sp. J Nat Prod 87(10):2530−2536

Zubair M, Alarif W, Al-Footy K, Mohamed PH, Ali M, Basaif S, Al-Lihaibi S, Ayyad S-E (2016) New antimicrobial biscembrane hydrocarbon and cembranoid diterpenes from the soft coral *Sarcophyton trocheliophorum*. Turk J Chem. 40(3):385−392
